# First-line tislelizumab and ociperlimab combined with gemcitabine and cisplatin in advanced biliary tract cancer (ZSAB-TOP): a multicenter, single-arm, phase 2 study

**DOI:** 10.1038/s41392-025-02356-y

**Published:** 2025-08-21

**Authors:** Guoming Shi, Xiaoyong Huang, Liang Ma, Hui Li, Jianhong Zhong, Junye Wang, Qiang Gao, Xiaojun Guo, Shuangjian Qiu, Huichuan Sun, Yinghong Shi, Xiaowu Huang, Xiaoying Wang, Yong Yi, Xiaodong Zhu, Cheng Huang, Zhenbin Ding, Yi Chen, Yifeng He, Yinghao Shen, Qiman Sun, Jian Zhou, Jia Fan

**Affiliations:** 1 Department of Hepatobiliary Surgery and Liver Transplantation, Liver Cancer Institute, Zhongshan Hospital, Fudan University, Key Laboratory of Carcinogenesis and Cancer Invasion of Ministry of Education, Shanghai, 200032 China; 2 Department of Hepatobiliary Surgery, Guangxi Medical University Cancer Hospital, Nanning, Guangxi 530021 China; 3 Department of Oncology, The Affiliated Hospital of Jining Medical University, Jining, 272000 China; 4 Department of Hepatic Oncology, Liver Cancer Institute, Zhongshan Hospital, Fudan University, Key Laboratory of Carcinogenesis and Cancer Invasion of Ministry of Education, Shanghai, 200032 China

**Keywords:** Gastrointestinal cancer, Clinical trials

## Abstract

Adding a PD-1/PD-L1 inhibitor to gemcitabine plus cisplatin (GemCis) has shown survival benefits in advanced biliary tract cancer (BTC). Dual inhibition of PD-1/PD-L1 and TIGIT may act synergistically, and further enhance antitumor effects. ZSAB-TOP was a single-arm, multicenter, phase 2 study (NCT05023109) evaluating efficacy and safety of first-line tislelizumab (a PD-1 inhibitor) plus ociperlimab (a TIGIT inhibitor) and GemCis in advanced BTC. Eligible patients received tislelizumab (200 mg) and ociperlimab (900 mg) on day 1 until unacceptable toxicity or disease progression, in combination with cisplatin (25 mg/m²) and gemcitabine (1000 mg/m²) on days 1 and 8 of a 21-day cycle for a maximum eight cycles. The primary endpoint was confirmed objective response rate (ORR) evaluated by the investigator, which was compared with a historical ORR of 25% with GemCis, with a statistical superiority setting at p ≤ 0.05. From March 8, 2022, to January 18, 2023, 45 patients were enrolled. Among the 41 patients in the efficacy analysis set, the confirmed ORR was 51.2% (95% CI 35.1–67.1), achieving the statistical superiority criteria (p = 0.0003). Patients who had TIGIT^+^/PD-L1^+^ (n = 16) tended to have a numerically greater confirmed ORR (75.0% [95% CI 47.6–92.7]). After a median follow-up of 14.6 months, median progression-free survival was 7.7 months (95% CI 6.0–9.4), with a median overall survival of 17.4 months (95% CI 11.7-not reached). Treatment-related adverse events of grade ≥3 occurred in 60.0% of patients; immune-mediated adverse events of any grade was observed in 42.2%, with the majority being grade 1 or 2. In conclusion, first-line tislelizumab and ociperlimab plus GemCis yielded clinically promising tumor response and survival outcomes in advanced BTC and were generally well tolerated without new safety signals.

## Introduction

Biliary tract cancer (BTC) refers to a heterogeneous and rare group of aggressive adenocarcinomas, comprising intrahepatic (ICC), gallbladder cancer (GBC), and extrahepatic cholangiocarcinoma (ECC).^1^ Although BTC represents only 3% of all gastrointestinal cancers, its mortality and incidence are rising worldwide.^1,2^ Most patients are typically identified at a late stage, resulting in limited treatment options and a dismal prognosis, with a median overall survival (OS) reaching only 7.1 months under best supportive care.^1,3^ Gemcitabine combined with cisplatin (GemCis) has constituted the standard first-line regimen for advanced BTC for over a decade.^4^ However, despite treatment with GemCis, the median OS remains below one year, emphasizing the urgent demand for more effective therapies.^5^

In recent years, immunotherapy has emerged as a highly promising approach for various cancers.^6^ In phase 3 KEYNOTE-966 and TOPAZ-1 trials of advanced BTC, adding pembrolizumab or durvalumab to GemCis significantly extended median OS compared with GemCis alone, with reported median OS of 12.7 versus 10.9 months and 12.8 versus 11.5 months, respectively.^7,8^ However, the survival benefit of this combination therapy remains modest, highlighting an ongoing need for novel strategies to increase antitumor efficacy and prolong survival.

T-cell immunoreceptor with immunoglobulin and immunoreceptor tyrosine-based inhibition motif domain (TIGIT), a coinhibitory immune checkpoint receptor, is expressed on multiple tumor-infiltrating immune cells in various cancers, including natural killer (NK) cells and CD8^+^, helper, and regulatory T cells.^9^ In mouse models, coblockade of TIGIT and programmed cell death-1/ligand 1 (PD-1/PD-L1) has synergistically enhanced the effector function of CD8^+^ T cells.^10,11^ The CITYSCAPE and MORPHEUS-liver trials demonstrated that adding tiragolumab (a TIGIT inhibitor) to first-line atezolizumab (a PD-L1 inhibitor) or atezolizumab plus bevacizumab significantly extended median progression-free survival (PFS) and improved objective response rate (ORR) in non-small cell lung cancer (NSCLC) and hepatocellular carcinoma (HCC), respectively, with acceptable safety profiles.^12,13^

We hypothesized that adding a TIGIT inhibitor to the existing first-line chemotherapy plus PD-1/PD-L1 inhibitor regimen could improve outcomes in BTC. Ociperlimab is an innovative humanized monoclonal IgG1 antibody that targets TIGIT with high specificity and affinity. It has also shown effective binding to complement component 1q and all Fcγ receptors, facilitating antibody-dependent cellular cytotoxicity.^14^ A phase 1 trial of ociperlimab plus tislelizumab in patients with advanced solid tumors exhibited a manageable safety profile and promising antitumor activity.^15^ The phase 2 ZSAB-TOP study was designed to evaluate the efficacy and safety of first-line tislelizumab (a PD-1 inhibitor) plus ociperlimab and GemCis in advanced BTC.

## Results

### Patient characteristics

**Fig. 1:**
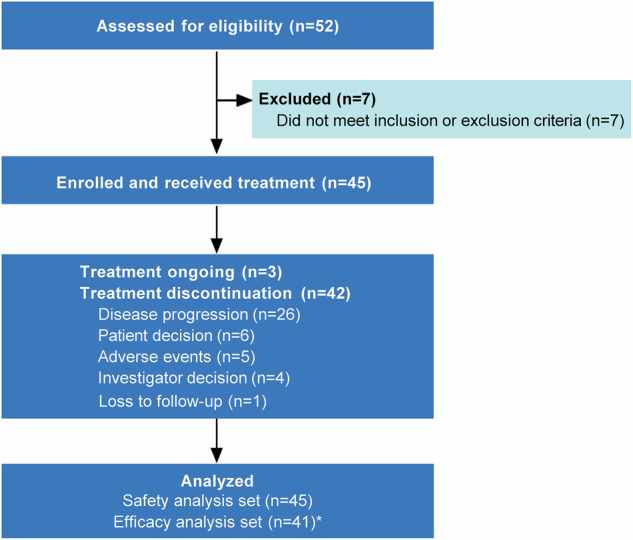
Trial profile. ^*^Four patients were excluded from the efficacy analysis set for the following reasons: one patient withdrew from the study because of loss to follow-up before the first tumor assessment; one withdrew from the study because of noncompliance with study procedures before the first tumor assessment; one discontinued treatment because of an SAE (immune-related myocarditis) before the first tumor assessment and subsequently withdrew from the study; and one discontinued treatment because of cerebral infarction before the first tumor assessment and later participated in another clinical trial

From March 8, 2022, to January 18, 2023, 45 patients were enrolled and included in the safety analysis (SAS; Fig. 1). The median age was 58.0 years (interquartile range [IQR] 53.0--64.0), with males accounting for 55.6% (25/45) of the cohort. Most patients had TNM stage IV disease (31 [68.9%]), had tumors of intrahepatic origin (34 [75.6%]), and had metastatic disease at enrollment (27 [60.0%]). A total of 44 (97.8%) patients were assessed for TIGIT and PD-L1 expression levels. The distribution of patients by TIGIT and PD-L1 status was as follows: 40.0% (18/45) were TIGIT^+^ and PD-L1^+^, 4.4% (2/45) TIGIT^+^ and PD-L1^-^, 28.9% (13/45) TIGIT^-^ and PD-L1^+^, and 24.4% (11/45) TIGIT^-^ and PD-L1^-^. The baseline characteristics of the 45 enrolled patients are detailed in Table 1.

**Table 1:** Demographics and baseline characteristics

	Patients (n = 45)
Age, year	58.0 (53.0-64.0)
Sex	
Male	25 (55.6)
Female	20 (44.4)
Site of origin	
Intrahepatic	34 (75.6)
Extrahepatic	1 (2.2)
Gallbladder	10 (22.2)
Disease classification	
Locally advanced	18 (40.0)
Metastatic	27 (60.0)
Disease status	
Initially, unresectable	39 (86.7)
Recurrent	6 (13.3)
ECOG performance status	
0	41 (91.1)
1	4 (8.9)
TNM disease stage	
II	2 (4.4)
III	12 (26.7)
IV^*^	31 (68.9)
Expression of PD-L1 and TIGIT	
TIGIT^+^ and PD-L1^+^	18 (40.0)
TIGIT^+^ and PD-L1^-^	2 (4.4)
TIGIT^-^ and PD-L1^+^	13 (28.9)
TIGIT^-^ and PD-L1^-^	11 (24.4)
Unknown	1 (2.2)

Data are presented as the median (IQR) or n (%). IQR, interquartile range; ECOG, Eastern Cooperative Oncology Group; TIGIT, T-cell immunoreceptor with immunoglobulin and immunoreceptor tyrosine-based inhibition motif domain; TNM, tumor-node-metastasis; PD-L1, programmed cell death-ligand 1; PD-L1^+^ was defined as the ratio of the area occupied by PD-L1-stained cells (tumor cells, lymphocytes, and macrophages) to the total tumor area (TAP, tumor area positivity) ≥1%; TIGIT^+^ was defined as the percentage of TIGIT-positive immune cells relative to the total immune cell population divided by the tumor area (positive IC/tumor area), with a threshold of ≥1%

^*^including 27 patients with metastatic disease at enrollment and 4 with locally advanced gallbladder cancer (T4N1M0, n = 2; T4N2M0, n = 1; T4N0M0, n = 1)

According to the data cutoff (July 19, 2024), median follow-up was 14.6 months (IQR 9.6–19.5). Patients received a median of 8 (IQR 3.0–12.0) treatment cycles. Median exposure duration was 4.4 months (IQR 1.8–5.5) for gemcitabine, 4.1 months (IQR 1.8–5.5) for cisplatin, and 5.5 months (IQR 2.1–8.3) for tislelizumab and ociperlimab. At the data cutoff, treatment was ongoing in 3 (6.7%) of 45 patients, with 42 (93.3%) having discontinued due to disease progression (26 [57.8%] of 45 patients), patient decision (6 [13.3%]), adverse events (AEs; 5 [11.1%]), investigator decision (4 [8.9%]), or loss to follow-up (1 [2.2%]). In the efficacy analysis set (EAS), 28 (68.3%) of the 41 patients had received subsequent anticancer therapies, primarily immunotherapy (19 [46.3%]), targeted therapy (17 [41.5%]), and chemotherapy (17 [41.5%]; supplementary Table 1).

### Efficacy

**Fig. 2:**
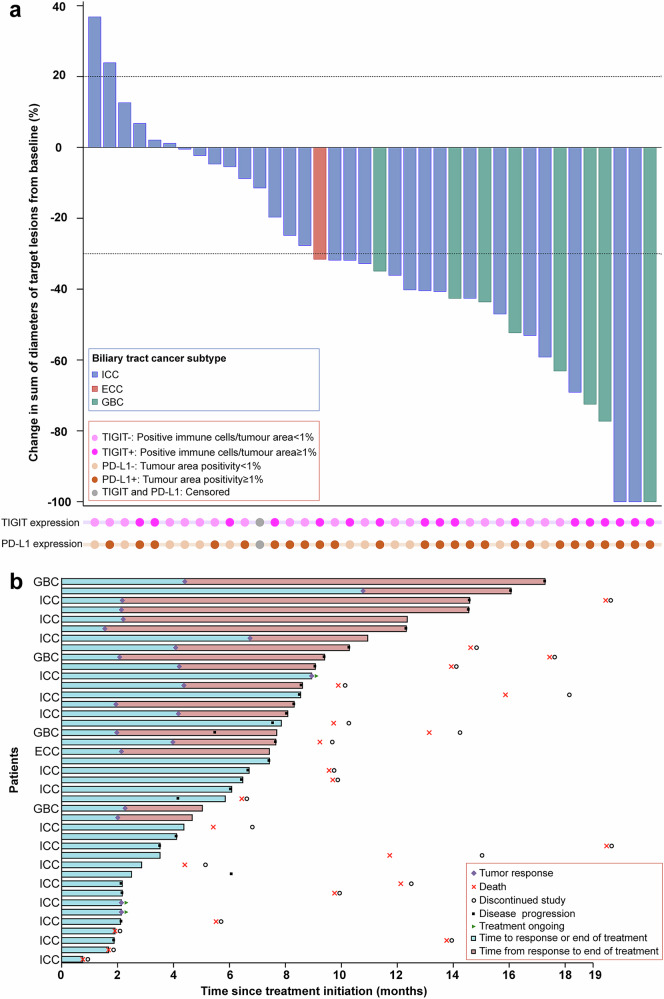
Tumor responses. (**a**) Waterfall plot of the maximum percent change in the size of target lesions from baseline (n = 38). Three patients were excluded because of death before the first tumor response evaluation. (**b**) Treatment duration per Response Evaluation Criteria in Solid Tumors version 1.1 (n = 41). ICC, intrahepatic cholangiocarcinoma; GBC, gallbladder cancer; ECC, extrahepatic cholangiocarcinoma; PD-L1, programmed cell death-ligand 1; TIGIT, T-cell immunoreceptor with immunoglobulin and immunoreceptor tyrosine-based inhibition motif domain

Among the 41 patients in the EAS, the confirmed ORR evaluated by investigator was 51.2% (21 of 41 patients; 95% CI 35.1--67.1), including 3 confirmed complete responses (CRs) and 18 confirmed partial responses (PRs; Table 2 and Fig. 2a), indicating that the combination therapy was significantly superior to the historical control (p = 0.0003). Disease control rate (DCR) was 82.9% (34 of 41 patients; 95% CI 67.9–92.8), with 13 patients exhibiting stable disease (SD; including 2 unconfirmed PR). Among the 23 patients who achieved CR or PR (including 2 unconfirmed PRs), the median duration of response (DoR) was 6.4 months (95% CI 4.2–12.5; supplementary Fig. 1). The time of treatment for all patients in the EAS is shown in Fig. 2b. Three (7.3%) patients achieved obvious tumor regression after treatment, met the criteria for surgical resection, subsequently discontinued treatment and underwent curative surgery.

**Table 2:** Tumor response

	Patients (n = 41)
ORR (confirmed response only), n (% [95% CI])	21 (51.2 [95% CI, 35.1-67.1])
ORR (confirmed and unconfirmed responses), n (% [95% CI])	23 (56.1 [95% CI, 39.7-71.5])
DCR, n (% [95% CI])	34 (82.9 [95% CI, 67.9-92.8])
Best overall response	
CR	3 (7.3)
PR	18 (43.9)
SD^*^	13 (31.7)
PD	4 (9.8)
NE	3 (7.3)

Data are n (%) unless otherwise specified. ORR, objective response rate; DCR, disease control rate; CR, complete response; PR, partial response; SD, stable disease; PD, progressive disease; NE, not evaluable; CI, confidence interval; ^*^including 2 patients with unconfirmed PR

**Fig. 3:**
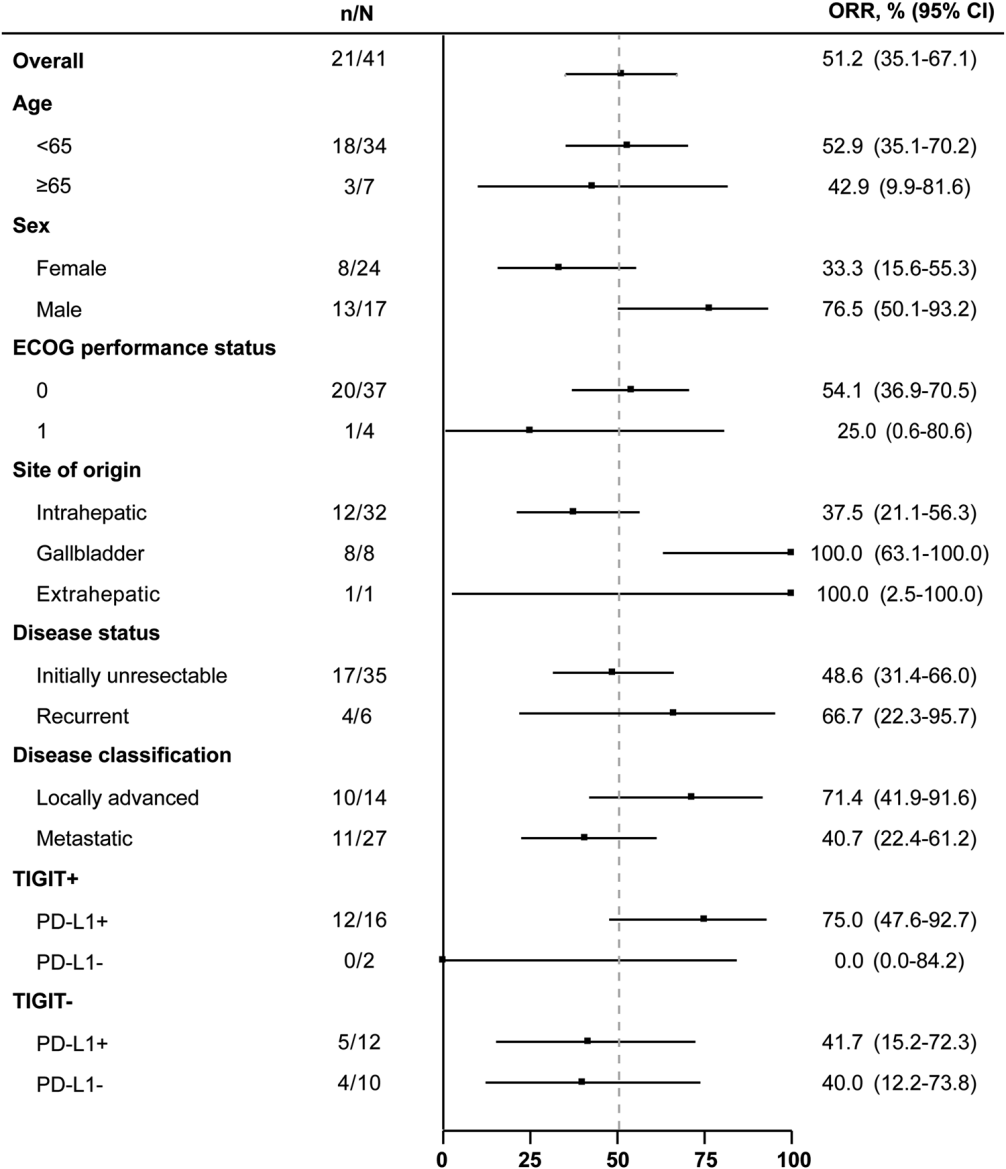
Subgroup analyses for the confirmed ORR in the efficacy analysis set. ORR, objective response rate; ECOG, Eastern Cooperative Oncology Group; PD-L1, programmed cell death-ligand 1; TIGIT, T-cell immunoreceptor with immunoglobulin and immunoreceptor tyrosine-based inhibition motif domain; CI, confidence interval

Patients who were TIGIT^+^ (12 of 18 patients; 66.7% [95% CI 41.0--86.7]) or PD-L1^+^ (17/28; 60.7% [95% CI 40.6--78.5]) presented numerically higher confirmed ORRs than those who were TIGIT^-^ (9/22; 40.9% [95% CI 20.7--63.6]) or PD-L1^-^ (4/12; 33.3% [95% CI 9.9--65.1]). To further explore the combined impact of TIGIT and PD-L1 status, patients were stratified on the basis of both markers. As shown in Fig. 3, patients in the TIGIT^+^ and PD-L1^+^ subgroups (12 of 16 patients; 75.0% [95% CI 47.6–92.7]) tended to have numerically higher confirmed ORRs than those in the TIGIT^+^ and PD-L1^-^ (0/2; 0% [95% CI 0.0–84.2]), TIGIT^-^ and PD-L1^+^ (5/12; 41.7% [95% CI 15.2–72.3]), and TIGIT^-^ and PD-L1^-^ (4/10; 40.0% [95% CI 12.2–73.8]) subgroups. All 3 patients with confirmed CRs were positive for both TIGIT and PD-L1 expression. The confirmed ORR was 37.5% (12 of 32 patients; 95% CI 21.1--56.3) for ICC, 100.0% (8/8; 95% CI 63.1--100.0) for GBC, and 100.0% (1/1; 95% CI 2.5–100.0) for ECC. Expression of TIGIT and PD-L1 across the ICC, ECC, and GBC subgroups is provided in supplementary Table 2. A total of 28.1% (9/32) of patients with ICC were positive for both TIGIT and PD-L1, whereas 100.0% (1/1) of patients with ECC and 75.0% (6/8) of patients with GBC were positive for both TIGIT and PD-L1.

**Fig. 4:**
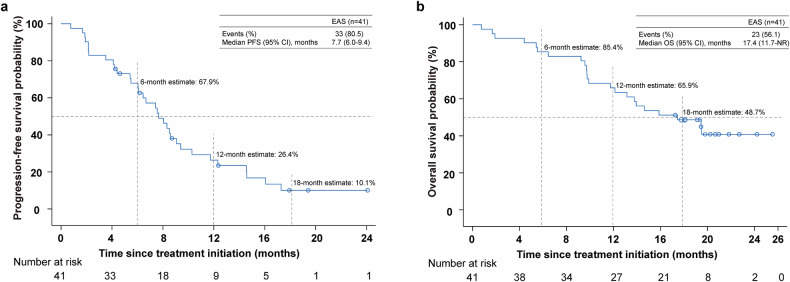
Kaplan‒Meier analyses of survival as assessed by investigators. (**a**) Kaplan‒Meier curves of PFS. (**b**) K‒M curves of OS. PFS, progression-free survival; OS, overall survival; NR, not reached; CI, confidence interval; EAS, efficacy analysis set

At the data cutoff, 33 (80.5%) patients experienced PFS. The median PFS was 7.7 months (95% CI 6.0–9.4), and PFS rate at 12 months was 26.4% (95% CI 13.5–41.3; Fig. 4a). At the data cutoff, 23 (56.1%) patients had died. The median OS was 17.4 months (95% CI 11.7-not reached [NR]), and OS rate at 12 months was 65.9% (95% CI 49.3–78.2; Fig. 4b).

We further evaluated the PFS and OS outcomes based on TIGIT and PD-L1 expression status (Supplementary Fig. 2). Specifically, the median PFS was 8.3 months (95% CI 6.0–14.6) for patients who were TIGIT^+^ versus 7.4 months (95% CI 2.2–9.1) for TIGIT^-^, with median OS of NR (95% CI 17.4-NR) versus 12.9 months (95% CI 9.6–19.5). For patients who were PD-L1^+^ versus PD-L1^-^, the median PFS was 8.3 months (95% CI 6.0--11.7) versus 7.0 months (95% CI 2.1--12.3), and the median OS was 19.5 months (95% CI 12.1-NR) versus 13.8 months (95% CI 6.4-NR), respectively. When TIGIT and PD-L1 status were analyzed as a combined factor, patients with TIGIT^+^ and PD-L1^+^ status had a median PFS of 9.4 months (95% CI 6.0–17.3), numerically greater than that of patients with TIGIT^+^ and PD-L1^-^ status (5.9 months [95% CI 4.2–7.6]), TIGIT^-^ and PD-L1^+^ status (7.7 months [95% CI 1.9–10.3]), and TIGIT^-^ and PD-L1^-^ status (7.0 months [95% CI 1.9–12.3]). Median OS was NR (95% CI 19.5-NR) for patients who were TIGIT^+^ and PD-L1^+^, 8.1 months (95% CI 6.4–9.7) for TIGIT^+^ and PD-L1^-^, 10.8 months (95% CI 1.9–15.9) for TIGIT^-^ and PD-L1^+^, and 16.7 months (95% CI 5.5–NR) for TIGIT^-^ and PD-L1^-^.

### Safety

Treatment-emergent AE (TEAE) of any grade occurred in all patients (100.0%), with 97.8% (44 of 45 patients) reporting treatment-related AEs (TRAEs; supplementary Table 3). Grade ≥3 TRAEs occurred in 60.0% (27/45) of patients. TRAEs of grade ≥3 occurring in at least 10% of patients were neutropenia (13 [28.9%] of 45 patients), leukopenia (12 [26.7%]), thrombocytopenia (8 [17.8%]), and anemia (6 [13.3%]; Table 3). Eleven (24.4%) of 45 patients experienced serious adverse events (SAEs), with 7 (15.6%) experiencing treatment-related SAEs. TRAEs resulted in chemotherapy discontinuation in 7 patients (15.6%) and immunotherapy discontinuation in 3 patients (6.7%). Three patients (6.7%) experienced TEAEs that led to death; none were deemed treatment related.

**Table 3:** Treatment-related adverse events occurring in ≥ 5% of patients in the safety analysis set

Treatment-related adverse events	Any grade	Grade ≥3
Any event	44 (97.8)	27 (60.0)
Anemia	32 (71.1)	6 (13.3)
Leukopenia	31 (68.9)	12 (26.7)
Neutropenia	28 (62.2)	13 (28.9)
Thrombocytopenia	23 (51.1)	8 (17.8)
Elevated AST	15 (33.3)	3 (6.7)
Elevated ALT	14 (31.1)	3 (6.7)
Elevated serum creatinine	14 (31.1)	0
Rash	11 (24.4)	2 (4.4)
Proteinuria	9 (20.0)	0
Vomiting	7 (15.6)	0
Nausea	5 (11.1)	0
Urinary tract infection	5 (11.1)	0
Weight loss	5 (11.1)	0
Hypothyroidism	4 (8.9)	0
Fatigue	4 (8.9)	1 (2.2)
Elevated GGT	4 (8.9)	1 (2.2)
Elevated troponin T	3 (6.7)	0
Elevated serum bilirubin	3 (6.7)	2 (4.4)
Hematuria	3 (6.7)	0
Anorexia	3 (6.7)	0

Data are n (%) unless otherwise specified. AST, aspartate aminotransferase; ALT, alanine aminotransferase; GGT, gamma glutamyl transferase

Immune-mediated AEs of any grade occurred in 19 (42.2%) patients, with dermatitis/rash (9 [20.0%] of 45 patients), hypothyroidism (4 [8.9%]), and increased troponin T (4 [8.9%]) being frequently reported (supplementary Table 4). Five (11.1%) of 45 patients experienced grade ≥3 immune-mediated AEs, including dermatitis/rash (2 [4.4%] of 45 patients), increased serum creatine phosphokinase (1 [2.2%]), thrombocytopenia (1 [2.2%]), autoimmune myocarditis (1 [2.2%]), and thrombosis (1 [2.2%]).

Throughout the study, coronavirus disease 2019 (COVID-19) occurred in 23 (51.1%) patients (any grade, n = 23; grade ≥3, n = 1); none were deemed treatment related. Nineteen patients experienced 20 treatment delays due to COVID-19 positivity or related isolation/lockdown measures, with a median delay of 9.0 days (range 1.0–19.0). Additionally, four patients skipped one treatment cycle because they were positive for COVID-19.

## Discussion

To our knowledge, ZSAB-TOP is the first study evaluating the efficacy and safety of adding a TIGIT inhibitor to the standard combination of a PD-1 inhibitor and GemCis in advanced BTC. Our results demonstrated that the combination of ociperlimab, tislelizumab, and GemCis yielded a remarkable ORR of 51.2%, which was statistically significantly greater than the historical ORR of 25% for GemCis (p = 0.0003),^4,16^ the standard care at the time of the study design of ZSAB-TOP. Additionally, the combination therapy exhibited a clinically promising OS of 17.4 months, indicating that the tumor response to the combination therapy may lead to long-term survival benefits. Dual immunotherapy plus GemCis was generally well tolerated, with an incidence of 60.0% (32/45) for grade ≥3 TRAEs.

Global phase 3 KEYNOTE-966 and TOPAZ-1 trials established pembrolizumab or durvalumab plus GemCis as new first-line standards, but the ORR was only marginally higher (26.7% and 29.0%, respectively) than that of GemCis,^8,17^ reinforcing the significance of the improved ORR observed in ZSAB-TOP with the addition of ociperlimab to standard chemoimmunotherapy. Moreover, the OS observed in our study was also numerically longer than that with durvalumab or pembrolizumab combined with GemCis, with median OS times of 12.8 months and 12.7 months, respectively.^8,17^ However, caution is needed when interpreting these results, with the limitations of cross-sectional comparisons. Specifically, differences in patient disease and demographics characteristics exist between the ZSAB-TOP and KEYNOTE-966 or TOPAZ-1 trials,^8,17^ including a lower median age, better Eastern Cooperative Oncology Group (ECOG) performance status, and fewer patients with metastatic disease, indicating that potentially younger and medically fitter patients with relatively lower disease burdens are included in our study. Nevertheless, the encouraging treatment response and survival outcomes suggest the potential of this combination for advanced BTC, and additional research is required to confirm these findings.

In addition to between-trial differences, which necessitate caution in cross-trial comparisons, one possible explanation for the improved outcomes could be the addition of TIGIT inhibition to the immunotherapy regimen. First, TIGIT inhibitors can directly modulate immune-mediated antitumor activity. Mechanistically, TIGIT inhibits the cytotoxic activity of NK/T cells through competitive antagonism of CD226-driven activation. Through this competition, TIGIT suppresses the activation, migration, and cytotoxic functions of NK/T cells while facilitating their exhaustion. Furthermore, TIGIT may alter T-cell metabolism to increase tumor cell invasion, angiogenesis, and colony formation.^18^ Second, TIGIT has indirect inhibitory effects on the tumor microenvironment. By triggering CD155, TIGIT induces increased interleukin (IL)-10 secretion while reducing IL-12 levels and modulates T-cell proliferation alongside the release of immunostimulatory cytokines, such as interferon-gamma.^18^ On the basis of these two points, inhibiting TIGIT could enhance the cytotoxic activities of NK/T cells. Third, TIGIT inhibitors can act synergistically with PD-1/PD-L1 blockades, enhancing their antitumor activities. TIGIT is frequently coexpressed with PD-1 across various T-cell subsets, and PD-1 inhibition has been reported to elevate TIGIT levels on CD8^+^ T cells by up to 1.5-fold. The increased expression of coinhibitory molecules, including lymphocyte-activation gene 3, TIGIT, and V domain immunoglobulin suppressor of T-cell activation, plays a critical role in T-cell dysfunction and treatment resistance, suggesting that dual TIGIT and PD-1 blockade may improve outcomes over PD-1 monotherapy. Overall, TIGIT inhibitors could enhance NK/T-cell antitumor activity by disrupting the TIGIT signaling pathway. Additionally, the unique properties of tislelizumab may also contribute to these favorable results. Tislelizumab differs from other PD-1 inhibitors by having an engineered Fc region that abolishes antibody-dependent cellular phagocytosis (a potential resistance mechanism to anti-PD-1 therapy), which may promote a high ORR and durable tumor responses.^19–21^ The PFS observed in ZSAB-TOP was numerically comparable to those in the KEYNOTE-966 and TOPAZ-1 trials, potentially due to complexities involved in assessing PFS in patients with BTC. Progression assessment in this population frequently depends on nonradiographic indicators, including serum carbohydrate antigen 19-9 expression, liver function, and biliary obstruction. Thus, PFS evaluated per the Response Evaluation Criteria in Solid Tumors (RECIST) version 1.1 may not fully capture disease progression in patients with BTC.

Recently, dual immunotherapy targeting PD-1/PD-L1 and TIGIT has yielded promising antitumor activity in other solid tumors, including NSCLC and HCC.^12,13^ For example, the CITYSCAPE trial demonstrated that first-line atezolizumab plus tiragolumab led to clinically meaningful improvements in PFS (5.4 months *vs* 3.6 months) and ORR (31.3% *vs* 16.2%) over atezolizumab alone in PD-L1-selected NSCLC.^12^ Notably, Atezolizumab plus tiragolumab showed a marked OS benefit in patients exhibiting high PD-L1 expression (tumor proportion score ≥50%), suggesting that dual blockade with a TIGIT inhibitor may enhance treatment outcomes of PD-1/PD-L1 inhibitors in tumors exhibiting elevated PD-L1 levels.^12^ Conversely, in the phase 3 SKYSCRAPER-02 trial, tiragolumab plus atezolizumab and chemotherapy did not lead to improvements in PFS and OS in treatment-naïve patients with extensive-stage small-cell lung cancer (SCLC), a disease typically associated with lower PD-L1 expression than NSCLC.^22,23^ Although this exploration in SCLC was unsuccessful, it further underscores the possible impact of PD-L1 and TIGIT expression in therapeutic effects. Since PD-1/PD-L1 and TIGIT are upregulated on infiltrating CD8^+^ T cells in BTC, dual immunotherapy is worth exploring in this disease population.^24,25^ Our previous research also found that high PD-L1 and TIGIT dual expression linked to worse OS in patients with ICC (Pei YZ et al., Zhongshan Hospital, Fudan University, unpublished data; supplementary Fig. 3), suggesting that combined anti-TIGIT and PD-L1 therapy may represent a promising approach for treating ICC. In support of this hypothesis, our study revealed that patients who were either TIGIT^+^ or PD-L1^+^ presented numerically higher confirmed ORR, PFS, and OS rates, especially those positive for both markers. Notably, all patients who achieved confirmed CR were in the TIGIT^+^ and PD-L1^+^ subgroups. Consistent with previous reports, our study revealed that TIGIT was associated with PD-L1 expression, with 90% of TIGIT-positive patients also being PD-L1 positive.^26–28^ Overall, coexpression of TIGIT and PD-L1 might help identify better responders to TIGIT and PD-L1 inhibitors plus chemotherapy in BTC. Additional research is necessary to confirm these findings. Currently, several phase 3 trials investigating dual PD-1 and TIGIT blockade in BTC are underway (NCT06467357 and NCT06109779) and may provide further information regarding this combination strategy.

Studies on advanced BTC frequently pool various subtypes together; however, differences in treatment response and prognosis warrant further investigation. Subgroup analyses from the KEYNOTE-966 and TOPAZ-1 trials revealed that immunotherapy combined with chemotherapy yielded comparable efficacy across the ICC, ECC, and GBC subgroups.^8,17^ In the present study, GBC and ECC patients tended to have better tumor responses. All patients with GBC (n = 8) and ECC (n = 1) achieved an objective response, whereas 37.5% of those with ICC achieved an objective response. Interestingly, up to 77.8% of patients with GBC and ECC were positive for both TIGIT and PD-L1, whereas 28.1% of the ICC patients were dual-positive. Although limited by a small sample size, these results suggested that PD-L1 and TIGIT were more likely to be coexpressed in the GBC and ECC subtypes and that dual inhibition of these targets may have synergistic benefits in these subtypes. Further explorations into PD-L1 and TIGIT expression, as well as the efficacy of dual PD-L1 and TIGIT blockade in these subtypes, are warranted.

The combination of tislelizumab, ociperlimab, and GemCis exhibited manageable safety, with grade ≥3 TRAEs in 60.0% of patients, comparable to that in the TOPAZ-1 (62.7%) and KEYNOTE-966 (70.0%) trials.^8,17^ Notably, most of these AEs were controllable through dose discontinuation or appropriate supportive care, and no treatment-related deaths occurred. Hematologic toxicities, such as neutropenia, leukopenia, thrombocytopenia, and anemia, were the most common grade ≥3 TRAEs, aligning with the known profiles of GemCis.^4,16^ Additionally, 42.2% of patients experienced immune-mediated AEs, which was comparable to the percentage of patients receiving dual immunotherapy combining TIGIT and PD-L1 inhibitors (21.9%-54.4%).^15,22^ The most frequently reported immune-mediated AEs included dermatitis/rash, hypothyroidism, and increased troponin T, in line with previous findings for tislelizumab and ociperlimab combination therapy.^15^ Most of these AEs were grade 1–2, with no new safety signals.^20,29,30^ Importantly, only 3 (6.7%) patients discontinued immunotherapy because of TRAEs. Overall, the safety profile of tislelizumab plus ociperlimab and GemCis was probably acceptable for patients with advanced BTC, without increasing the incidence of grade ≥3 TRAEs or treatment discontinuation relative to the TOPAZ-1 and KEYNOTE-966 trials.^8,17^

The current study had several limitations. First, even though ZSAB-TOP was designed with a prespecified statistical hypothesis and sample size calculation, caution was warranted when concluding the superiority of our combination therapy over GemCis, the standard care when study design of ZSAB-TOP, on the basis of its single-arm design. Moreover, although the TOPAZ-1 and KEYNOTE-966 trials exhibited significantly improved PFS and OS with GemCis plus durvalumab or pembrolizumab over GemCis alone, the ORR remained similar. Second, the confirmed ORR, as assessed by the investigator, served as the primary endpoint. However, to minimize potential bias and ensure consistency, response assessments were rechecked by the leading principal investigator at the primary center after evaluations by investigators at each study site. Third, the study was carried out in the context of COVID-19 pandemic, which has impacted patient enrollment and treatment, resulting in challenges such as treatment discontinuation and delays due to constrained access to medical visits, potentially introducing bias. Fourth, the lack of molecular profiling data represents another limitation of our study. Given that molecular alterations such as isocitrate dehydrogenase 1 (*IDH1*) mutations or fibroblast growth factor receptor 2 (*FGFR2*) fusions may influence therapeutic outcomes, the presence of such genomic heterogeneity could have affected the results, particularly in the context of a relatively small cohort.^31^ Furthermore, the study population was exclusively Asian, limiting the generalizability of the findings to other racial and ethnic groups. Therefore, randomized controlled trials with larger and more diverse cohorts are warranted to confirm these findings.

In conclusion, tislelizumab plus ociperlimab and GemCis yielded clinically promising antitumor activity and survival outcomes in patients with advanced BTC, with a manageable safety profile. These findings underscore that this combination strategy may serve as a potential feasible and optimized first-line treatment option for advanced BTC. Prospective validation in large-scale, phase 3 randomized controlled trials is warranted to confirm these results and inform future clinical practice.

## Materials and methods

### Ethics

The protocol received approval from the institutional review board at each participating center, and the study was conducted in line with local regulations, Good Clinical Practice guidelines, and Declaration of Helsinki. Data from this trial are available upon request to regulatory health authorities, the sponsor’s monitors/representatives, and institutional review board or ethics committee members. An independent clinical research organization and a site management organization were responsible for study management and quality monitoring. Regular site visits, data verification, and compliance checks were performed throughout the study. All participants gave written informed consent before enrollment. This study is registered with ClinicalTrials.gov, number NCT05023109.

### Study design and participants

ZSAB-TOP is a single-arm, investigator-initiated, phase 2 trial recruiting patients from 3 centers in China (supplementary data). Eligible patients aged 18–75 years with histologically or cytologically confirmed unresectable advanced BTC (GBC, ICC, or ECC) were included. Additional primary inclusion criteria included at least one measurable target lesion per RECIST version 1.1, an estimated life expectancy of at least 3 months, an Eastern Cooperative Oncology Group (ECOG) performance status of 0 or 1, and adequate organ and bone marrow function. The key exclusion criteria were ampullary cancer or mixed hepatocellular and cholangiocellular carcinoma, previous systemic therapy (such as chemotherapy or immunotherapy) for BTC, known hypersensitivity or allergy reaction to study treatments, history of another malignancy, or active autoimmune disease necessitating systemic treatment. Detailed inclusion and exclusion criteria can be found in the trial protocol.

### Procedures

The treatment regimen consisted of intravenous tislelizumab (200 mg) and ociperlimab (900 mg) given on day 1, plus gemcitabine (1000 mg/m²) and cisplatin (25 mg/m²) administered on days 1 and 8 of a 21-day cycle. GemCis was given for a maximum of eight cycles unless disease progression occurred. Tislelizumab and ociperlimab administration continued until unacceptable toxicity, investigator decision, disease progression, death, or withdrawal of consent, whichever occurred first. Dose reductions for tislelizumab and ociperlimab were not permitted, while temporary dose interruptions of up to 12 weeks were allowed to manage AEs related to tislelizumab or ociperlimab, necessitating a delay for both drugs. If the AEs failed to resolve within 12 weeks, tislelizumab and ociperlimab were permanently discontinued. Patients who discontinued tislelizumab and ociperlimab due to unacceptable toxicity were allowed to continue GemCis, and vice versa. Treatment decision criteria, including interruption, discontinuation, and dose reduction for AEs, are detailed in the study protocol.

Baseline and follow-up tumor assessments were conducted via computed tomography (CT) or magnetic resonance imaging (MRI) of the abdomen, chest, and pelvis every 9 weeks until withdrawal of consent, death, disease progression, loss to follow-up, or study completion, whichever occurred first. Response assessments were performed by investigators at each study site via RECIST version 1.1 and subsequently reviewed by the leading principal investigator at the primary center to ensure consistency. CR or PR was verified by a subsequent CT scan or MRI performed 4–6 weeks following the initial response. Post-treatment survival follow-ups were conducted every 3 months until study completion, death, withdrawal of consent, or loss to follow-up. AEs were recorded during the whole study and for 30 days following the final dose of the study drug (90 days for immune-mediated AEs) or until patients began another anticancer treatment. The severity of the AEs was graded via the National Cancer Institute Common Terminology Criteria for Adverse Events (NCI-CTCAE) version 5.0.

Tumor biopsy samples, either fresh or formalin-fixed and paraffin-embedded, were acquired from all patients for central laboratory evaluation of TIGIT and PD-L1 expression via TIGIT (E5Y1W; #99567S; Cell Signaling Technology, Danvers, MA, USA) and VENTANA PD-L1 (SP263; Roche, Switzerland) antibodies, respectively. The optimal cutoff points for evaluating PD-L1 and TIGIT expression were identified via receiver operating characteristic curve analysis. A sample was considered to be PD-L1 positive (PD-L1^+^) if the tumor area positivity (TAP) was 1% or higher. TAP was calculated as the ratio of the area occupied by PD-L1-stained cells (macrophages, tumor cells, and lymphocytes) to the total tumor area. TIGIT positivity (TIGIT^+^) was calculated as the percentage of TIGIT-stained immune cells relative to total immune cells divided by the tumor area, with a threshold of ≥1%.

### Outcomes

The primary endpoint was investigator-assessed confirmed ORR (proportion of CR/PR) by RECIST version 1.1. The secondary endpoints included the DCR (CR/PR/SD at the best overall response), DoR (time interval from first documented CR/PR to death from any cause or disease progression), PFS (time interval from the first dose of study treatment until death from any cause or disease progression) according to RECIST version 1.1, OS (time interval from the first dose of study treatment until death from any cause), and safety. Exploratory endpoint was association of the clinical response with PD-L1 and TIGIT expression.

### Statistical analysis

At the time of the study design, GemCis was recognized as the standard first-line regimen for unresectable BTC, yielding an ORR of 25% in advanced cases.^4,16^ Sample size was estimated using an exact binomial test with a one-sided alpha of 0.05 and 80% power to test the hypothesis that the combination therapy would increase ORR from 25% to 45%. Accounting for an anticipated 20% dropout rate, the total planned enrollment was set at 45 patients.

Efficacy was assessed in the EAS, defined as patients who received at least one dose of drugs and had at least one posttreatment tumor assessment, unless treatment was discontinued owing to clinical progression or death before the first posttreatment tumor assessment. All patients who received at least one dose of any study drug were included in the SAS for safety evaluation.

The investigator-assessed tumor responses were summarized descriptively, along with their corresponding 95% CIs, which were calculated via the Clopper‒Pearson method. The Kaplan‒Meier method was used to estimate the medians of investigator-assessed PFS, DoR, and OS, and the Brookmeyer‒Crowley method was used to calculate the 95% CIs. Greenwood’s formula was applied to estimate the 95% CIs for the 6- and 12-month PFS and OS rates. Post hoc subgroup analyses of the ORR based on age (<65 years or ≥65 years), ECOG performance status (0 or 1), site of origin (ICC, ECC, or GBC), sex (female or male), disease classification (locally advanced or metastatic), disease status (initially unresectable or recurrent), TIGIT^+^ status (PD-L1^+^ or PD-L1^-^), and TIGIT^-^ status (PD-L1^+^ or PD-L1^-^) were performed descriptively.

Statistical analyses were conducted via SAS Software (version 9.4; SAS Institute Inc., Cary, NC, USA). A p value below 0.05 was regarded as statistically significant.

## Supplementary information

Statistical Analysis Plan

CONSORT_2025_editable_checklist

Sigtrans_Supplementary_Materials

Study protocol

## Data Availability

The datasets supporting the findings of the current study are included in the manuscript and its Supplemental Materials. The datasets are available from the corresponding author upon reasonable request.

## References

[1] Valle, J. W., Kelley, R. K., Nervi, B., Oh, D. Y. & Zhu, A. X. Biliary tract cancer. *Lancet* **397**, 428–444 (2021). 33516341 10.1016/S0140-6736(21)00153-7

[2] Chen, S. et al. Current status, trends, and predictions in the burden of gallbladder and biliary tract cancer in China from 1990 to 2019. *Chin. Med J. (Engl.)* **135**, 1697–1706 (2022). 35984211 10.1097/CM9.0000000000002258 PMC9509182

[3] Ji, J. H. et al. Natural history of metastatic biliary tract cancer (BTC) patients with good performance status (PS) who were treated with only best supportive care (BSC). *Jpn J. Clin. Oncol.* **45**, 256–260 (2015). 25628352 10.1093/jjco/hyu210

[4] Valle, J. et al. Cisplatin plus gemcitabine versus gemcitabine for biliary tract cancer. *N. Engl. J. Med* **362**, 1273–1281 (2010). 20375404 10.1056/NEJMoa0908721

[5] Park, J. O. et al. Gemcitabine Plus Cisplatin for Advanced Biliary Tract Cancer: A Systematic Review. *Cancer Res Treat.* **47**, 343–361 (2015). 25989801 10.4143/crt.2014.308 PMC4509359

[6] Zhao, Y., Yang, M., Feng, J., Wang, X. & Liu, Y. Advances in immunotherapy for biliary tract cancers. *Chin. Med J. (Engl.)* **137**, 524–532 (2024). 37646139 10.1097/CM9.0000000000002759 PMC10932537

[7] Oh, D. Y. et al. Durvalumab or placebo plus gemcitabine and cisplatin in participants with advanced biliary tract cancer (TOPAZ-1): updated overall survival from a randomized phase 3 study. *Lancet Gastroenterol. Hepatol.* **9**, 694–704 (2024). 38823398 10.1016/S2468-1253(24)00095-5

[8] Kelley, R. K. et al. Pembrolizumab in combination with gemcitabine and cisplatin compared with gemcitabine and cisplatin alone for patients with advanced biliary tract cancer (KEYNOTE-966): a randomized, double-blind, placebo-controlled, phase 3 trial. *Lancet* **401**, 1853–1865 (2023). 37075781 10.1016/S0140-6736(23)00727-4

[9] Ge, Z., Peppelenbosch, M. P., Sprengers, D. & Kwekkeboom, J. TIGIT, the Next Step Towards Successful Combination Immune Checkpoint Therapy in Cancer. *Front Immunol.* **12**, 699895 (2021). 34367161 10.3389/fimmu.2021.699895 PMC8339559

[10] Banta, K. L. et al. Mechanistic convergence of the TIGIT and PD-1 inhibitory pathways necessitates coblockade to optimize anti-tumor CD8(+) T cell responses. *Immunity* **55**, 512–526.e519 (2022). 35263569 10.1016/j.immuni.2022.02.005 PMC9287124

[11] Johnston, R. J. et al. The immunoreceptor TIGIT regulates antitumor and antiviral CD8(+) T-cell effector function. *Cancer Cell* **26**, 923–937 (2014). 25465800 10.1016/j.ccell.2014.10.018

[12] Cho, B. C. et al. Tiragolumab plus atezolizumab versus placebo plus atezolizumab as a first-line treatment for PD-L1-selected non-small cell lung cancer (CITYSCAPE): primary and follow-up analyses of a randomized, double-blind, phase 2 study. *Lancet Oncol.* **23**, 781–792 (2022). 35576957 10.1016/S1470-2045(22)00226-1

[13] Finn, R. S. et al. The results from the MORPHEUS-liver study: Phase Ib/II randomized evaluation of tiragolumab (tira) in combination with atezolizumab (atezo) and bevacizumab (bev) in patients with unresectable, locally advanced or metastatic hepatocellular carcinoma (uHCC). *J. Clin. Oncol.* **41**, 4010–4010 (2023).

[14] Chen, X. et al. A Fc-competent anti-human TIGIT blocking antibody BGB-A1217 elicits strong immune responses and potent anti-tumor efficacy in pre-clinical models. *J. Cancer Res* **81**, 1854 (2021). 10.3389/fimmu.2022.828319 PMC8902820 35273608

[15] Frentzas, S. et al. AdvanTIG-105: a phase I dose escalation study of the anti-TIGIT monoclonal antibody ociperlimab in combination with tislelizumab in patients with advanced solid tumors. *J. Immunother. Cancer* **11**, e005829 (2023). 37857528 10.1136/jitc-2022-005829 PMC10603446

[16] Okusaka, T. et al. Gemcitabine alone or in combination with cisplatin in patients with biliary tract cancer: a comparative multicenter study in Japan. *Br. J. Cancer* **103**, 469–474 (2010). 20628385 10.1038/sj.bjc.6605779 PMC2939781

[17] Oh, D. Y. et al. Durvalumab plus Gemcitabine and Cisplatin in Advanced Biliary Tract Cancer. *NEJM Evid.* **1**, EVIDoa2200015 (2022). 38319896 10.1056/EVIDoa2200015

[18] Chu, X., Tian, W., Wang, Z., Zhang, J. & Zhou, R. Coinhibition of TIGIT and PD-1/PD-L1 in Cancer Immunotherapy: Mechanisms and Clinical Trials. *Mol. Cancer* **22**, 93 (2023). 37291608 10.1186/s12943-023-01800-3 PMC10249258

[19] Ren, Z. et al. Tislelizumab in Patients with Previously Treated Advanced Hepatocellular Carcinoma (RATIONALE-208): A Multicenter, Non-Randomized, Open-Label, Phase 2 Trial. *Liver Cancer* **12**, 72–84 (2023). 36872927 10.1159/000527175 PMC9982342

[20] Qin, S. et al. Tislelizumab *vs* Sorafenib as First-Line Treatment for Unresectable Hepatocellular Carcinoma: A Phase 3 Randomized Clinical Trial. *JAMA Oncol.* **9**, 1651–1659 (2023). 37796513 10.1001/jamaoncol.2023.4003 PMC10557031

[21] Zhang, T. et al. The binding of an anti-PD-1 antibody to FcγRΙ has a profound impact on its biological functions. *Cancer Immunol. Immunother.* **67**, 1079–1090 (2018). 29687231 10.1007/s00262-018-2160-x PMC6006217

[22] Rudin, C. M. et al. SKYSCRAPER-02: Tiragolumab in Combination With Atezolizumab Plus Chemotherapy in Untreated Extensive-Stage Small-Cell Lung Cancer. *J. Clin. Oncol.* **42**, 324–335 (2024). 37976444 10.1200/JCO.23.01363 PMC10824371

[23] Acheampong, E. et al. Tumor PD-L1 Expression in Small-Cell Lung Cancer: A Systematic Review and Meta-Analysis. *Cells* **9**, (2020). 10.3390/cells9112393 PMC7693331 33142852

[24] Shi, X. et al. Single-cell atlas of diverse immune populations in the advanced biliary tract cancer microenvironment. *NPJ Precis Oncol.* **6**, 58 (2022). 35982235 10.1038/s41698-022-00300-9 PMC9388673

[25] Qiao, Y. et al. Enhancement of CAR-T-cell activity against cholangiocarcinoma by simultaneous knockdown of six inhibitory membrane proteins. *Cancer Commun. (Lond.)* **43**, 788–807 (2023). 37282786 10.1002/cac2.12452 PMC10354409

[26] Chauvin, J. M. et al. TIGIT and PD-1 impair tumor antigen-specific CD8⁺ T cells in melanoma patients. *J. Clin. Invest* **125**, 2046–2058 (2015). 25866972 10.1172/JCI80445 PMC4463210

[27] Han, H. S. et al. TOX-expressing terminally exhausted tumor-infiltrating CD8(+) T cells are reinvigorated by coblockade of PD-1 and TIGIT in bladder cancer. *Cancer Lett.* **499**, 137–147 (2021). 33249194 10.1016/j.canlet.2020.11.035

[28] Li, X. et al. A Comprehensive Analysis of Key Immune Checkpoint Receptors on Tumor-Infiltrating T Cells From Multiple Types of Cancer. *Front Oncol.* **9**, 1066 (2019). 31709176 10.3389/fonc.2019.01066 PMC6823747

[29] Zhou, C. et al. Tislelizumab Versus Docetaxel in Patients With Previously Treated Advanced NSCLC (RATIONALE-303): A Phase 3, Open-Label, Randomized Controlled Trial. *J. Thorac. Oncol.* **18**, 93–105 (2023). 36184068 10.1016/j.jtho.2022.09.217

[30] Kim, T. W. et al. Anti-TIGIT Antibody Tiragolumab Alone or With Atezolizumab in Patients With Advanced Solid Tumors: A Phase 1a/1b Nonrandomized Controlled Trial. *JAMA Oncol.* **9**, 1574–1582 (2023). 37768658 10.1001/jamaoncol.2023.3867 PMC10540058

[31] Lamarca, A., Barriuso, J., McNamara, M. G. & Valle, J. W. Molecular targeted therapies: Ready for “prime time” in biliary tract cancer. *J. Hepatol.* **73**, 170–185 (2020). 32171892 10.1016/j.jhep.2020.03.007

