# Supplementary material for: First-line tislelizumab and ociperlimab combined with gemcitabine and cisplatin in advanced biliary tract cancer (ZSAB-TOP): a multicenter, single-arm, phase 2 study
Source: Signal Transduct Target Ther. 2025 Aug 21;10:260. doi: 10.1038/s41392-025-02356-y (PMC12368246; doi:10.1038/s41392-025-02356-y)
Supplement: Supplementary file 1 — Statistical Analysis Plan [file 41392_2025_2356_MOESM1_ESM.doc]

**Statistical Analysis Plan**

| **Protocol Title:** | An open-label, multicenter, single-arm exploratory study investigating the efficacy and safety of GP regimen (gemcitabine + cisplatin) in combination with tislelizumab and ociperlimab as first-line treatment for unresectable advanced biliary tract carcinoma (BTC) |
| --- | --- |
| **Protocol Number:** | BGB-A1217-2001-IIT |
| **Investigational Products:** | Tislelizumab, Ociperlimab, Gemcitabine, Cisplatin |
| **Study phase:** | Phase Ⅱ |
| **Sponsor:** | Zhongshan Hospital, Fudan University |
| **Study site:** | Zhongshan Hospital, Fudan University |
| **Version / Date:** | V1.0/2022-11-29 |

| **Version History** | | | |
| --- | --- | --- | --- |
| Version Number | Version Date | Version Description / Revision History | Reason for Revision (if applicable) |
| 1.0 | 2022-11-29 | Initial Version |  |

Content

[1. Introduction 5](#__RefHeading___Toc203035537)

[2. Study overview 5](#__RefHeading___Toc203035538)

[2.1 Study design 5](#__RefHeading___Toc203035539)

[2.2.1 Sample size 5](#__RefHeading___Toc203035540)

[2.2.2 Randomization 5](#__RefHeading___Toc203035541)

[2.2.3 Blinding 6](#__RefHeading___Toc203035542)

[2.2 Study objective and endpoints 6](#__RefHeading___Toc203035543)

[3. Analysis set 6](#__RefHeading___Toc203035544)

[4. Statistical analysis 7](#__RefHeading___Toc203035545)

[4.1 General principles 7](#__RefHeading___Toc203035546)

[4.2 Patients disposition 10](#__RefHeading___Toc203035547)

[4.3 Protocol deviations 11](#__RefHeading___Toc203035548)

[4.4 Demographics and other baseline characteristics analysis 11](#__RefHeading___Toc203035549)

[4.4.1 Demographics and other baseline characteristics 11](#__RefHeading___Toc203035550)

[4.4.2 Medical history 12](#__RefHeading___Toc203035551)

[4.4.3 Prior treatment history 12](#__RefHeading___Toc203035552)

[4.5 Drug exposure and concomitant medications analysis 12](#__RefHeading___Toc203035553)

[4.5.1 Drug exposure analysis 13](#__RefHeading___Toc203035554)

[4.5.2 Concomitant medications analysis 13](#__RefHeading___Toc203035555)

[4.6 Efficacy analysis 14](#__RefHeading___Toc203035556)

[4.6.1 Primary efficacy endpoint 14](#__RefHeading___Toc203035557)

[4.6.2 Secondary efficacy endpoints 15](#__RefHeading___Toc203035558)

[4.6.3 Exploratory efficacy analysis 16](#__RefHeading___Toc203035559)

[4.7 Safety analysis 17](#__RefHeading___Toc203035560)

[4.7.1 AEs 17](#__RefHeading___Toc203035561)

[4.7.2 Laboratory tests 18](#__RefHeading___Toc203035562)

[4.7.3 Vital signs 19](#__RefHeading___Toc203035563)

[4.7.4 12-lead ECGs 19](#__RefHeading___Toc203035564)

[4.7.5 Physical examination 19](#__RefHeading___Toc203035565)

[4.7.6 Other safety events 20](#__RefHeading___Toc203035566)

[4.8 Subgroup analysis 20](#__RefHeading___Toc203035567)

[4.9 Interim analysis 20](#__RefHeading___Toc203035568)

[5. Supporting documents 20](#__RefHeading___Toc203035569)

[6. Appendix 20](#__RefHeading___Toc203035570)

[6.1 Abbreviations 20](#__RefHeading___Toc203035571)

[7. References 21](#__RefHeading___Toc203035572)

# Introduction

The statistical analysis plan (SAP) is intended to describe the statistical methods to be applied in the clinical trial with protocol number of BGB-A1217-2001-IIT. This study aims to evaluate the efficacy and safety of GP regimen (gemcitabine + cisplatin) in combination with tislelizumab and ociperlimab as first-line treatment for unresectable advanced biliary tract carcinoma (BTC).

This SAP is based on the clinical study protocol version 1.0 (dated 16 June 2021) and the electronic Case Report Form (eCRF) version 1.0 (dated 17 February 2022). Any amendments to the protocol or eCRF may necessitate corresponding updates to the SAP.

This SAP must be finalized after approval by the sponsor and before the corresponding database lock.

# Study overview

## Study design

This is an open-label, multicenter, single-arm phase Ⅱ study designed to evaluate the efficacy and safety of GP in combination with tislelizumab and ociperlimab as first-line treatment for unresectable advanced BTC. The study explores the correlation between expression levels of potential biomarkers PD-L1 and TIGIT with disease status, treatment response/prognosis, aiming to inform novel therapeutic strategies and improve outcomes in patients with advanced BTC.

### 2.2.1 Sample size

The sample size is calculated based on the primary endpoint ORR, using an exact binomial distribution method adopted for this single-arm trial. A sample size of 36 patients will provide 80% statistical power to demonstrate statistically significant difference between the ORR of combination regimen (assumed to be 45%, as assessed per Response Evaluation Criteria in Solid Tumors [RECIST] version 1.1) and the historical control ORR of 25% at a one-sided alpha level of 0.05. Assuming a 20% drop-out rate, a total of 45 patients will be enrolled.

### 2.2.2 Randomization

Not applicable.

### 2.2.3 Blinding

Not applicable.

## Study objective and endpoints

| **Study Objective** | **Study Endpoints** |
| --- | --- |
| **Primary:** | |
| - To assess efficacy of GP in combination with tislelizumab and ociperlimab as first-line treatment for unresectable advanced BTC | - Objective response rate (ORR) |
| **Secondary:** | |
| - To assess efficacy and safety of GP in combination with tislelizumab and ociperlimab as first-line treatment for unresectable advanced BTC | - Disease control rate (DCR) - Duration of response (DoR) - Progression-free survival (PFS) - PFS rates at 6 months and at 12 months - Overall survival (OS) - OS rates at 6 months and at 12 months - Incidence, nature, and severity of adverse events (AEs) and serious AEs (SAEs) |
| - **Exploratory Endpoints:**   To explore potential biomarkers that may correlate with clinical efficacy/prognosis | • Correlation between expression levels of potential biomarkers programmed cell death ligand-1 (PD-L1) and T-cell immunoglobulin and ITIM domain (TIGIT) with disease status, response to/prognosis of GP in combination with tislelizumab and ociperlimab as first-line treatment for unresectable advanced BTC. |

# Analysis set

The safety analysis set (SAS) includes all patients who received at least 1 dose of any study treatment (any component for the combination therapy).

The efficacy-evaluable analysis set (EFF) includes all patients who had measurable disease at baseline per RECIST version 1.1 and have at least one evaluable post-baseline tumor assessment unless discontinued treatment due to clinical progression or death prior to the first post-treatment tumor assessment. the efficacy analysis set (EAS) is a subset of the SAS.

# Statistical analysis

## General principles

**Statistical software**

Statistical analyses will be performed using SAS® version 9.4 (or higher).

**Descriptive statistics**

For quantitative variable, descriptive statistics will include the number of patients, mean, standard deviation, median, minimum, maximum, and quartiles (Q1, Q3). Unless otherwise specified, the minimum and maximum values will retain the same number of decimal places as recorded in the original database. The mean and median will be presented with one additional decimal place, and the standard deviation with two additional decimal places compared to the original data. The number of decimal places for all summary statistics will not exceed four digits.

For qualitative variable, descriptive statistics will include frequencies and/or percentages. Percentages will be presented with **one decimal place**. If the frequency is zero, the corresponding percentage will not be reported. When the percentage equals 100, it will be displayed as **100** without decimal places.

For **time-to-event variables**, descriptive statistics will include the number of non-missing observations (N), median, minimum, and maximum. Kaplan–Meier **event rates** will also be provided, where applicable, for specific time-to-event variables.

In statistical tests, p-values ≥ 0.0001 will be reported to four decimal places. P-values less than 0.0001 will be reported as “< 0.0001”, and p-values greater than 0.9999 will be reported as “> 0.9999”.

**Hypothesis testing**

The primary endpoint ORR will be based upon the investigator’s tumor assessments per RECIST version 1.1 from the EAS. The targeted ORR for the combination regimen (GP in combination with tislelizumab and ociperlimab) is 45%, whereas the historical control ORR for gemcitabine plus cisplatin in a similar population was 25%. The null and alternative hypotheses are thus set as follows:

H0: ORR = 25%

Ha: ORR > 25%

A binomial exact test will be performed for hypothesis testing. If the obtained one-sided p-value is ≤ 0.05, it will be concluded that the investigational products produce a statistically significant increase in ORR compared with the historical control, thereby demonstrating superiority of the investigational products.

**Multiplicity adjustment**

Not applicable.

**Baseline definition**

Unless otherwise specified, baseline is defined as the last non-missing measurement before the first dose of the study drug.

**Missing data**

If the event date is missing, the following rules will be applied for imputation:

1) AE dates:

Unless the available partial date clearly indicates that the AE occurred outside the treatment period, it will be considered a treatment-emergent AE (TEAE).

When the start or end date of an AE is partially missing, the missing portion will be imputed to determine whether the event occurred during the treatment period. In cases of uncertainty, the event will be assumed to have occurred during the treatment period. The following rules will be applied for imputing partially missing AE dates:

If the start date of an AE is missing, the following rules will be applied:

- If both the start day and month are missing and the year matches the treatment start year, the date will be imputed as the treatment start date;
- If both the start day and month are missing and the year dose not matches the treatment start year, the date will be imputed as January 1st;
- If the start day is missing and both the month and year match the treatment start month and year, the date will be imputed as the treatment start date;
- If the day is missing and either the month or year differs from the treatment start month and year, the date will be imputed as the first day of that month.

If the year of the start date or the entire start date is missing, no imputation will be performed.

If the end date of AEs partly missing, the following rules will be applied:

- If both the month and day are missing, it will be replaced with December 31st;
- If only the day is missing, the date will be imputed as the last day of that month;
- If the imputed end date is later than the date of death, the end date will be set to the date of death.

If the year of the end date or the entire end date is missing, no imputation will be performed.

2) Concomitant medication dates:

If the available partial date clearly indicates prior medication, it will be classified as prior medication; otherwise, it will be considered concomitant medication.

When the start or end date of administration, treatment, or procedure is partially missing, the missing portion will be imputed to determine whether the administration, treatment, or procedure is classified as prior or concomitant. The following rules will be applied for imputing partially missing administration dates:

If the start date of administration, treatment, or procedure is partially missing, the following rules will be applied:

- If both the start day and month are missing, the date will be imputed as January 1st;
- If only the start day is missing, the date will be imputed as the first day of that month;
- If the imputed start date is later than the date of death, the date will be set to the date of death.

If the end date of administration, treatment, or procedure is partially missing, the following rules will be applied:

- If both the month and day are missing, it will be replaced with December 31st;
- If only the day is missing, the date will be imputed as the last day of that month;
- If the imputed end date is later than the date of death, the end date will be set to the date of death.

If the year of the start date or end date for administration, treatment, or procedure is missing, or if the start date or end date is completely missing, no imputation will be performed.

3) The tumor diagnosis date will be imputed as the earliest possible date according to the following rules:

- If the month is known but the day is missing, the date will be imputed as the 1st day of that month;
- If the year is known but the month is missing, or both the month and day are missing, the date will be imputed as January 1st of that year;
- If the year is missing, no imputation will be performed.

4) The death date will be imputed as the earliest possible date according to the following rules:

- If the start day is missing, and both the month and year match the treatment start month and year, the date will be imputed as the treatment start date;
- If the day is missing and either the month or year differs from the treatment start month and year, the date will be imputed as the first day of that month.

If the year of the start date is missing, or if the start date is completely missing, no imputation will be performed.

If the month is missing, no imputation will be performed. The data will be treated as censored, with the censoring date defined as the subject’s last known date of survival.

## Patients disposition

The number of patients treated and discontinued from study treatments and/or study and those with major protocol deviations will be counted. The primary reason for study treatment and/or study discontinuation will be summarized according to the categories on the eCRF. The end of study status (alive, dead, withdraw consent, or loss to follow‑up) at the cutoff date will be summarized using the data from the eCRF.

Major protocol deviations will be summarized and listed by each category.

## Protocol deviations

Protocol deviations will be analyzed based on all enrolled patients. The protocol deviation listing will be finalized prior to the final database lock.

The number and percentage of patients with major protocol deviations, categorized by deviation type, will be summarized.

A listing of protocol deviations sorted by subject ID will be provided.

## Demographics and other baseline characteristics analysis

Demographics and other baseline characteristics will be summarized in the SAS using descriptive statistics. Continuous variables include age, body weight, vital signs, time since initial cancer diagnosis, and time since advanced/metastatic disease diagnosis; categorical variables include number of prior systemic therapies received, gender, Eastern Cooperative Oncology Group (ECOG) performance status, country, race and metastatic sites. For variables measured multiple times during the treatment cycle, descriptive statistics will be summarized by time point.

### 4.4.1 Demographics and other baseline characteristics

A descriptive analysis will be performed for all demographic and baseline characteristics, including age, sex, ethnicity, body weight, vital signs, time since initial cancer diagnosis and time since diagnosis of advanced/metastatic disease, smoking history, alcohol consumption history, ECOG performance status, and other relevant parameters. A detailed itemized list will be provided. Age (in years) will be calculated as:

Age (years) = (date of informed consent – date of birth + 1)/365.25, rounded down to the nearest whole number. If the date of birth is missing, the age recorded directly on the CRF will be used.

Cancer history will include an assessment of prior surgical procedures, radiotherapy, and drug therapies, along with documentation of the start and end dates, best response, and the reasons for treatment discontinuation. Imaging data obtained prior to study entry may also be collected for investigator assessment. Disease duration (in months) will be calculated as:

Disease duration (months) = (date of informed consent – date of cancer diagnosis+ 1)/30.4375.

Descriptive analysis of baseline characteristics of study patients will be presented in tabular form, including Child-Pugh scores, ECOG performance status, and other relevant clinical parameters.

### 4.4.2 Medical history

Medical history will be analyzed descriptively. Medical history terms will be coded using Medical Dictionary for Regulatory Activities (MedDRA) version 24.0 or higher and summarized by System organ class (SOC) and preferred term (PT).

A detailed listing of medical history will be provided.

### 4.4.3 Prior treatment history

Prior treatment history includes prior meaningful procedures and prior medications.

Prior medications are defined as any medications that ended before the first dose of the study drug.

Patients enrolled in this study must not have received prior systemic therapy for BTC, including chemotherapy and immunotherapy with PD-1, PD-L1/L2, CTLA-4, TIGIT, or other immune checkpoint inhibitors.

Prior tumor treatments will be analyzed descriptively. Prior medications will be coded using the World Health Organization (WHO) Drug Dictionary (version September 1, 2021 or later), and classified by Anatomical Therapeutic Chemical Classification System, level 2 (ATC2) and preferred name (PN). If an ATC2 code is unavailable for a drug, the ATC level 1 (ATC1) code will be used as a substitute.

A detailed listing of prior treatment history will be provided.

## Drug exposure and concomitant medications analysis

This section encompasses subject drug exposure, treatment compliance, and concomitant medication analysis.

Analyses of drug exposure and concomitant medication will be conducted utilizing the SAS.

### 4.5.1 Drug exposure analysis

A descriptive analysis of subject drug exposure will be conducted, including the number of treatment cycles received (number and percentage of patients), duration of exposure, actual cumulative dose (mg), actual dose intensity (mg/cycle), relative dose intensity, and compliance. The definitions are as follows:

- Duration of drug exposure (cycles) = number of completed treatment cycles (one cycle is defined as 21 days);
- Actual cumulative dose (mg) = total administered dose across all cycles;
- Actual dose intensity (mg or mg/m² per cycle) = actual cumulative dose (mg or mg/m²)/duration of exposure (cycles);
- Relative dose intensity (%) = actual dose intensity (mg per cycle)/initial planned dose intensity (mg/cycle) × 100%; The initial planned dose intensity is based on the protocol-specified planned dose;
- Compliance (%) = actual cumulative dose (mg)/planned cumulative dose (mg) × 100%; The planned cumulative dose refers to the investigator-adjusted planned dose recorded in the Case Report Form (CRF).

A summary will be provided of the number (and percentage) of patients who required dose reduction, treatment interruption, dose delay, or treatment discontinuation of each investigational drug due to AEs or other reasons.

A patient-level listing including all dosing records and calculated summary statistics will be provided.

Actual dose administered per cycle and treatment compliance will be referenced from CRF records.

A detailed listing of drug exposure will be supplied.

### 4.5.2 Concomitant medications analysis

Concomitant medications will be analyzed descriptively, and detailed listings will be provided. Drug names will be coded using the WHO Drug Dictionary (version September 1, 2021 or later), and classified by both ATC2 and PN. If an ATC2 code is unavailable for a given drug, the ATC1 code will be used as a substitute.

Concomitant medications will be defined as medications that (1) started before the first dose of study treatment and are continuing at the time of the first dose of study treatment, or (2) started on or after the date of the first dose of study treatment up to 30 days after the patient’s last dose.

Detailed listings of concomitant medications and concomitant therapies will be provided.

## Efficacy analysis

The efficacy analysis will be primarily based on the EFF.

### 4.6.1 Primary efficacy endpoint

The primary efficacy endpoint is the ORR assessed by investigators according to the RECIST version 1.1. ORR is defined as the proportion of patients achieving a predefined reduction in tumor burden that is maintained for a minimum specified duration, representing the sum of complete response (CR) rate and partial response (PR) rate following treatment with the study drug. The targeted ORR for the combination regimen is 45%, whereas the historical control ORR for gemcitabine plus cisplatin in a similar population was 25%. The null and alternative hypotheses are thus set as follows:

H0: ORR = 25%

Ha: ORR > 25%

A binomial exact test will be performed for hypothesis testing. If the obtained one-sided p-value is ≤ 0.05, it will be concluded that the investigational products produce a statistically significant increase in ORR compared with the historical control, thereby demonstrating superiority of the investigational products.

ORR will be calculated as the number and percentage of responders, with two-sided 90% confidence intervals (CIs) estimated using the exact binomial probability method.

The primary efficacy analysis will be conducted approximately 12 months after the enrollment of the last patient and will be based on the EAS.

### 4.6.2 Secondary efficacy endpoints

#### 4.6.2.1 DCR

DCR is defined as the proportion of patients who achieve tumor shrinkage or stabilization maintained for a minimum duration, including cases of CR, PR, and stable disease (SD), primarily in the context of solid tumors.

DCR will be calculated as the number and percentage of patients with disease control, and exact 95% CIs will be calculated using the binomial distribution within the EAS.

#### 4.6.2.2 DoR

DoR is defined as the time from the first documented response (CR or PR) to progressive disease (PD) or death, whichever occurs first. For patients without documented progression or death as of the data cutoff date, DoR will be censored at the date of the last tumor assessment by imaging.

DoR (months) = (date of progression/death or censoring − date of first response + 1)/30.4375.

The median DoR will be estimated using the Kaplan–Meier method, with 95% CIs constructed using the Brookmeyer and Crowley method, and a corresponding survival curve will be generated.

#### 4.6.2.3 PFS and PFS rate

PFS is defined as the time from initiation of study treatment to the first occurrence of investigator-assessed disease progression or death from any cause, whichever occurs first. For patients without documented disease progression or death as of the data cutoff date, PFS will be censored at the date of the last tumor assessment by imaging. Censoring rules for PFS follow the guidelines described in Appendices A-D in the guidance for industry Clinical Trial Endpoints for the Approval of Non-Small Cell Lung Cancer Drugs and Biologics^[1]^. The censoring rules are outlined in the table below.

**Table: Censoring scheme for PFS**

| **Situation** | **Date of Progression or Censoring** | **Outcome** |
| --- | --- | --- |
| Incomplete or no baseline tumor assessments | Date of treatment start | Censored |
| Progression documented between two assessment visits | Earliest of:   1. Date of new lesion appearance 2. Date of first tumor assessment with documented progression | Progressed |
| No progression | Date of last tumor assessment with no documented progression | Censored |
| Treatment discontinuation for undocumented progression | Date of last tumor assessment with no documented progression | Censored |
| New anticancer treatment started | Date of last tumor assessment with no documented progression before start of new treatment | Censored |
| Death after more than one missed visit | Date of last tumor assessment with no documented progression | Censored |
| Death between two assessment visits | Date of death | Progressed |

PFS (in months) = (date of death/progression or censoring − date of first study treatment + 1)/30.4375.

The median PFS will be estimated using the Kaplan–Meier method, with a 95% CI constructed using the Brookmeyer and Crowley method (if estimable). A Kaplan–Meier survival curve will be generated. In addition, the 6-month and 12-month PFS rates (i.e., the proportion of patients alive and progression-free at 6 and 12 months after initiation of study treatment) will be estimated using the Kaplan–Meier method, with corresponding 95% CIs constructed using Greenwood’s formula.

#### 4.6.2.4 OS and OS rate

OS is defined as the time from the start of study treatment to death from any cause. For patients who are alive at the data cutoff date, OS will be censored at the date of the last survival follow-up.

OS (months) = (date of death or censoring − date of first study treatment + 1)/30.4375.

The median OS will be estimated using the Kaplan–Meier method, with 95% CIs constructed using the Brookmeyer and Crowley method (if estimable). A Kaplan–Meier survival curve will be generated. The 6-month and 12-month OS rates (i.e., the proportion of patients alive at 6 and 12 months after treatment initiation) will also be estimated using the Kaplan–Meier method, with corresponding 95% CIs constructed using Greenwood’s formula.

### 4.6.3 Exploratory efficacy analysis

#### 4.6.3.1 Potential biomarkers correlated with prognosis

The correlation between expression levels of potential biomarkers PD-L1 and TIGIT with disease status, response to/prognosis of GP in combination with tislelizumab and ociperlimab as first-line treatment for unresectable advanced BTC will be analyzed using multivariate regression analysis in the EAS.

Subgroup analyses may be conducted based on biomarker expression levels (e.g., PD-L1 and TIGIT expression), to explore differences in efficacy among groups with distinct biomarker profiles, including dual high expression (PD-L1^+^/TIGIT^+^), dual low expression (PD-L1^−^/TIGIT^−^), and single high expression (PD-L1^+^/TIGIT^−^ or PD-L1^−^/TIGIT^+^).

## Safety analysis

This section presents the safety analysis of patients, including AEs, laboratory tests, vital signs, electrocardiograms (ECGs), physical examinations, and other safety events, primarily analyzed using the SAS.

### 4.7.1 AEs

The AE verbatim descriptions (investigator’s description from the eCRF) will be coded using MedDRA. AEs will be coded to MedDRA (version 24.0 or higher) by the PT, and primary SOC.

A TEAE is defined as any AE or SAE that has an onset date or a worsening in severity from baseline (pretreatment) on or after the date of first dose of study treatment up to 30 days following study treatment discontinuation (safety follow-up visit) or initiation of a new anticancer therapy, whichever occurs first.

The causality of each AE should be assessed and classified by the investigator as “related” or “not related”. Those “related” AEs are considered related to the investigational drug.

Immune-related AE (irAE): If alternative causes have been ruled out, the AE requires the use of systemic steroids, other immunosuppressants, or endocrine therapy, and is consistent with an immune-related mechanism of action, the event is considered an irAE.

A summary table of TEAEs will report the number (percentage) of patients experiencing AEs in the following categories.

- All TEAEs
- TEAE related to the study product (ADR)
- irAE
- ≥ Grade 3 TEAE
- ≥ Grade 3 TEAE related to the study product
- TEAEs leading to study withdrawal
- TEAEs leading to treatment interruption
- TEAE leading to dose reduction
- TEAEs leading to treatment discontinuation
- SAE
- SAE related to the study product
- TEAE leading to death
- ADR leading to death

A patient is counted only once by the highest severity grade per National Cancer Institute Common Terminology Criteria for Adverse Events (NCI-CTCAE) version 5.0, within an SOC and PT, even if the patient experiences more than 1 TEAE within a specific SOC and PT.

The number and percentage of patients experiencing TEAEs or adverse reactions, irAEs, SAEs or serious adverse reactions, and TEAEs or adverse reactions leading to study discontinuation will be summarized by SOC and PT.

Similarly, the number and percentage of patients experiencing TEAEs or adverse reactions, irAEs, SAEs or serious adverse reactions, and TEAEs or adverse reactions leading to study discontinuation, stratified by severity grade, will also be summarized by SOC and PT.

Detailed listings will be provided for TEAEs, adverse reactions, irAEs, TEAEs of grade ≥ 3 per CTCAE, and SAEs.

### 4.7.2 Laboratory tests

Clinical laboratory (e.g, hematology, serum chemistry) values will be evaluated as appropriate. Descriptive summary statistics (e.g, n, mean, standard deviation, median, minimum, and maximum for continuous variables; n [%] for categorical variables) for laboratory parameters and their changes from baseline will be calculated. Laboratory values will be summarized by visit and by the worst post-baseline visit.

For continuous laboratory test data, descriptive summary statistics of post-treatment observed values and changes relative to baseline will be provided by visit. A contingency table will be generated to summarize clinical significance assessments (clinically significant abnormality, non-clinically significant abnormality, or normal) before and after treatment. The worst post-treatment assessment will be used, including only patients with non-missing baseline and at least one post-baseline observation.

Laboratory parameters that are graded in NCI-CTCAE version 5.0 will be summarized by NCI-CTCAE Grade. In the summary of laboratory parameters by the NCI-CTCAE Grade, parameters with NCI-CTCAE grading in both high and low directions will be summarized separately.

A detailed listing of laboratory test results will be provided.

### 4.7.3 Vital signs

Vital signs include body temperature, heart rate, respiratory rate, systolic blood pressure, and diastolic blood pressure.

Descriptive summary statistics of post-treatment observed values and changes from baseline will be provided by visit, along with tabulated listings.

### 4.7.4 12-lead ECGs

A contingency table will be generated to summarize the clinical significance assessments (clinically significant abnormality, non-clinically significant abnormality, or normal) of 12-lead ECGs before and after treatment. The worst post-treatment assessment will be used. Only patients with non-missing baseline and at least one post-baseline observation will be included in the analysis.

A detailed listing of 12-lead ECG results will be provided.

### 4.7.5 Physical examination

A contingency table will be generated to summarize physical examination findings (normal or abnormal) before and after treatment. The worst post-treatment assessment will be used. Only patients with non-missing baseline and at least one post-baseline observation will be included in the analysis.

A comprehensive list of all patients with physical examination will be provided.

### 4.7.6 Other safety events

The following safety events will be listed/provided:

- Pregnancy tests
- Pulmonary function tests
- Thyroid function tests
- Virology tests (HBV, HCV, HIV, and syphilis)

## Subgroup analysis

Not applicable.

## Interim analysis

Not applicable.

# Supporting documents

# Appendix

## Abbreviations

| **Abbreviation** | **Definition** |
| --- | --- |
| AE | Adverse event |
| BTC | Biliary tract carcinoma |
| Child-Pugh | Child-Pugh liver function classification |
| CR | Complete response |
| DCR | Disease control rate |
| DoR | Duration of response |
| ECOG | Eastern Cooperative Oncology Group |
| eCRF | Electronic case report form |
| EFF | Efficacy-evaluable analysis set |
| NCI-CTCAE | National Cancer Institute Common Terminology Criteria for Adverse Events |
| ORR | Objective response rate |
| OS | Overall survival |
| PD | Progressive disease |
| PFS | Progression-free survival |
| PR | Partial response |
| PN | Preferred name |
| PT | Preferred term |
| SAE | Serious adverse event |
| SAP | Statistical Analysis Plan |
| SD | Stable disease |
| SAS | Safety analysis set |
| SOC | System organ class |
| TEAE | Treatment emergent adverse event |
| TFL | Tables, figures, and listings |
| WHO Drug Dictionary | World Health Organization Drug Dictionary |
| MedDRA | Medical Dictionary for Regulatory Activities |

# References

[1] Guidance for industry Clinical Trial Endpoints for the Approval of Non-Small Cell Lung Cancer Drugs and Biologics. FDA https://www.fda.gov/media/116860/download
