# Supplementary material for: First-line tislelizumab and ociperlimab combined with gemcitabine and cisplatin in advanced biliary tract cancer (ZSAB-TOP): a multicenter, single-arm, phase 2 study
Source: Signal Transduct Target Ther. 2025 Aug 21;10:260. doi: 10.1038/s41392-025-02356-y (PMC12368246; doi:10.1038/s41392-025-02356-y)
Supplement: Supplementary file 3 — Sigtrans_Supplementary_Materials [file 41392_2025_2356_MOESM3_ESM.docx]

Supplementary Materials for

First-line tislelizumab and ociperlimab combined with gemcitabine and cisplatin in advanced biliary tract cancer (ZSAB-TOP): a multicenter, single-arm, phase 2 study

Guoming Shi^#, *^, Xiaoyong Huang^#^, Liang Ma^#^, Hui Li^#^, Jianhong Zhong, Junye Wang, Qiang Gao, Xiaojun Guo, Shuangjian Qiu, Huichuan Sun, Yinghong Shi, Xiaowu Huang, Xiaoying Wang, Yong Yi, Xiaodong Zhu, Cheng Huang, Zhenbin Ding, Yi Chen, Yifeng He, Yinghao Shen, Qiman Sun, Jian Zhou^*^, Jia Fan^*^

^#^ Guoming Shi, Xiaoyong Huang, Liang Ma, and Hui Li contributed equally as first authors.

^*^Correspondence to: fan.jia@zs-hospital.sh.cn; zhou.jian@zs-hospital.sh.cn; shi.guoming@zs-hospital.sh.cn.

**This PDF file includes:**

List of investigators

Tables S1 to S4

Figures S1 to S3

**Other Supplementary Materials for this manuscript include the following:**

Study protocol

## List of investigators

|  | Investigators | Affiliation | Patients enrolled^*^ |
| --- | --- | --- | --- |
| 1 | Jia Fan/Guoming Shi | Zhongshan Hospital, Fudan University | 38 |
| 2 | Liang Ma | Guangxi Medical University Cancer Hospital | 4 |
| 3 | Junye Wang | The Affiliated Hospital of Jining Medical University | 3 |

^*^Patients were strictly screened and enrolled according to the inclusion and exclusion criteria. Given the tendency of Chinese BTC patients to seek care at high-volume tertiary centers, Zhongshan Hospital—a nationally recognized BTC treatment center—had the highest patient flow and the largest number of eligible patients.

## Table S1. Summary of subsequent anticancer therapies during survival follow-up in the efficacy analysis set

|  | Patients (n=41) |
| --- | --- |
| Subsequent anticancer therapies | 28 (68.3) |
| Immunotherapy | 19 (46.3) |
| Toripalimab | 7 (17.1) |
| Pembrolizumab | 3 (7.3) |
| Sintilimab | 3 (7.3) |
| Adebrelimab | 2 (4.9) |
| Camrelizumab | 2 (4.9) |
| Tislelizumab | 2 (4.9) |
| HS-20089 | 1 (2.4) |
| Envafolimab | 1 (2.4) |
| Cadonilimab | 1 (2.4) |
| Targeted therapy | 17 (41.5) |
| Lenvatinib | 15 (36.6) |
| Gunagratinib | 1 (2.4) |
| Pemigatinib | 1 (2.4) |
| Regorafenib | 1 (2.4) |
| IBI360 | 1 (2.4) |
| Chemotherapy | 17 (41.5) |
| Gemcitabine | 7 (17.1) |
| Capecitabine | 6 (14.6) |
| Nab-paclitaxel | 5 (12.2) |
| S-1 | 4 (9.8) |
| Oxaliplatin | 3 (7.3) |
| Cisplatin | 1 (2.4) |
| Traditional Chinese medicine | 1 (2.4) |
| Surgery | 3 (7.3) |
| Radiotherapy | 1 (2.4) |
| Transarterial chemoembolization | 1 (2.4) |
| Unknown | 1 (2.4) |

Data are n (%).

## Table S2. Expression of TIGIT and PD-L1 across ICC, GBC, and ECC subgroups in the efficacy analysis set

|  | ICC (n=32) | GBC (n=8) | ECC (n=1) |
| --- | --- | --- | --- |
| TIGIT^+^ and PD-L1^+^ | 9 (28.1) | 6 (75.0) | 1 (100.0) |
| TIGIT^+^ and PD-L1^-^ | 2 (6.3) | 0 | 0 |
| TIGIT^-^ and PD-L1^+^ | 10 (31.3) | 0 | 0 |
| TIGIT^-^ and PD-L1^-^ | 10 (31.3) | 2 (25.0) | 0 |
| Unknown | 1 (3.1) | 0 | 0 |

Data are n (%). ICC, intrahepatic cholangiocarcinoma; ECC, extrahepatic cholangiocarcinoma; GBC, gallbladder cancer; PD-L1, programmed cell death-ligand 1; TIGIT, T-cell immunoreceptor with immunoglobulin and immunoreceptor tyrosine-based inhibition motif domain; PD-L1^+^ was defined as the ratio of the area occupied by PD-L1 staining cells (tumour cells, lymphocytes, and macrophages) to the total tumour area (TAP, tumour area positivity) ≥1%; TIGIT^+^ was defined as the percentage of TIGIT staining immune cells relative to the total immune cell, divided by the tumour area (positive IC/tumour area), with a threshold of ≥1%.

## Table S3. Summary of adverse events in the safety analysis set

|  | Patients (n=45) |
| --- | --- |
| TEAEs |  |
| Any grade | 45 (100.0) |
| Grade ≥3 | 32 (71.1) |
| Serious TEAEs | 11 (24.4) |
| TEAEs leading to discontinuation of chemotherapy | 10 (22.2) |
| TEAEs leading to discontinuation of immunotherapy | 7 (15.6) |
| TEAEs leading to death | 3 (6.7)^*^ |
| TRAEs |  |
| Any grade | 44 (97.8) |
| Grade ≥3 | 27 (60.0) |
| Serious TRAEs | 7 (15.6) |
| TRAEs leading to discontinuation of chemotherapy | 7 (15.6) |
| TRAEs leading to discontinuation of immunotherapy | 3 (6.7) |
| TRAEs leading to death | 0 (0) |

Data are n (%) unless otherwise specified. TEAEs, treatment-emergent adverse events; TRAEs, treatment-related adverse events.

^*^Three patients experienced TEAEs leading to death, all of which were assessed as non-treatment-related mortalities:

1) Cerebral infarction: Prior to the first dose of the study drugs, the patient exhibited early signs of cerebral infarction. The investigator assessed that the cerebral infarction was a consequence of the patient’s pre-existing condition rather than the study drugs.

2) Multiple organ failure: The patient developed symptoms of multiple organ failure one month after the last study drug administration, during which no study medication was given. The investigator attributed the cause of death to the progression of the underlying disease rather than the study drugs.

3) Delayed treatment for ascites caused by coronavirus disease 2019 (COVID-19) pandemic: The patient experienced mild ascites symptoms approximately one month after the first cycle of treatment, and ultimately died due to the lack of timely medical intervention resulting from COVID-19-related quarantine. Given that no study drug was administered during this period, the investigator concluded that the patient's condition and delayed treatment, rather than the study drugs, contributed to the fatal outcome.

## Table S4. Immune-mediated adverse events in the safety analysis set

|  | Patients (n=45) | |
| --- | --- | --- |
|  | Any grade | Grade ≥ 3 |
| Total | 19 (42.2) | 5 (11.1) |
| Immune-mediated adverse events |  |  |
| Dermatitis/rash | 9 (20.0) | 2 (4.4) |
| Hypothyroidism | 4 (8.9) | 0 (0.0) |
| Troponin T increased | 4 (8.9) | 0 (0.0) |
| Hyperthyroidism | 2 (4.4) | 0 (0.0) |
| Serum creatine phosphokinase increased | 2 (4.4) | 1 (2.2) |
| Hypersensitivity | 2 (4.4) | 0 (0.0) |
| Thrombocytopenia | 1 (2.2) | 1 (2.2) |
| Autoimmune myocarditis | 1 (2.2) | 1 (2.2) |
| Thrombosis | 1 (2.2) | 1 (2.2) |
| Euthyroid sick syndrome | 1 (2.2) | 0 (0.0) |

Data are n (%).

*
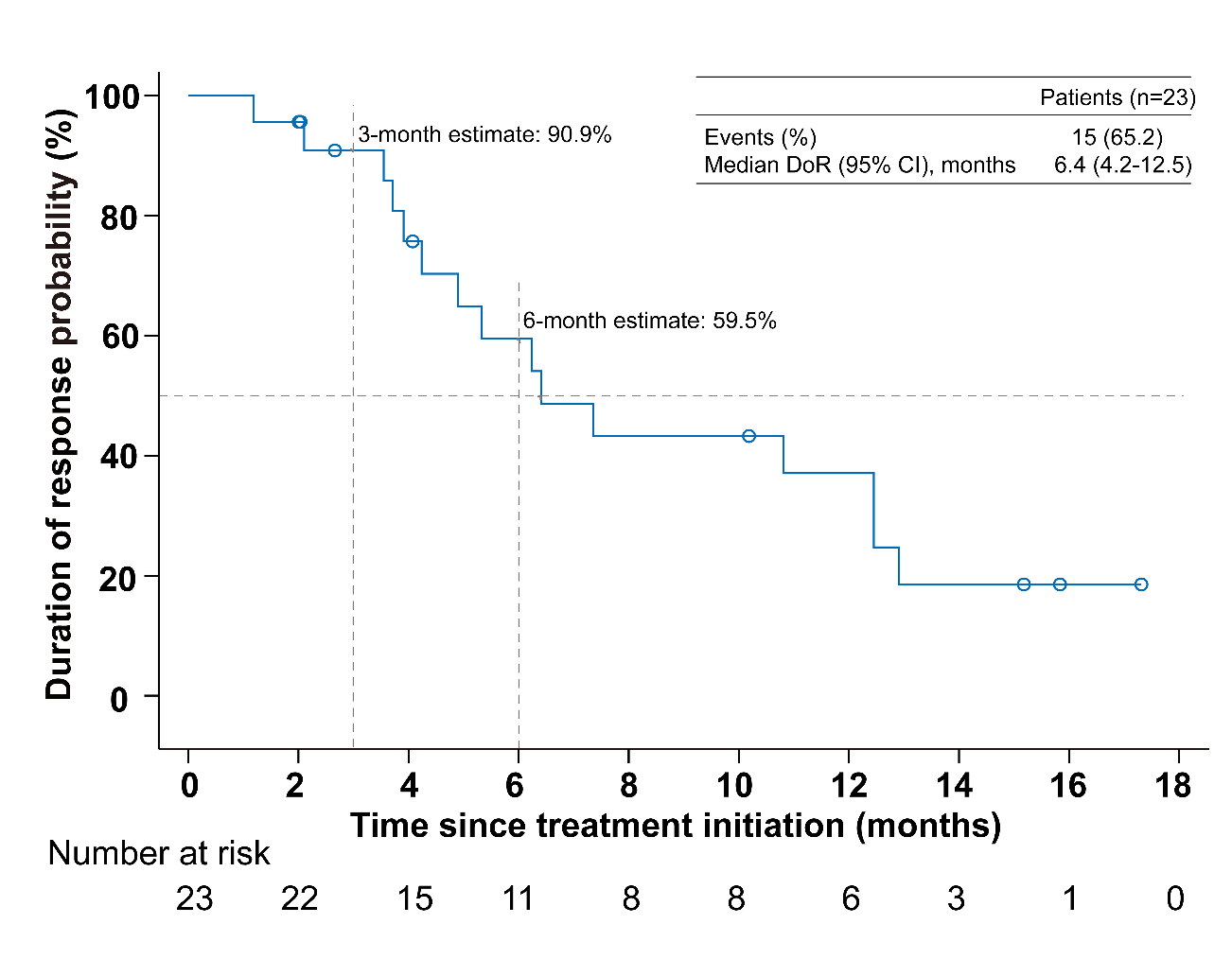
*

## Figure S1. Kaplan-Meier analyses of duration of response as assessed by investigators

CI, confidence interval; DoR, duration of response. Of the 23 patients achieving complete or partial response, 2 had unconfirmed partial response.





## Figure S2. Investigator-assessed PFS and OS based on the expression levels of TIGIT and PD-L1

Kaplan-Meier curves of (a) PFS and (b) OS based on TIGIT expression; (c) PFS and (d) OS based on PD-L1 expression; (e) PFS and (f) OS based on combined TIGIT and PD-L1 expression. PFS, progression-free survival; OS, overall survival; PD-L1, programmed death ligand-1; TIGIT, T-cell immunoreceptor with immunoglobulin and immunoreceptor tyrosine-based inhibition motif domain; NR, not reached; CI, confidence interval.

**Unpublished data by Pei YZ et al. (Zhongshan Hospital, Fudan University)**

Our previous retrospective study aimed to investigate the expression profiles of both PD-L1 and TIGIT in tumour tissues from 297 patients with ICC and analyze the prognostic implications of these two molecules. Based on different levels of PD-L1/TIGIT expression, our results showed that patients with high PD-L1/high TIGIT was associated with a poor overall survival among the four subgroups (Figure S3).


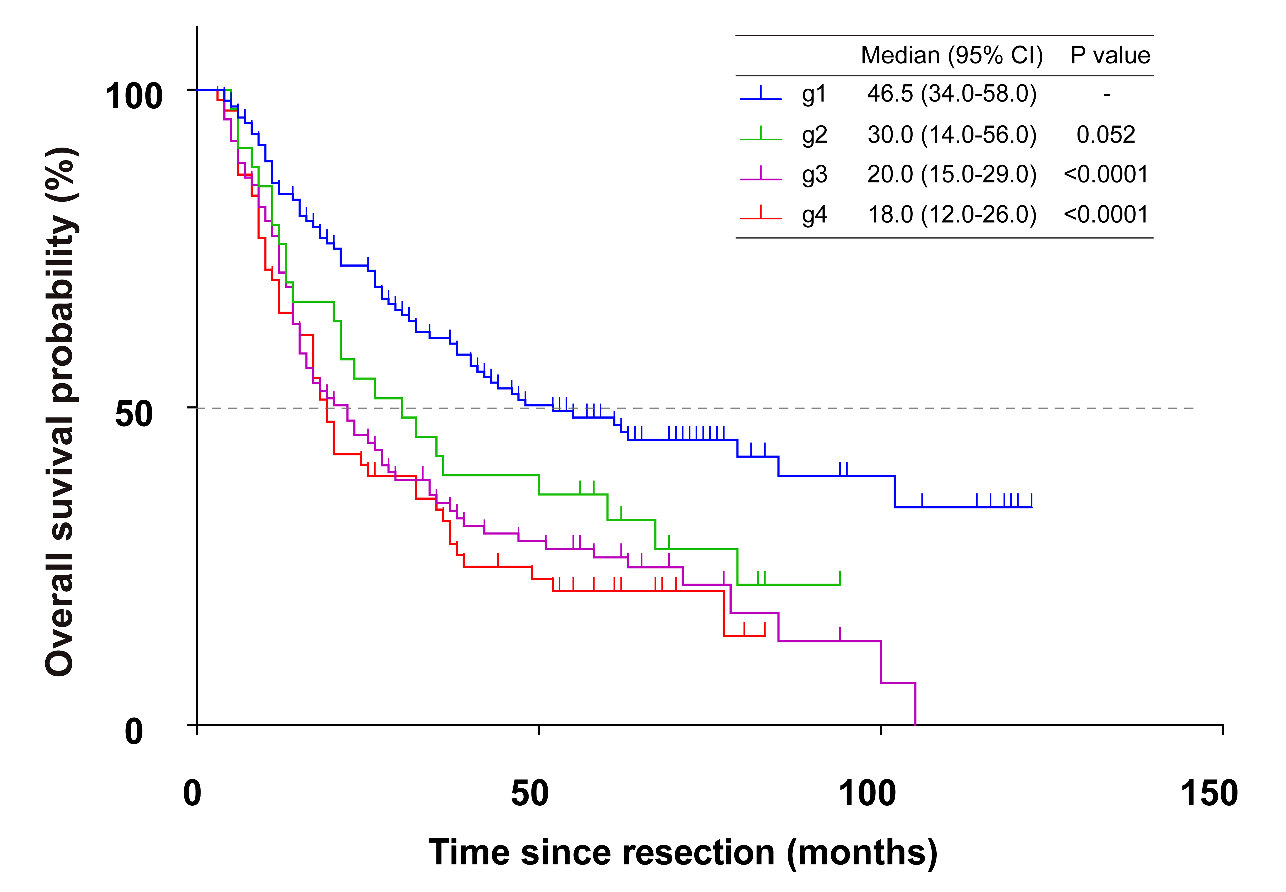


## Figure S3. Overall survival based on different levels of PD-L1/TIGIT expression

TILs, tumour-infiltrating lymphocytes; TIGIT, T-cell immunoreceptor with immunoglobulin and immunoreceptor tyrosine-based inhibition motif domain; PD-L1, programmed death ligand-1; g1, Tumour^low PD-L1^/TILs^low TIGIT^ (n=116); g2, Tumour^low PD-L1^/TILs^high TIGIT^ (n=87); g3, Tumour^high PD-L1^/TILs^low TIGIT^ (n=33); g4, Tumour^high PD-L1^/TILs^high TIGIT^ (n=61).
