# Supplementary material for: First-line tislelizumab and ociperlimab combined with gemcitabine and cisplatin in advanced biliary tract cancer (ZSAB-TOP): a multicenter, single-arm, phase 2 study
Source: Signal Transduct Target Ther. 2025 Aug 21;10:260. doi: 10.1038/s41392-025-02356-y (PMC12368246; doi:10.1038/s41392-025-02356-y)
Supplement: Supplementary file 4 — Study protocol [file 41392_2025_2356_MOESM4_ESM.docx]

CLINICAL STUDY PROTOCOL

| **PROTOCOL TITLE:** | An Open-label, Multicenter, Single-arm Exploratory Study Investigating the Efficacy and Safety of GP Regimen (Gemcitabine + Cisplatin) in Combination With Tislelizumab and Ociperlimab as First-line Treatment for Unresectable Advanced Biliary Tract Carcinoma (BTC) |
| --- | --- |
| **PROTOCOL IDENTIFIER:** | BGB-A1217-2001-IIT |
| **INVESTIGATIONAL PRODUCTS:** | Tislelizumab, Ociperlimab, Gemcitabine, Cisplatin |
| **INDICATION:** | First-line Treatment for BTC |
| **SPONSOR:** | Zhongshan Hospital Affiliated to Fudan University  No. 1609 Xietu Road, Xudhui District, Shanghai |

**CONFIDENTIALITY STATEMENT**

The information contained in this document (especially unpublished data) is confidential which is provided for review by you, your staff, and applicable ethics committee only. This document may not be disclosed to any unauthorized individuals without the prior written approval of sponsor unless for the purpose of providing necessary information to obtain informed consents for candidate patients.

PROTOCOL APPROVAL SHEET

Protocol Title: An Open-label, Multicenter, Single-arm Exploratory Study Investigating the Efficacy and Safety of GP Regimen (Gemcitabine + Cisplatin) in Combination With Tislelizumab and Ociperlimab as First-line Treatment for Unresectable Advanced Biliary Tract Carcinoma (BTC)

Protocol Identifier: BGB-A1217-2001-IIT

Sponsor: Zhongshan Hospital Affiliated to Fudan University

Sponsor Authorized Signatory of Protocol/Protocol Amendment

Jia Fan, Academician

Job Title: President of Zhongshan Hospital Affiliated to Fudan University

Email: fan.jia@zs-hospital.sh.cn

**SIGNATURE: DATE:**

SITE PRINCIPAL INVESTIGATOR SIGNATURE PAGE

Protocol Title: An Open-label, Multicenter, Single-arm Exploratory Study Investigating the Efficacy and Safety of GP Regimen (Gemcitabine + Cisplatin) in Combination With Tislelizumab and Ociperlimab as First-line Treatment for Unresectable Advanced Biliary Tract Cancer (BTC)

Protocol Identifier: BGB-A1217-2001-IIT

**I confirm that i have read this protocol, i understand it, and i will work according to this protocol. I will also work consistently with the ethical principles that have their origin in the declaration of helsinki and that are consistent with good clinical practices and the applicable laws and regulations. Acceptance of this document constitutes my agreement that no unpublished information contained herein will be published or disclosed without prior written approval from sponsor.**

**I have read this protocol in its entirety and agree to conduct the study accordingly:**

**Investigator’s name: _______________________________**

**Investigator title:_______________________________**

**Name/Address of site:_______________________________**

**Signature of investigator:______________Date: _____________**

Synopsis

| **Study Title** | An Open-label, Multicenter, Single-arm Exploratory Study Investigating the Efficacy and Safety of GP Regimen (Gemcitabine + Cisplatin) in Combination With Tislelizumab and Ociperlimab as First-line Treatment for Unresectable Advanced Biliary Tract Carcinoma (BTC) |
| --- | --- |
| **Protocol Identifier** | BGB-A1217-2001-IIT |
| **Sponsor Name** | Zhongshan Hospital Affiliated to Fudan University |
| **Principal Investigator** | Jia Fan, Academician |
| **Number of Planned Study Patients** | 45 |
| **Study Site(s):** | 3 sites in China |
| **Study Objectives**  **Primary Objective:**   - To assess efficacy of GP in combination with tislelizumab and ociperlimab as first-line treatment for unresectable advanced BTC by objective response rate (ORR) as assessed by the investigator per Response Evaluation Criteria in Solid Tumors (RECIST) version1.1.   **Secondary Objectives:**   - To assess safety and tolerability of GP in combination with tislelizumab and ociperlimab as first-line treatment for unresectable advanced BTC. - To assess efficacy of GP in combination with tislelizumab and ociperlimab as first-line treatment for unresectable advanced BTC by disease control rate (DCR) as assessed by the investigator. - To assess efficacy of GP in combination with tislelizumab and ociperlimab as first-line treatment for unresectable advanced BTC by duration of response (DoR) as assessed by the investigator. - To assess efficacy of GP in combination with tislelizumab and ociperlimab as first-line treatment for unresectable advanced BTC by progression-free survival (PFS) and PFS rates at 6 months and at 12 months as assessed by the investigator. - To assess efficacy of GP in combination with tislelizumab and ociperlimab as first-line treatment for unresectable advanced BTC by overall survival (OS) and OS rates at 6 months and at 12 months as assessed by the investigator.   **Exploratory Objective:**   - To explore potential biomarkers that may correlate with clinical efficacy/prognosis of GP in combination with tislelizumab and ociperlimab as first-line treatment for unresectable advanced BTC. | |
| **Study Endpoints**  **Primary Endpoint:**   - ORR assessed by the investigator per RECIST v1.1: the proportion of patients who have achieved complete response (CR) or partial response (PR).   **Secondary Endpoints:**   - DCR assessed by the investigator per RECIST v1.1: the proportion of patients whose DCR is CR, PR, and stable disease (SD). - DoR assessed by the investigator per RECIST v1.1: the time from the date that the response criteria (CR or PR) are first met until the confirmation of disease progression (PD) or death, whichever occurs first - PFS assessed by the investigator: the time from the beginning of study treatment to the date of disease progression as assessed per RECIST v1.1 or death from any cause, whichever occurs first - PFS rates assessed by the investigator: the proportion of patients remain alive and progression-free as assessed per RECIST v1.1 at the 6 and 12 months from the beginning of study treatment. - OS assessed by the investigator: the time from the beginning of study treatment until the date of death. - OS rates assessed by the investigator: the proportion of patients remain alive at the 6 and 12 months from the beginning of study treatment. - Incidence, nature, and severity of adverse events, serious adverse events per the National Cancer Institute-Common Terminology Criteria for Adverse Events (NCI‑CTCAE) Version 5.0: all these parameters will be determined based on NCI-CTCAE v5.0. Safety and tolerability will be assessed in combination with clinical laboratory abnormalities, relevant physical examinations, electrocardiograms (ECGs) and vital signs.   **Exploratory Endpoint:**   - Correlation between expression levels of potential biomarkers programmed cell death ligand-1 (PD-L1) and T-cell immunoglobulin and ITIM domain (TIGIT) with disease status, response to/prognosis of GP in combination with tislelizumab and ociperlimab as first-line treatment for unresectable advanced BTC. | |
| **Study Population**  Patients diagnosed with unresectable advanced BTC (including intrahepatic cholangiocarcinoma [ICC], extrahepatic cholangiocarcinoma [ECC] and gallbladder cancer [GBC]) and confirmed histologically or cytologically. | |
| **Main Inclusion Criteria**  Patients are eligible for inclusion in the study if they meet the following criteria:  1. Male or female, aged 18 to 75 years old  2. Patients must be able to understand and willing to sign the written informed consent form (ICF) and must sign the ICF before enrollment.  3. Patients with histologically or cytologically confirmed unresectable advanced BTC (including ICC, ECC, and GBC), and agree to submit archival tumor tissue samples or fresh tumor biopsies for biomarker testing.  4. Have least 1 measurable lesion as defined by RECIST v1.1.  5. Have not received any prior systemic therapy for biliary tract tumors, including chemotherapy and immunotherapy.  6. Eastern Cooperative Oncology Group (ECOG) Performance Status (PS) score 0 to 1.  7. Child-Pugh Class A classification for liver function.  8. Patients must have adequate bone marrow, liver and kidney function as indicated by the following laboratory assessment criteria (obtained ≤ 7 days before the first dose of study treatment):  Hematology:  Absolute neutrophil count (ANC) ≥ 1.5 x 10^9^/L, platelets ≥ 100 x 10^9^/L, and hemoglobin ≥ 90 g/L  Hepatic function:  AST and ALT ≤ 3 x upper limit of normal value (ULN); total bilirubin ≤ 1.5 x ULN  Patients with obstructive jaundice can be considered for enrollment if their hepatic function meet the inclusion criteria following the treatment with percutaneous transhepatic cholangial drainage (PTCD) or endoscopic retrograde cholangiopancreatography (ERCP)  Renal function:  Serum creatinine clearance (CrCl) ≥ 45 mL/min  Coagulation:  International normalized ratio (INR) ≤ 1.5  9. Patients with HBV or HCV infection must meet the following criteria:  For patients with inactive/asymptomatic HBV carrier, chronic, or active HBV:  Must have HBV DNA < 2000 copies/mL at Screening.  Note: Patients with detectable hepatitis B surface antigen (HBsAg) or detectable HBV DNA should be managed per treatment guidelines. Patients receiving antiviral treatments at Screening should have HBV DNA < 2000 copies/mL and will continue the antiviral treatment during the study.  For patients with HCV infection:  Detectable HCV RNA level is indicative of infection and patients with detectable HCV RNA are not eligible to participate in this study.  10. Females of childbearing potential (i.e., have the physiological ability to become pregnant) must be willing to use a highly effective method of birth control for the duration of the study and for 120 days after the last dose of study treatment, and have had a negative urine or serum pregnancy test within 7 days of the first dose of study treatment.  11. Non-sterile males must be willing to use a highly effective method of birth control for the duration of the study and for 120 days after the last dose of study treatment.  12. Life expectancy ≥ 3 months.  **Main Exclusion Criteria**  Patients are not eligible to participate in this study if they meet any of the following exclusion criteria:  1. Diagnosis of ampullary cancer, mixed hepatocellular and cholangiocellular carcinoma;  2. History of severe hypersensitivity reactions to other monoclonal antibodies;  3. Allergy to tislelizumab, ociperlimab (anti-TIGIT inhibitor) or any of its excipients; allergy to cisplatin and any of its excipients; allergy to gemcitabine and any excipients;  4. Pericardial effusion, uncontrollable pleural effusion, or clinically significant ascites within 7 days before the first dose of study treatment, defined as meeting the following criteria: (a) ascites can be detected during physical examination at Screening, and (b) ascites that requires abdominal tapping for drainage at Screening;  5. Clinical evidence of portal hypertension with esophageal or gastric varices within 6 months before the first dose of study treatment;  6. Bleeding or thrombotic disorders or use of anticoagulants, such as warfarin or similar agents, requiring therapeutic international normalized ratio (INR) monitoring within 6 months before the first dose of study treatment;  7. Prior malignancies except for the BTC under investigation in this study and any locally recurring cancer that has been treated curatively (e.g, resected basal or squamous cell skin cancer, superficial bladder cancer, carcinoma *in situ* of the cervix or breast);  8. Any known central nervous system metastasis and/or leptomeningeal disease before the first dose of study treatment;  9. Active immunodeficiency or autoimmune disease(s) at Screening and/or history of immunodeficiency or autoimmune disease(s) that may relapse at Screening;  Note: patients with the following conditions will not be excluded:  • Type 1 diabetes mellitus that is clinically manageable  • Hypothyroidism that is controlled by hormonal replacement therapy  • Controlled celiac disease (≤ 3 times/day)  • Skin diseases not requiring systemic treatment (e.g, vitiligo, psoriasis, or alopecia)  • Any other disease that is not expected to recur in the absence of external triggering factors  10. Any condition that requires systemic treatment with either corticosteroids (dose > 10 mg daily of prednisone or equivalent) or other immunosuppressive agents ≤ 14 days before the first dose of study treatment;  Note: Patients who are currently or had previously been on any of the following steroid regimens will not be excluded:  • Adrenal replacement steroid (dose ≤ 10 mg daily of prednisone or equivalent) are permitted in the absence of active autoimmune disease  • Topical, ocular, intra-articular, intranasal, or inhalational corticosteroid with minimal systemic absorption  • Short course (≤ 7 days) of corticosteroid prescribed prophylactically (e.g, for contrast dye allergy) or for the treatment of a non-autoimmune condition (eg, delayed-type hypersensitivity reaction caused by contact allergen)  11. With history of interstitial lung disease or non-infectious pneumonitis;  12. Severe chronic or active infection (including tuberculosis, excluding viral hepatitis) requiring systemic antibacterial, antifungal, or antiviral therapy before the first dose of study treatment;  13. Screening ECG shows QT corrected (QTc) interval (corrected by Fridericia’s method) > 450 msec  Note: An ECG will be repeated to confirm any reported QTc interval > 450 msec from the initial testing.  15. Any of the following cardiovascular risk factors:   - Cardiac chest pain, defined as moderate pain that limits instrumental activities of daily living (ADL), within 28 days before the first dose of study treatment; - Symptomatic pulmonary embolism within 28 days before the first dose of study treatment; - Any history of acute myocardial infarction within 6 months before the first dose of study treatment; - Any history of heart failure meeting New York Heart Association Classification III or IV within 6 months before the first dose of study treatment; - Any event of ventricular arrhythmia ≥ Grade 2 in severity within 6 months before the first dose of study treatment; - Cerebral vascular accident (CVA) or transient ischemic attack (TIA) within 6 months before the first dose of study treatment;   15. Received organ transplantation or hematopoietic stem cell transplantation (HSCT) or any major surgical procedure within 28 days before the first dose of study treatment;  16. Known mental disorders or substance abuses that may compromise the study compliance;  17. Was administered a live vaccine within 28 days before the first dose of study treatment; Note: Seasonal vaccines for influenza are generally inactivated vaccines and are allowed.  18. Known history of human immunodeficiency virus (HIV) infection or syphilis infection;  19. Currently participating in other studies and receiving treatments, or participating in other clinical trials of drug or medical device within 4 weeks after the first dose of study treatment;  20. Pregnant or lactating women, become pregnant or lactating from the Screening visit to 120 days after the last dose of the study treatment, or prepare for pregnancy or give birth to a child within the planned study duration;  21. Poor study compliance as determined by the investigator, or other conditions that render the patient ineligible for the study;  22. Medical contraindications that preclude the use of contrast-enhanced imaging (CT or MRI). | |
| **Investigational Product, Dose, and Mode of Administration:**   - Tislelizumab, at the dose of 200 mg, is diluted in 100 mL of 0.9% normal saline (NS), and will be administered on the first day of each cycle (D1) and repeated dosing once every three weeks (Q3W). - Ociperlimab, at the dose of 900 mg, is diluted in 100 mL of 0.9% NS, and will be administered on the first day of each cycle (D1) and repeated dosing once every three weeks (Q3W).   Treatment with tislelizumab and ociperlimab will continue until disease progression, unacceptable toxicity, death, withdrawal of consent, or other conditions that make the patient unsuitable for the study, as determined by the investigator   - GP: Gemcitabine, at the dose of 1000 mg/m^2^, is diluted in 100 mL of 0.9% NS, and will be infused over 30 minutes; Cisplatin, at the dose of 25 mg/m^2^, is diluted in 100 mL of 0.9% NS, and will be infused over 2 hours; GP regimen will be administered on the first and eighth day of each cycle (D1/D8) and repeated every three weeks (Q3W) until intolerance, disease progression or completion of 8 cycles of chemotherapy   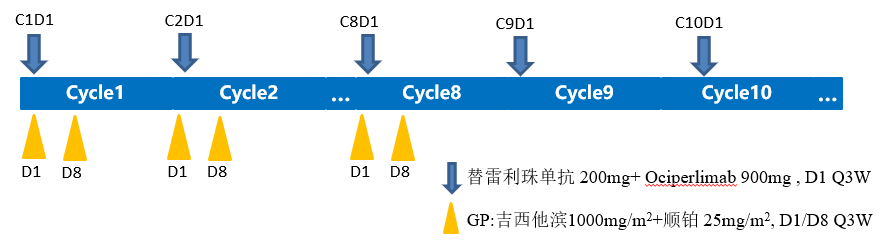  GP: Gemcitabine 1000 mg/m^2^ + Cisplain 25 mg/m^2^, D1/D8 Q3W  Tislelizumab 200 mg + Ociperlimab 900 mg, D1 Q3W  **Tislelizumab Plus Ociperlimab**  Patients will receive tislelizumab at the dose of 200 mg intravenously followed by the administration of ociperlimab at the dose of 900 mg on Day 1 of each 21-day cycle (i.e, once every 3 weeks). The investigational products must be administered via intravenous infusion. Specific instructions for product preparation and administration are provided in the Pharmacy Manual.  The initial infusions (Day 1 of Cycle 1 and Cycle 2) of tislelizumab and ociperlimab will be delivered over 60 minutes; if this is well tolerated, then all the subsequent infusions will be administered over 30 minutes, which is the shortest time period permissible for infusion. Tislelizumab and ociperlimab must not be concurrently administered with any other drug.  As a routine precaution, after the infusion of study treatment is complete on Day 1 of Cycle 1 and Cycle 2, patients must be monitored for ≥ 120 minutes afterward in an area with resuscitation equipment and emergency agents. From Cycle 3 onward, a ≥ 60-minute monitoring period is required in an area with resuscitation equipment and emergency agents.  Refer to the Pharmacy Manual for detailed instructions on drug preparation, storage, and administration.  **GP: Gemcitabine Plus Cisplatin**  The gemcitabine 1000 mg/m^2^ + cisplatin 25 mg/m^2^ regimen will be administered on the first and eighth day of each cycle for up to 8 cycles, with chemotherapy cycles and dosage being adjusted based on tolerability.  Patients who are intolerant to chemotherapy or have achieved stable disease or objective response after completing 8 cycles of chemotherapy will continue the treatment of tislelizumab 200 mg plus ociperlimab 900 mg until disease progression, unacceptable toxicity, death, withdrawal of consent, or other conditions that make the patient unsuitable for the study, as determined by the investigator | |
| **Study Design**  This is an open-label, multicenter, single-arm phase 2 study designed to evaluate the efficacy and safety of GP in combination with tislelizumab and ociperlimab as first-line treatment for unresectable advanced BTC. The study aims to explore the correlation between expression levels of potential biomarkers PD-L1 and TIGIT with disease status, treatment response/prognosis.  The study schema is presented below:  Tumor tissue biomarker testing: PD-L1, TIGIT  Pathologically diagnosed with unresectable advanced BTC (ICC, ECC or GBC)  Have not received any prior systemic therapy for biliary tract tumors;  ECOG PS 0-1  Child Pugh A  Treatment until PD  or Death  or Unacceptable toxicity  or Withdrawl of consent  or Unfit for study treatment as determined by the investigator  Whichever occurs first  Efficacy/Safety/Survival  Follow-ups  GP (Gemcitabine + Cisplatin)+ Tislelizumab + Ociperlimab  Follow-up^c^  Treatment^b,c^  Screening^a^  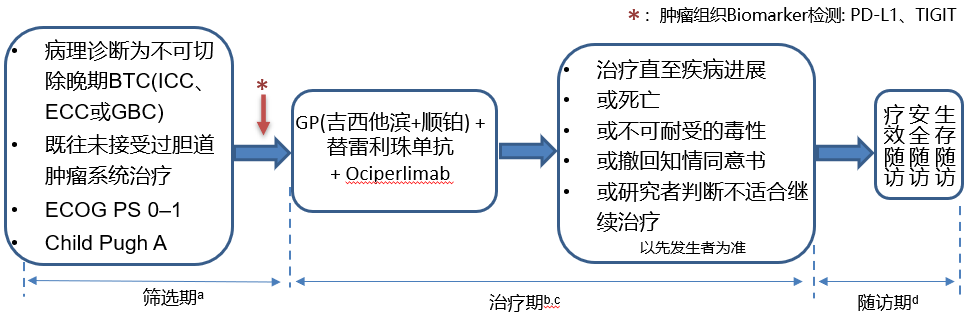   1. Archival tumor tissue samples or fresh biopsies must be available for biomarker testings 2. Tislelizumab and ociperlimab will be administered intravenously on Day 1 of each 21-day cycle (once every 3 weeks). Tislelizumab (200 mg) and ociperlimab (900 mg) will be administered at flat doses. 3. Gemcitabine 1000 mg/m^2^ + cisplatin 25 mg/m^2^ regimen will be administered on the first and eighth day of each cycle for up to 8 cycles, with chemotherapy cycles and dosage being adjusted based on tolerability. Patients who are intolerant to chemotherapy or have achieved stable disease or objective response after completing 8 cycles of chemotherapy will continue the treatment of tislelizumab 200 mg plus ociperlimab 900 mg until disease progression, unacceptable toxicity, death, withdrawal of consent, or other conditions that make the patient unsuitable for the study, as determined by the investigator. 4. Survival follow-up should continue until initiation of subsequent anticancer therapy, the patient withdraws consent, dies, is lost to follow-up or the study terminates, whichever occurs first.   **Study Follow-up and Assessments:**  During the study, hematology and other tests are required before administration of chemotherapy on D1 and D8 of each cycle as well as before administration of tislelizumab and ociperlimab maintenance treatment. Assessments including tumor imaging (enhanced CT or MRI), hematology, serum alpha-fetoprotein (AFP), serum carcinoembryonic antigen (CEA), CA 19-9, CA 125, etc. will be performed every 9 weeks (± 7 days).  Tumor response will be assessed by the investigator per RECIST v1.1. Tumor imaging (enhanced CT or MRI) must be performed within 28 days before the first dose of study treatment. During the study, tumor assessments will be performed every 9 weeks (± 7 days). After documentation of initial response (CR or PR), confirmation of tumor response should occur ≥ 4 weeks but ≤ 6 weeks after the initial response. Tumor assessment should continue until disease progression is determined by the investigator. If a patient discontinues study treatment due to any reason other than disease progression (such as toxicity), tumor assessments will continue as scheduled until disease progression, death, withdrawal of consent, loss to follow-up, or until the termination of study, whichever occurs first.  Patients will be evaluated for any adverse events (AEs) and serious adverse events (SAEs) (severity grades, per NCI-CTCAE v5.0). After the ICF has been signed but before the administration of study treatment(s), only SAEs should be reported. After initiation of the study treatment(s) administration, all AEs and SAEs, regardless of relationship to the study treatment(s), will be reported until either 30 days after the last dose of study treatment, or until the initiation of a new anticancer therapy, whichever occurs first. Immune-related AEs (serious or non-serious) will be reported until 90 days after the last dose of study treatment, regardless of whether the patient starts a new anticancer therapy. All SAEs considered related to the study treatment(s) that are brought to the attention of the investigator should be reported regardless of time since the last dose of study treatment.  **End of study:**  The study will continue until the last patient's death, loss to follow-up, or withdrawal from the study and is expected to conclude approximately 2 years after the enrollment of the last patient. Safety and efficacy analyses will be conducted upon the observation of a predefined number of events.  **Biomarker(s) Testing:**  Enrolled patients are required to provide tumor tissues (formalin-fixed paraffin-embedded tissue or fresh unstained slides) for the detection of PD-L1 and TIGIT expression in a central laboratory to explore the correlation with disease status, treatment response/prognosis.  Note:Written informed consent is required before obtaining fresh tumor biopsies. Tumor tissues need to originate from core or punch biopsy. Tumor tissues from fine-needle aspiration are not acceptable; In case of submitting unstained cut slides, freshly cut slides should be submitted to the testing laboratory within 14 days from when the slides are cut. | |
| **Statistical Methods:**  **Analysis Set:**   - The Safety Analysis Set includes all patients who received at least1 dose of any study treatment (any component for the combination therapy). The Safety Analysis Set will be used for all safety analyses. - The Efficacy Analysis Set includes all patients who are in the Safety Analysis Set, had measurable disease at baseline per RECIST v1.1 and have at least one evaluable post-baseline tumor assessment unless discontinued treatment due to clinical progression or death prior to the first post-treatment tumor assessment. The Efficacy Analysis Set will be used for all efficacy analyses.   **Primary Efficacy Endpoint Analysis:**  The primary endpoint ORR will be based upon the investigator’s tumor assessments per RECIST v1.1 from the Efficacy Analysis Set. The targeted ORR for the combination regimen (GP in combination with tislelizumab and ociperlimab) is 45%, whereas the historical control ORR for gemcitabine plus cisplatin in a similar population was 25%. The null and alternative hypotheses are thus set as follows:  H0: ORR = 25%  Ha: ORR > 25%  A binomial exact test will be performed for hypothesis testing. If the obtained one-sided p-value is ≤ 0.05, it is to be concluded that the investigational products statistically significantly increases ORR compared with historical control, and thus demonstrating superiority of the investigational products.  **Secondary Efficacy Endpoints Analyses:**  Time-to-event variables, including PFS, OS, and DoR, will be estimated using the Kaplan-Meier (KM) method, with Kaplan-Meier curves generated and plotted over time. The medians of PFS, OS and DoR along with their 95% confidence intervals (CIs) (constructed using Brookmeyer and Crowley method [Brookmeyer and Crowley 1982]) (if estimable) will be estimated based on the investigator’s assessment per RECIST v1.1. Meanwhile, landmark rates at selected time points (6 months and 12 months) for PFS and OS will be estimated using the Kaplan-Meier method with the corresponding 95% CIs constructed using Greenwood’s formula (Greenwood 1926). The binomial exact 95% CIs will be used for assessing DCR within the Efficacy Analysis Set.  **Exploratory Endpoint Analysis:**  The correlation between expression levels of potential biomarkers programmed cell death ligand-1 (PD-L1) and T-cell immunoglobulin and ITIM domain (TIGIT) with disease status, response to/prognosis of GP in combination with tislelizumab and ociperlimab as first-line treatment for unresectable advanced BTC will be analyzed using multivariate regression analysis in the Efficacy Analysis Set.  **Safety Analysis:**  AEs will be graded for severity according to the NCI‑CTCAE v5.0. Laboratory values (e.g., hematology, clinical chemistry and urinalysis), vital signs, ECGs, as well as physical examinations will be used for determining safety profiles. Descriptive statistics (e.g., n, mean, standard deviation, median, minimum, maximum for continuous variables; n [%] for categorical variables) will be used to analyze all safety data in the Safety Analysis Set.  **Sample Size Calculation:**  The study plans to enroll 45 patients.  Based on the the exact binomial distribution method adopted for this single-arm trial, the sample size of 36 patients will provide 80% statistical power to demonstrate statistically significant difference between the ORR of combination regimen (assumed to be 45%, as assessed per RECIST v1.1) and the historical control ORR of 25% at a one-sided alpha level of 0.05. Assuming a 20% drop-out rate, a total of 45 patients will be enrolled. | |

TABLE OF CONTENTS

TABLE OF CONTENTS

[CLINICAL STUDY PROTOCOL 1](#_Toc178530145)

[PROTOCOL APPROVAL SHEET 2](#_Toc178530146)

[SITE PRINCIPAL INVESTIGATOR SIGNATURE PAGE 3](#_Toc178530147)

[Synopsis 4](#_Toc178530148)

[TABLE OF CONTENTS 14](#_Toc178530149)

[Definition of Abbreviations and Terms 21](#_Toc178530150)

[1. 1 Study Background 24](#_Toc178530151)

[1.1. Overview of Biliary Tract Carcinoma 24](#_Toc178530152)

[1.2. Current Treatments for BTC 24](#_Toc178530153)

[1.3. Application of Immunecheckpoint Inhibitors (ICIs) in the Treatment of BTC 26](#_Toc178530154)

[1.3.1. Immune Microenvironment in Biliary Tract Carcinoma 26](#_Toc178530155)

[1.3.2. Clinical Studies of Immune Checkpoint Inhibitor Monotherapy for Advanced BTC 26](#_Toc178530156)

[1.3.3. ICIs-containing Combination Therapy for BTC 27](#_Toc178530157)

[2. Background Information on Tislelizumab 29](#_Toc178530158)

[2.1. Pharmacology 29](#_Toc178530159)

[2.2. Toxicology 30](#_Toc178530160)

[2.3. Clinical Pharmacology 30](#_Toc178530161)

[2.4. Prior Clinical Experience With Tislelizumab 31](#_Toc178530162)

[2.4.1. Study BGB-A317_Study_001 (Monotherapy) 31](#_Toc178530163)

[2.4.2. Study BGB-A317_Study_102 (Monotherapy) 32](#_Toc178530164)

[2.4.3. Study BGB-A317_Study_203 (Monotherapy) 32](#_Toc178530165)

[2.4.4. Study BGB-A317_Study_204 (Monotherapy) 33](#_Toc178530166)

[2.4.5. Study BGB-A317_Study_207 (Monotherapy) 33](#_Toc178530167)

[2.4.6. Study BGB-A317_Study_208 (Monotherapy) 33](#_Toc178530168)

[2.4.7. Study BGB-A317_Study_209 (Monotherapy) 34](#_Toc178530169)

[2.5. Pooled Safety Assessment of Monotherapy Studies 34](#_Toc178530170)

[2.5.1. DLTs Occurred in Dose-Escalation and Determination of MTDs 35](#_Toc178530171)

[2.5.2. Treatment-Emergent Adverse Events As Assessed Related to Monotherapy 36](#_Toc178530172)

[2.5.3. Special Categories of Immune Related Adverse Events Reported in Monotherapy Studies 38](#_Toc178530173)

[2.5.4. Fatal Adverse Events Reported in Monotherapy Studies 40](#_Toc178530174)

[2.6. Pooled Efficacy Assessment of Monotherapy Studies 40](#_Toc178530175)

[2.6.1. Study BGB-A317-001 40](#_Toc178530176)

[2.6.2. Study BGB-A317-102 40](#_Toc178530177)

[2.7. Clinical Safety of Tislelizumab 41](#_Toc178530178)

[2.8. Rationale for the Selection of the Tislelizumab Dose 42](#_Toc178530179)

[3. Background Information on Ociperlimab As a TIGIT Inhibitor 42](#_Toc178530180)

[3.1. Nonclinical Summary 42](#_Toc178530181)

[3.1.1. Pharmacology 42](#_Toc178530182)

[3.1.2. Toxicology 43](#_Toc178530183)

[3.2. Prior Clinical Experience With Ociperlimab 44](#_Toc178530184)

[3.3. Clinical Pharmacology of Ociperlimab 47](#_Toc178530185)

[4. Study Rationale 47](#_Toc178530186)

[4.1. Rationale forSynergistic Effect of Ociperlimab plus Tislelizumab 48](#_Toc178530187)

[4.2. Rationale for Combination of Gemcitabine/Cisplatin (GP) with Immunotherapy 48](#_Toc178530188)

[4.3. Rationale for Biomarkers 49](#_Toc178530189)

[5. Study Objectives and Endpoints 49](#_Toc178530190)

[5.1. Study Objectives 49](#_Toc178530191)

[5.1.1. Primary Objective 49](#_Toc178530192)

[5.1.2. Secondary Objectives 50](#_Toc178530193)

[5.1.3. Exploratory Objective 50](#_Toc178530194)

[5.2. Study Endpoints 50](#_Toc178530195)

[5.2.1. Primary Endpoint 50](#_Toc178530196)

[5.2.2. Secondary Endpoints 50](#_Toc178530197)

[5.2.3. Exploratory Endpoint 51](#_Toc178530198)

[6. Study Design 51](#_Toc178530199)

[6.1. Summary of Study Design 51](#_Toc178530200)

[6.2. Screening Period 52](#_Toc178530201)

[6.3. Treatment Period 52](#_Toc178530202)

[6.4. End-of-Treatment Visit and Safety Follow-up Telephone Visit 53](#_Toc178530203)

[6.5. Survival Follow-up Visit 54](#_Toc178530204)

[6.6. Discontinuation of Study Treatment/Study Discontinuation by Patients or Investigator 54](#_Toc178530205)

[6.6.1. Discontinuation of Study Treatment 54](#_Toc178530206)

[6.6.2. Patient Discontinuation From Study (End of Study for an Individual Patient) 54](#_Toc178530207)

[6.6.3. Study Termination and Study Site Closure 55](#_Toc178530208)

[7. Study Population 55](#_Toc178530209)

[7.1. Inclusion Criteria 56](#_Toc178530210)

[7.2. Exclusion Criteria 57](#_Toc178530211)

[8. Study Treatment(s) 59](#_Toc178530212)

[8.1. Formulation, Packaging and Handling 59](#_Toc178530213)

[8.1.1. Tislelizumab 59](#_Toc178530214)

[8.1.2. Ociperlimab 59](#_Toc178530215)

[8.1.3. Gemcitabine and Cisplatin 60](#_Toc178530216)

[8.2. Dosage, Administration, and Compliance 60](#_Toc178530217)

[8.2.1. Treatment Administration 60](#_Toc178530218)

[8.2.2. Handling of Overdose 61](#_Toc178530219)

[8.2.3. Investigational Medical Products Accountability 62](#_Toc178530220)

[8.2.4. Dose Modification or Delay 62](#_Toc178530221)

[8.2.5. Disposal and Destruction 66](#_Toc178530222)

[9. Prior and Concomitant Therapies 66](#_Toc178530223)

[9.1. Prior Therapies 66](#_Toc178530224)

[9.2. Concomitant Therapies 66](#_Toc178530225)

[9.3. Excluded (Prohibited or Restricted) Therapies 67](#_Toc178530226)

[10. Study Assessments and Procedures 67](#_Toc178530227)

[10.1. Screening 68](#_Toc178530228)

[10.1.1. Demographic Data and Medical History 68](#_Toc178530229)

[10.1.2. Women of Childbearing Potential and Contraception 68](#_Toc178530230)

[10.1.3. Informed Consent Forms and Screening Log 69](#_Toc178530231)

[10.1.4. Pulmonary Function Test 69](#_Toc178530232)

[10.2. Enrollment 69](#_Toc178530233)

[10.2.1. Confirmation of Eligibility 69](#_Toc178530234)

[10.2.2. Patient Study Number 69](#_Toc178530235)

[10.2.3. Dispensation of Study Treatment 69](#_Toc178530236)

[10.3. Safety Assessments 69](#_Toc178530237)

[10.3.1. Vital Signs 69](#_Toc178530238)

[10.3.2. Physical Examination 70](#_Toc178530239)

[10.3.3. Eastern Cooperative Oncology Group (ECOG) Performance Status 70](#_Toc178530240)

[10.3.4. Laboratory Safety Assessments 71](#_Toc178530241)

[10.3.5. Pulmonary Function Test 72](#_Toc178530242)

[10.3.6. Electrocardiograms 72](#_Toc178530243)

[10.3.7. Adverse Events 72](#_Toc178530244)

[10.4. Biomarker Testing 73](#_Toc178530245)

[10.5. Tumor and Response Evaluation 73](#_Toc178530246)

[10.6. Visit Windows 74](#_Toc178530247)

[10.7. Unscheduled Visits 74](#_Toc178530248)

[10.8. End of Treatment (EOT) Visit 75](#_Toc178530249)

[10.9. Safety follow-up Visit 75](#_Toc178530250)

[10.10. Survival Follow-up Visit 75](#_Toc178530251)

[11. Safety Monitoring and Reporting 76](#_Toc178530252)

[11.1. Risks Associated With Study Treatments 76](#_Toc178530253)

[11.1.1. Risks Associated with Ociperlimab and Tislelizumab 76](#_Toc178530254)

[11.1.2. Risks Associated With Gemcitabine and Cisplatin 76](#_Toc178530255)

[11.2. General Plan to Manage Safety Concerns 76](#_Toc178530256)

[11.2.1. Eligibility Criteria 76](#_Toc178530257)

[11.2.2. Safety Monitoring Plan 77](#_Toc178530258)

[11.3. Adverse Events 77](#_Toc178530259)

[11.3.1. Definitions and Reporting 77](#_Toc178530260)

[11.3.2. Assessment of Severity 78](#_Toc178530261)

[11.3.3. Assessment of Causality 78](#_Toc178530262)

[11.3.4. Follow-up of Adverse Events 79](#_Toc178530263)

[11.3.5. Laboratory Test Abnormalities 80](#_Toc178530264)

[11.4. Definition of a Serious Adverse Event 80](#_Toc178530265)

[11.5. Timing, Frequency, and Method of Capturing Adverse Events and Serious Adverse Events 81](#_Toc178530266)

[11.5.1. Adverse Event Reporting Period 81](#_Toc178530267)

[11.5.2. Reporting Serious Adverse Events 81](#_Toc178530268)

[11.5.3. Diagnosis Versus Recording Signs or Symptoms 82](#_Toc178530269)

[11.5.4. Adverse Events Occurring Secondary to Other Events 83](#_Toc178530270)

[11.5.5. Recording Persistent or Recurrent Adverse Events 83](#_Toc178530271)

[11.5.6. Disease Progression 83](#_Toc178530272)

[11.5.7. Recording Deaths 83](#_Toc178530273)

[11.5.8. Recording Pregnancy 84](#_Toc178530274)

[11.5.9. Recording Post-study Adverse Events 84](#_Toc178530275)

[11.5.10. Management of Adverse Events of Special Interest 84](#_Toc178530276)

[11.5.11. Infusion-Related Reactions 84](#_Toc178530277)

[11.5.12. Severe Hypersensitivity Reactions and Flu-Like Symptoms 86](#_Toc178530278)

[11.5.13. Immune-Related Adverse Events 86](#_Toc178530279)

[11.5.14. Abnormal Kidney Function 87](#_Toc178530280)

[12. Statistical Methods and Sample Size Determination 88](#_Toc178530281)

[12.1. Statistical Analysis 88](#_Toc178530282)

[12.1.1. Analysis Sets 88](#_Toc178530283)

[12.1.2. Patients Disposition 89](#_Toc178530284)

[12.1.3. Demographics and Other Baseline Characteristics 89](#_Toc178530285)

[12.1.4. Prior and Concomitant Medications 89](#_Toc178530286)

[12.2. Efficacy Analysis 89](#_Toc178530287)

[12.2.1. Primary Efficacy Analysis 89](#_Toc178530288)

[12.2.2. Secondary Efficacy Analyses 90](#_Toc178530289)

[12.2.3. Exploratory Efficacy Analysis 90](#_Toc178530290)

[12.3. Safety Analysis 90](#_Toc178530291)

[12.3.1. Extent of Exposure 90](#_Toc178530292)

[12.3.2. Adverse Events 91](#_Toc178530293)

[12.3.3. Laboratory Analyses 91](#_Toc178530294)

[12.3.4. Vital Signs 91](#_Toc178530295)

[12.4. Sample Size Consideration 91](#_Toc178530296)

[13. Source Documents and Access to Source Data/Documents 92](#_Toc178530297)

[13.1. Access to Information for Monitoring 92](#_Toc178530298)

[13.2. Access to Information for Audit or Inspection 92](#_Toc178530299)

[14. Quality Assurance and Quality Control 92](#_Toc178530300)

[14.1. Quality Assurance 92](#_Toc178530301)

[14.2. Site Inspection 93](#_Toc178530302)

[14.3. Drug Accountability 93](#_Toc178530303)

[15. Ethics/Protection of Human Patients 93](#_Toc178530304)

[15.1. Ethical Standard 93](#_Toc178530305)

[15.2. Institutional Review Board/Independent Ethics Committee 94](#_Toc178530306)

[15.3. Protocol Modifications 94](#_Toc178530307)

[15.4. Informed Consent 94](#_Toc178530308)

[15.5. Patient and Data Confidentiality 95](#_Toc178530309)

[16. Data Handling and Records Retention 95](#_Toc178530310)

[16.1. Data Collection and Management Responsibilities 95](#_Toc178530311)

[16.1.1. Data Collection 95](#_Toc178530312)

[16.1.2. Data Management/Coding 95](#_Toc178530313)

[16.2. Data Integrity 96](#_Toc178530314)

[16.3. Study Records Retention 96](#_Toc178530315)

[16.4. Protocol Deviations 97](#_Toc178530316)

[16.5. Publication and Data Sharing Policy 97](#_Toc178530317)

[16.6. Study and Study Site Closure 98](#_Toc178530318)

[16.7. Information Disclosure and Inventions 98](#_Toc178530319)

[17. References 99](#_Toc178530320)

[Appendix 1 Study Procedures 102](#_Toc178530321)

[Appendix 2 ECOG Performance Status 108](#_Toc178530322)

[Appendix 3 Child-Pugh Classification Scoring System 109](#_Toc178530323)

[Appendix 4 Preexisting Immune Deficiencies or Autoimmune Diseases 110](#_Toc178530324)

[Appendix 5 Clinical Laboratory Assessments 111](#_Toc178530325)

[Appendix 6 Contraception Guidelines and Definitions of “Women of Childbearing Potential,” “No Childbearing Potential” 112](#_Toc178530326)

[Appendix 7 New York Heart Association Functional Classification 114](#_Toc178530327)

[Appendix 8 Chronic Kidney Disease Epidemiology Collaboration (CKD-EPI) Equation 115](#_Toc178530328)

[Appendix 9 The Response Evaluation Criteria in Solid Tumors Guidelines (RECIST) Version 1.1 116](#_Toc178530329)

[Appendix 10 Immune‑Related Adverse Event Evaluation and Management 124](#_Toc178530330)

Definition of Abbreviations and Terms

| **Abbreviation** | **Definition** |
| --- | --- |
| AASLD | American Association for the Study of Liver Disease |
| ADA | Anti-drug Antibody |
| ADCC | Antibody-dependent Cellular Cytotoxicity |
| ADCP | Antibody-dependent Cellular Phagocytosis |
| AE | Adverse Event |
| AFP | Alpha Fetoprotein |
| ALT | Alanine Aminotransferase |
| AST | Aspartate Aminotransferase |
| BCLC | Barcelona Clinic Liver Cancer Staging |
| BGB‑A317 | Compound Code of Monoclonal Antibody Tislelizumab |
| Ociperlimab | Compound Code of Monoclonal Antibody Ociperlimab |
| BOR | Best Overall Response |
| BTC | Biliary Tract Carcinoma |
| CI | Confidence Interval |
| CL | Clearance |
| C_max_ | Maximum Observed Plasma Concentration |
| CP | Child-Pugh |
| CP-A | Child-Pugh Grade A |
| CP-B | Child-Pugh Grade B |
| CR | Complete Response |
| CRA | Clinical Research Associate (Clinical Monitor) |
| CSR | Clinical Study Report |
| CT | Computed Tomography |
| CTCAE | Common Terminology Criteria for Adverse Events |
| DCR | Disease Control Rate |
| DLT | Dose Limiting Toxicity |
| DNA | Deoxyribonucleic Acid |
| DOR | Duration of Response |
| eCCG | Electronic Case Report Form Completion Guideline |
| ECC | Extrahepatic Cholangiocarcinoma |
| ECG | Electrocardiograms |
| ECOG | Eastern Cooperative Oncology Group |
| eCRF | Electronic Case Report Form |
| EDC | Electronic Data Capture |
| eGFR | Estimated Glomerular Filtration Rate |
| FcγR | Gamma Fc Receptor (e.g., Fcγ-RI, Fcγ-R2I) |
| FDA | US Food and Drug Administration |
| FFPE | Formalin-fixed Paraffin Embedded |
| GBC | Gallbladder Cancer |
| GCP | Good Clinical Practice |
| HBcAb | Hepatitis B Core Antibody |
| HBsAg | Hepatitis B Surface Antigen |
| HBV | Hepatitis B Virus |
| HCC | Hepatocellular Carcinoma |
| HCV | Hepatitis C Virus |
| HR | Hazard Ratio |
| IB | Investigator’s Brochure |
| ICC | Intrahepatic Cholangiocarcinoma |
| ICF | Informed Consent Form |
| ICH | International Council on Harmonisation |
| ICU | Intensive Care Unit |
| IEC | Independent Ethics Committee |
| IFN-α | Interferon-alpha |
| IFN-γ | Interferon-gamma |
| Ig | Immunoglobulin |
| IgA | Immunoglobulin A |
| IgG | Immunoglobulins (e.g, IgG1, IgG2, IgG3, IgG4) |
| INR | International Normalized Ratio |
| IRB | Institutional Review Board |
| ITT | Intent-to-treat |
| IV | Intravenously |
| K_D_ | Dissociation Constant |
| MCH | Mean Corpuscular Hemoglobin |
| MDSC | Myeloid-derived Suppressor Cells |
| MDRD | Modification of Diet in Renal Disease Equation |
| MedDRA | Medical Dictionary for Regulatory Activities |
| MRI | Magnetic Resonance Imaging |
| MSI | Microsatellite Instability |
| MTD | Maximum Tolerated Dose |
| NCI-CTCAE | National Cancer Institute Common Terminology Criteria for Adverse Events |
| NE | Not Evaluable |
| NCCN | National Comprehensive Cancer Network |
| NSAID | Non-steroidal Anti-inflammatory Drug |
| ORR | Objective Response Rate |
| OS | Overall Survival |
| PD | Progressive Disease |
| PD-1 | Programmed Cell Death Protein-1 |
| PD-L1 | Program Cell Death Protein Ligand-1 |
| PD-L2 | Program Cell Death Protein Ligand-2 |
| PFS | Progression‑free Survival |
| PLT | Platelet Count |
| PP | Per-protocol |
| PR | Partial Response |
| PS | Performance Status |
| PT | Prothrombin Time |
| Q3W | Once Every 3 weeks |
| QTc | QT Interval Corrected For Heart Rate |
| RBC | Red Blood Cell |
| RECIST | Response Evaluation Criteria in Solid Tumors |
| RNA | Ribonucleic Acid |
| SAE | Serious Adverse Event |
| SAP | Statistical Analysis Plan |
| SD | Stable Disease |
| SOC | System Organ Class |
| SUSAR | Suspected Unexpected Serious Adverse Reaction |
| TEAE | Treatment Emergent Adverse Event |
| TIGIT | T-cell Immunoglobulin and ITIM Domain |
| T_max_ | Time to Reach Maximum Concentration |
| t_1/2_ | Elimination Half-life |
| TSH | Thyroid Stimulating Hormone |
| TTP | Time to Progression |
| ULN | Upper Limit of Normal Range |

1 Study Background

- 1. Overview of Biliary Tract Carcinoma

Malignant biliary tract carcinoma (BTC) originates from biliary epithelial cells and includes various pathological types such as adenocarcinoma, squamous cell carcinoma, neuroendocrine tumor and other types. Among them, more than 80% are adenocarcinomas. BTC can be divided into intrahepatic cholangiocarcinoma (ICC), gallbladder cancer (GBC) and extrahepatic cholangiocarcinoma (ECC) based on anatomical origins. ECC can be further classified into hilar, middle segment and lower segment cholangiocarcinomas. However, advancements in embryonic development research and genomic analysis have led to some disagreements regarding this classification**^1^**. BTC accounts for less than 1% of all malignancies and 3% of gastrointestinal malignancies**^2^**. The incidence of BTC varies globally, with the highest rates among Native Americans and Southeast Asian populations**^3-4^**. The annual incidence of BTC in China is 7.48 cases per 100,000, accounting for 2.4% of new cancer cases in China**^5^**. GBC is more common in women, whereas ECC is more common in men**^6^**. The incidence of ICC shows an increasing trend, while that of ECC shows a decreasing trend**^7^**.

The etiology and pathogenesis of BTC are not clear, and the known risk factors include primary sclerosing cholangitis, Caroli disease, hepatolithiasis, trematode infection of liver, viral hepatitis, alcoholic hepatitis, abnormal metabolic status (diabetes, obesity, non-alcoholic fatty liver disease), cirrhosis, etc., and many patients have no obvious predisposing factors **^8-10^**.

- 1. Current Treatments for BTC

Currently, surgical resection is the only curative treatment for BTC; however, radical resection is feasible in only 10% of patients, with 5-year survival rates ranging from 8% to 40%**^11^**; patients with unresectable or recurrent metastatic BTC often have a worse prognosis.

Due to insidious onset, high invasiveness and lack of specific symptoms at early stage, patients with BTC are often diagnosed at middle or advanced stages, making surgical less feasible. BTC is highly prone to recurrence or metastasis after surgery. More than 80% of patients die within 1 year of diagnosis, and the 5-year survival rate of patients with advanced disease is only 2% to 5%**^12^**.

At present, chemotherapy is the mainstay treatment for BTC. The first-line treatment includes gemcitabine plus cisplatin, gemcitabine plus tegafur, capecitabine plus oxaliplatin, etc., among which gemcitabine plus cisplatin is the preferred regimen recommended by both China and international guidelines. There is insufficient evidence to support the clinical value of second-line chemotherapy for the treatment of BTC.

A small sample size phase 2 study confirmed gemcitabine monotherapy as the standard of care for advanced BTC**^13^**. In 2010, a randomized controlled phase 3 clinical trial (ABC-02 study) enrolled 410 patients with histologically or cytologically confirmed unresectable advanced BTC**^14^**. Patients were randomized to receive either gemcitabine plus cisplatin (GP) or gemcitabine alone. After a median follow-up of 8.2 months, the disease control rate (DCR) was 81.4% in the combination arm versus 71.8% in the monotherapy arm (p = 0.049); the median PFS was 8.0 months versus 5.0 months (HR, 0.63; 95% CI, 0.51 to 0.77; P < 0.001), respectively; and the primary endpoint median OS was 11.7 months vs 8.1 months, respectively (HR, 0.64; 95% CI, 0.52 to 0.80; P < 0.001). The incidence of adverse events was similar between the two arms. Study data showed that gemcitabine plus cisplatin significantly improved survival compared with gemcitabine alone in patients with advanced BTC. The phase 3 study solidified the status of chemotherapy combination of gemcitabine plus cisplatin as the standard first-line treatment for advanced BTC.

Consistently, the BT22 study in Asian population also confirmed the superiority of GP regimen**^15^**. The study enrolled 83 patients with advanced BTC, and results showed that median OS and PFS were 11.2 months versus 7.7 months in GP arm versus 5.8 months and 3.7 months in gemcitabine monotherapy arm. Based on this, the GP regimen is established as the standard first-line chemotherapy for advanced BTC, with a level 1 recommendation for the treatment of BTC in both China and international guidelines**^16-17^**.

Other studies have shown that alternatives regimens such gemcitabine plus tegafur (S1) and capecitabine plus oxaliplatin would probably be established as first-line treatment for cholangiocarcinoma**^18-19^**. In addition, other chemotherapy regimens have been explored as first-line treatment for BTC, such as nab-paclitaxel plus gemcitabine**^20^**, FOLFIRI (irinotecan + FU + LV)**^21^**, gemcitabine plus cisplatin and S-1 (GCS regimen)**^22^**, as well as nab-paclitaxel plus gemcitabine and cisplatin (GAP regimen)**^23^**.

There is insufficient evidence to support the clinical value of second-line chemotherapy for the treatment of BTC. Current guidelines recommend symptomatic supports such as biliary stenting or percutaneous transhepatic cholangial drainage (PTCD) for jaundice relief and infection in patients who progressed after receiving first-line treatment. A randomized phase 3 study (study ABC-06), investigating second-line chemotherapy in selected patients with advanced BTC, was presented at the 2019 ASCO annual meeting. The data indicated that the mFOLFOX regimen is a second-line treatment option for cholangiocarcinoma**^24^**. Recent advances in genomic analysis and molecular biology research have further elucidated the pathogenesis of BTC. Many novel targeted therapies, such as those targeting the epidermal growth factor receptor (EGFR) family, anti-angiogenic agents, MEK inhibitors, IDH1/2 inhibitors, and fibroblast growth factor receptor (FGFR) inhibitors, have shown promising anti-tumor activity. Multiple targeted therapies are currently under investigation in clinical trials for BTC, such as derazantinib (NCT0323318), ivosidenib (NCT02989857), and zanidatamab (NCT04466891)**^25^**.

- 1. Application of Immunecheckpoint Inhibitors (ICIs) in the Treatment of BTC

Immune checkpoints are molecules that regulate the immune system's response to foreign invaders. Inhibitory signaling pathways are crucial for maintaining self-tolerance and regulating immune responses in peripheral tissues. Under normal conditions, co-stimulatory molecules are balanced with immune checkpoint molecules to minimize the invasion of surrounding normal tissues. Immune checkpoints, such as programmed death protein-1 (PD-1), cytotoxic T-lymphocyte antigen-4 (CTLA-4), and T-cell immunoglobulin and ITIM domain protein (TIGIT), are often manipulated by tumor cells to evade immune surveillance. When activated by their respective ligands, these checkpoints promote apoptosis in peripheral blood T cells. Recent findings have shown that immune checkpoint inhibitors can enhance anti-tumor immune responses. The good therapeutic effects of ICIs in various solid tumors indicate that ICIs are promising therapeutic approach, warranting further research. Given the association of BTC with chronic inflammation, patients with BTC may be a potential target population for ICIs.

- - 1. Immune Microenvironment in Biliary Tract Carcinoma

BTC is a desmoplastic tumor. The immune microenvironment of BTC consists of cancer-associated fibroblasts and immunosuppressive innate immune cells, such as tumor-associated macrophages and myeloid-derived suppressor cells, with generally low levels of natural killer (NK) cells**^26^**. To avoid severe inflammatory responses due to continuous exposure to gut flora and other antigens from the digestive system, the liver maintains a state of chronic immune tolerance, which is partially mediated by Kupffer cells**^27^**. The unique features of BTC immune microenvironment may compromise the treatment response to ICIs such as by inducing fibrosis , which limits the penetration of drugs or immune cells into tumor sites; Tumor-associated macrophages and dendritic cells expressing immune tolerance factors such as PD-L1 can be exploited by tumor cells to upregulate immune checkpoints, including PD-1, TIGIT, CTLA-4, LAG-3, IDO-1, and TIM-3, further resulting in T-cell exhaustion and promoting immunosuppression and progression of BTC**^28-29^**. Thus, antibody therapies that antagonize these checkpoints can restore immune activity of host cells.

- - 1. Clinical Studies of Immune Checkpoint Inhibitor Monotherapy for Advanced BTC

Phase 1 study KEYNOTE-028 assessed the antitumor activity of pembrolizumab in 20 different advanced solid tumors**^30^**. The study finally enrolled 24 BTC patients, all were PD-Ll positive and received 10 mg/kg pembrolizumab every 2 weeks. A total of 23 patients were efficacy evaluable, among them, 3 patients achieved PR with an ORR of 13.0% (3/23, 95% CI, 2.8% to 33.6%). The median PFS and median OS was 1.8 months (95% CI: 1.4 to 3.7 months) and 6.2 months (95% CI: 3.8 to 10.3 months), respectively. The 12-month OS rate was 27.6%. The following phase 2 study KEYNOTE-158 **^31^** enrolled a total of 104 patients with advanced BTC, 61 of whom were PD-L1 positive (≥ 1%), and none were MSI-H. Patients received pembrolizumab 200 mg, Q3W. Six patients eventually achieved PR (including 1 patient who was PD-L1 negative) with an ORR of 5.8% (6/104; 95% CI, 2.1% to 12.1%). The median PFS and median OS were 2.0 months (95% CI: 1.9 to 2.1 months) and 7.4 months (95% CI: 5.5 to 9.6 months), respectively. The 12-month OS rate was 32.7%. In study KEYNOTE-158, pembrolizumab demonstrated effective and durable antitumor activity regardless of PD-L1 status (positive vs. negative). Taking the phase 1 and phase 2 studies together, it can be concluded that pembrolizumab demonstrated effective antitumor activity in advanced BTC patients regardless of PD-L1 status.

In a phase 2 clinical study evaluating nivolumab for the treatment of advanced BTC conducted in the United States, 54 patients with refractory advanced BTC (who had progressed after ≥1 line of treatment) were enrolled, including 32 patients with intrahepatic cholangiocarcinoma, 5 patients with extrahepatic cholangiocarcinoma, and 17 patients with gallbladder cancer. The investigator-assessed objective response rate was 22% (10/46), all of which were PR, and the DCR was 59% (27/46). For evaluable patients, PFS was 3.68 months (95% CI, 2.30 to 5.69 months), and median OS was 14.24 months (95% CI, 5.98 months to NA). PD-L1 positive tumor cells (defined as PD-L1 ≥1% in tumor cells by immunohistochemichal staining) was associated with prolonged PFS (HR = 0.23, 95% CI, 0.10 to 0.51, P < 0.001). In a clinical trial evaluating nivolumab conducted in Japan, the objective response rate in the monotherapy arm was only 3% (1/30), the median OS was 5.2 months, and the PFS was 1.4 months**^33^**.

- - 1. ICIs-containing Combination Therapy for BTC

To improve treatment response and enhance the efficacy of ICIs in wider population, an increasing number of clinical trials are exploring the combination of ICIs with other treatments to increase the exposure of tumor-associated antigens, enhance T-cell activation, reduce immunosuppressive factors, and modulate the immune microenvironment. These combination therapy include immunochemotherapy, dual immunotherapy, immunotherapy combined with local treatments, and immunotherapy combined with targeted therapy, which are mostly in the early stages of clinical trials.

**ICIs in Combination with Chemotherapy**

Currently, chemotherapy is the backbone treatment for BTC recommended in both China and international treatment guidelines. Chemotherapeutic drugs such as as gemcitabine, cisplatin, and 5-fluorouracil can upregulate the expression of immune checkpoints, alter immune cell infiltration, and enhance cell-mediated immune responses by increasing the expression of human leukocyte antigen (HLA) on BTC cells**^34-35^**. The combination of ICIs with chemotherapy warrants further attention.

In a multicenter, open-label, phase 1 clinical trial evaluating nivolumab in combination with gemcitabine/cisplatin (GP) in advanced unresectable or metastatic BTC conducted in Japan, 30 patients were enrolled in the combination arm to receive nivolumab in combination with GP as first line treatment, the study reported a PR rate of 37%, a median OS of 15.4 months (95% CI, 11.8 months to NA), and a median PFS of 4.2 months (95% CI, 2.8 to 5.6 months)**^33^**.

In 2020, a phase 2 study of anti-PD-L1 mAb, durvalumab (D) with/without anti-CTLA-4 mAb tremelimumab (T) in combination with gemcitabine/cisplatin (GP) as first-line treatment in patients with advanced BTC was reported on ASCO**^36^**; the study consisted of three cohorts: biomarker cohort (BMC, n = 30), GP + D (n = 45), and GP + D + T (n = 45). The median follow-up time for each cohort was 28.5 months, 11.3 months, and 11.9 months, respectively. The results showed that the primary endpoint ORR was 50.0%, 73.4%, and 73.3% for BMC, GP + D and GP + D + T cohort, respectively; DCR was 96.7%, 100.0%, and 97.8%, respectively; DoR was 11.0 months, 9.8 months, and 9.1 months, respectively; PFS was 13 months, 11.0 months, 11.9 months, respectively; OS was 15.0 months, 18.1 months, and 20.7 months, respectively. In terms of safety, common adverse reactions were neutropenia (54.5%), nausea (59.5%) and pruritus (55.44%). The common Grade 3 or Grade 4 AEs were neutropenia (50.4%), anemia (35.5%), and thrombocytopenia (16.5%). The GP + D regimen is being tested in a phase 3 clinical study (TOPAZ-1, NCT03875235).

A single-arm, open-label, phase 2 clinical trial has evaluated efficacy and safety of camrelizumab in combination with gemcitabine/oxaliplatin (GEMOX) in advanced BTC. Thirty-eight patients with advanced BTC were enrolled. The median follow-up time was 11.8 months. The PFS rate was 50% at 6 months, the ORR was 54%, the median PFS was 6.1 months, and the median OS was 11.8 months. Patients with PD-L1 tumor proportion score (TPS) ≥ 1% had an ORR of 80%, while those with PD-L1 TPS < 1% had an ORR of 53.8%. The most commonly reported treatment‑related adverse events (TRAEs) were fatigue (73%) and pyrexia (73%). The most commonly reported TRAE of ≥ Grade 3 were hypokalaemia (19%) and fatigue (16%)**^37^**.

In 2021, an open-label, phase 2 clinical trial (JS001-ZS-BC001) evaluating safety and efficacy of toripalimab in combination with gemcitabine/S1 (GS) as first-line treatment for advanced BTC was reported on ASCO meeting. Fifty (50) patients with BTC were enrolled, with a median follow-up duration of 10 months (range: 4 to 19 months). The median PFS was 7.0 months and median OS was 16.0 months. The ORR was 27.1% and the DCR was 87.5%; 13 patients achieved partial response (PR) and 29 patients achieved stable disease (SD). The most commonly observed TRAEs were neutropenia (92.0%), anaemia (86.0%) and rash (52.0%)**^38^**.

In 2021, an open-label phase 2 clinical trial sponsored by Zhongshan Hospital affiliated to Fudan University, evaluating efficacy and safety of toripalimab in combination with lenvatinib and GEMOX regimen in patients with unresectable locally advanced or metastatic ICC was also reported on ASCO meeting. The study enrolled 30 patients with locally advanced or metastatic ICC. The median follow-up time was 16.6 months. The results showed that 1 patient achieved complete response (CR). The primary endpoint ORR was 80% (24/30) and DCR was 93.3% (28/30). Twenty-three (23) patients reported progressive disease and 12 patients (including 1 patient withdrawn from the study) died. The median PFS was 10.0 months and median DoR was 9.8 months. The median OS was not reached. The 12-month OS rate was 73.3%. The ORR was significantly correlated with PD-L1 expression (p = 0.048) and DNA damage repair (DDR) -related mutations (p = 0.022) in tumor tissues. These findings warrant further validation in large, randomized clinical trials**^39^**.

**ICIs in Combination with Other Treatments**

Other ICIs-containing combination regimens, including dual ICIs, such as rdurvalumab in combination with anti-CTLA-4 inhibitors**^40^**, ICI in combination with targeted therapy, scuh as pembrolizumab plus lenvatinib **^41^** and ICIin combination with local therapy, such as tremelimumab plus tumor microwave ablation**^42^** have also been explored in patients with advanced BTC, offering potential treatment options.

Background Information on Tislelizumab

- 1. Pharmacology

Tislelizumab (also known as BGB-A317) is a humanized, immunoglobulin IgG4 variant monoclonal antibody against PD-1 under clinical development for the treatment of several human malignancies.

Tislelizumab acts by binding to the extracellular domain of human PD-1 with high specificity and affinity (dissociation constant [KD] = 0.15 nM). It competitively blocks binding efforts by both programmed cell death protein ligand-1 (PD‑L1) and programmed cell death protein ligand-2 (PD‑L2), thus inhibiting PD-1-mediated negative signaling in T cells. In *in vitro* cell-based assays, tislelizumab was observed to consistently and dose-dependently enhance the functional activity of human T cells and pre‑activated primary peripheral blood mononuclear cells. In addition, tislelizumab has demonstrated anti-tumor activity in several allogeneic xenograft models, in which peripheral blood mononuclear cells were co-injected with human cancer cells (A431 [epidermoid carcinoma]) or tumor fragments (BCCO-028 [colon cancer]) into immunocompromised mice.

Tislelizumab is an IgG4-variant antibody to gamma fragment crystallizable region (Fc) receptors (FcγR) such as FcγRI and FcγRIIIA, and has very low binding affinity to complement 1q (C1q), a subunit of complement 1. *In vitro* assays with tislelizumab suggested either low or no antibody-dependent cellular cytotoxicity (ADCC), antibody-dependent cellular phagocytosis (ADCP), or complement-dependent cytotoxicity (CDC) effects in humans**^43-44^**.

Please refer to the Tislelizumab Investigator’s Brochure **(IB)** for additional details regarding nonclinical studies of tislelizumab.

- 1. Toxicology

The toxicity and safety profile of tislelizumab was characterized in single-dose toxicology studies in mice and monkeys and in a 13-week, repeat-dose toxicology study in cynomolgus monkeys. The tissue cross-reactivity was evaluated in the normal frozen tissues from both humans and monkeys. The cytokine release assays were also evaluated using fresh human whole blood cells. The pivotal toxicology studies were conducted following Good Laboratory Practice (GLP) regulations. The single-dose regimens spanned from the intended human dose to 10-fold higher than the maximum of the intended human dose, and the repeated-dose regimens spanned the intended human dose to to 3-fold higher than the maximum of the intended human dose. Cynomolgus monkey was the only relevant species based on target sequence homology and binding activity.

Overall, no apparent toxicity was noted in mice or monkey toxicity studies. No tissue cross-reactivity was found in either human or monkey tissues, nor was any effect on cytokine release observed in human whole-blood assay. The toxicokinetic profile was well characterized, with dose proportional increases in systemic exposure without apparent accumulation or sex difference. Immunogenicity was observed without apparent immunotoxicity and effect on the systemic exposure. The No Observed Adverse Effect Level (NOAEL) of tislelizumab in the 13‑week monkey toxicity study was considered to be 30 mg/kg.

Please refer to the Tislelizumab Investigator’s Brochure **(IB)** for more detailed information on the toxicology of tislelizumab.

- 1. Clinical Pharmacology

In the phase 1 studies of BGB-A317_Study_001 and Study BGB-A317-102, interim pharmacokinetics (PK) analysis (data cutoff date 28 August 2017) was conducted by noncompartmental methods, using serum concentrations from patients who received doses of 0.5, 2.0, 5.0, 10 mg/kg once every 2 weeks (Q2W) and 2.0 mg/kg, 5.0 mg/kg, 200 mg Q3W (phase 1a parts 1, 2, and 3, and phase 1b in BGB-A317_Study_001) and patients who received doses of 200 mg Q3W in phase 1 of Study BGB-A317-102 (n = 19). The C_max_ and AUC increased in a nearly dose-proportional manner from 0.5 mg/kg to 10 mg/kg, both after single-dose administration and at steady state. Preliminary PK data from 27 patients who were administered 1 dose of 200 mg Q3W (phase 1a, part 3 and study BGB-A317-102) showed tislelizumab concentrations between the range of concentrations observed for patients who were administered 2 mg/kg and 5 mg/kg doses.

Preliminary population PK analysis using a 2-compartment model with first-order elimination shows a systemic plasma clearance (CL) of tislelizumab of 0.164 L/day, volume of distribution (Vd) in the central and peripheral compartments of 2.92 L and 1.39 L, respectively, and half-life (t_1/2_) of approximately 25.9 days. Race, gender, and body weight were not significant covariates on the CL of tislelizumab, which supports fixed-dosing across different ethnic groups.

- 1. Prior Clinical Experience With Tislelizumab

As of 20 May 2020, there are 28 ongoing studies with tislelizumab. Of the 28 ongoing studies, 15 have preliminary data available for presentation in the Investigator’s Brochure (IB): 7 monotherapy studies (BGB-A317_Study_001, BGB-A317-102, BGB-A317-203, BGB-A317-204, BGB-A317-207, BGB-A317-208, as well as BGB-A317-209); 2 chemotherapy combination therapy studies (BGB-A317-205 and BGB-A317-206); 6 targeted therapy combination therapy studies (BGB-A317/BGB-290_Study_001 [tislelizumab in combination with BGB-290 (also known as pamiparib, a poly (adenosine diphosphate ribose) polymerase [PARP] inhibitor), BGB-3111_BGB-A317_Study_001 [tislelizumab incombination with zanubrutinib (also known as BGB-3111, a Bruton tyrosine kinase inhibitor], BGB-900-101 [BGB-A333 (an anti-PD-L1 monoclonal antibody) monotherapy and in combination with tislelizumab], BGB-900-102 [BGB-A425, a humanized immunoglobulin gamma-1 (IgG1) variant monoclonal antibody against TIM-3 in combination with tislelizumab], BGB-900-104 (tislelizumab in combination with sitravatinib).

In addition, there are 13 ongoing studies, most of which are pivotal phase 3 studies (study BGB-A317-301, BGB-A317-302, BGB-A317-303, BGB-A317-304, BGB-A317-305, BGB-A317-306, BGB-A317-307, BGB-A317-309, BGB-A317-310, BGB-A317-311, BGB-A317-312, and BGB-A317-290-LTE1; and study BGB-900-105).

Data are available for the following studies: BGB-A317_Study_001, BGB-A317-102, BGB-A317-203, BGB-A317-204, BGB-A317-207, BGB-A317-208, and BGB-A317-209, which are summarized below:

Please refer to the Tislelizumab Investigator’s Brochure [Tislelizumab Investigator’s Brochure](file:///C:\Users\butte\Desktop\A317-307方案修订标记版倒腾草稿.docx#_BeiGene_Investigator’s_Brochure,)**(IB)** for more detailed information on efficacy and safety of tislelizumab.

- - 1. Study BGB-A317_Study_001 (Monotherapy)

BGB-A317_Study_001 is a 2-stage study. Phase 1A consists of a dose escalation and dose-finding component to establish the maximum tolerated dose (MTD), if any, and the recommended phase 2 dose/doses (RP2D). The primary objective of phase 1A is to assess the safety and tolerability of tislelizumab in patients with advanced tumors. The phase 1B is designed to investigate efficacy in select tumor types and to further evaluate the safety and tolerability of tislelizumab at the selected dose. The primary objective of phase 1B is to assess the antitumor activity of tislelizumab in select tumors.

As of 27 October 2017, the study was fully enrolled. Data are available for 451 treated patients (116 patients in phase 1A and 335 patients in phase 1B).

MTD was not reached with the maximum-administrated dose of 10 mg/kg Q2W in the dose-escalation part. Only 1 Grade 3 colitis occurred with 5 mg/kg Q2W dosing cohort.

There are 451 patients treated in the study and 441 patients are included in the efficacy evaluable set. Across all disease cohorts (N = 441), 5 patients (1.1%) have achieved complete response (CR), 55 patients (12.5%) have achieved partial response (PR), and 142 patients (32.2%) have achieved stable disease, yielding an overall response rate (ORR) of 13.6% and DCR of 45.8%.

Of the 49 patients in the HCC disease cohort, 6 patients (12.2%) have achieved PR and 19 patients (38.8%) have achieved stable disease, yielding an ORR of 12.2% and DCR of 51%.

- - 1. Study BGB-A317_Study_102 (Monotherapy)

Study BGB-A317-102 is a 2 phases, non-randomized, phase 1/2 study of tislelizumab monotherapy in Chinese patients with advanced solid tumors. The dose‑verification substudy of phase 1 has assessed the safety and tolerability of tislelizumab in patients with advanced solid tumors and confirmed the MTD and RP2Ds in Chinese patients. The PK substudy of phase 1 has assessed the PK of products derived from 2 manufacturing processes and scales (500L-FMP and 2000L FMP) and to assess the safety and tolerability of tislelizumab of the above mentioned manufacturing processes and scales in patients with advanced solid tumors. The phase 2 indication expansion study consists of 11 arms of indications of special interest to assess the efficacy, safety and tolerability of tislelizumab in Chinese patients with malignant solid tumors. In the phase 2 study, tislelizumab has been administered at a dose of 200 mg IV Q3W, which had been previously determined to be well-tolerated in the Chinese population.

As of 31 May 2018, the study was fully enrolled. Data are available from 300 patients treated on study. Overall, 249 patients are included in the Efficacy Evaluable Analysis Set. The Efficacy Evaluable Analysis Set includes all treated patients who have at least 1 measurable baseline target lesion and have at least 1 evaluable postbaseline tumor assessment.

No DLTs occurred in phase 1 dose-verification portion of study BGB-A317-102. The dose of 200 mg Q3W has been confirmed as the RP2D.

Across all disease cohorts (N = 249), 1 patient (0.4%) has achieved CR, 44 patients (17.7%) have achieved PR, and 91 patients (36.5%) have achieved stable disease, yielding an ORR of 18.1% and DCR of 54.6%.

Of the 16 patients in the HCC disease cohort, 3 patients (18.8%) have achieved PR and 7 patients (43.8%) have achieved stable disease, yielding an ORR of 18.8% and DCR of 62.5%.

- - 1. Study BGB-A317_Study_203 (Monotherapy)

Study BGB-A317-203 is a single-arm, multicenter, phase 2 study evaluating tislelizumab monotherapy in patients with relapsed or refractory classical Hodgkin's lymphoma in China. The primary objective of the study is to evaluate the efficacy of tislelizumab in in patients with relapsed or refractory classical Hodgkin's lymphoma in China. Additionally, safety, PK, and immunogenicity are currently under assessment. Approximately 68 patients are planned to be enrolled to receive tislelizumab at the dose of 200 mg IV Q3W. As of 22 November 2017, enrollment for this study has been completed and data are available from 70 patients treated on study. Safety results from study BGB-A317-203 are included in the IB as part of the integrated analysis of monotherapy.

- - 1. Study BGB-A317_Study_204 (Monotherapy)

Study BGB-A317-204 is a single-arm, open-label, multicenter phase 2 study evaluating safety of tislelizumab monotherapy in patients with previously treated, PD-L1+, locally advanced or metastatic urothelial bladder cancer. The primary objective of this study is to evaluate the efficacy of tislelizumab in patients with previously treated, PD-L1+, locally advanced or metastatic urothelial bladder cancer. Additionally, safety, PK, immunogenicity as well as potential predicative biomarkers are currently under assessment. Tislelizumab has been administered at a fixed-dose of 200 mg, IV, Q3W. Approximately 110 patients are enrolled. As of 31 August 2018, the study was fully enrolled. Data are available from 113 patients treated on study. Safety results from study BGB-A317-204 are as part of the integrated analysis of monotherapy.

- - 1. Study BGB-A317_Study_207 (Monotherapy)

Study BGB-A317-207 is an open-label, non-randomized, multicenter, prospective, phase 2 study evaluating efficacy of tislelizumab monotherapy in patients with relapsed or refractory mature T- and natural killer (NK)-cell neoplasms. The primary objective of this study is to evaluate the efficacy of tislelizumab in patients with relapsed or refractory mature T- and NK-cell neoplasms. Additionally, safety, PK, immunogenicity, potential predicative biomarkers as well as circulating EBV DNA levels are currently under assessment.

Patients are divided into 3 cohorts: cohort 1 includes patients with relapsed or refractory extranodal NK/T cell lymphoma (nasal or non-nasal type), excluding patients with aggressive NK leukemia; cohort 2 includes patients with relapsed or refractory mature T-cell tumors, limited to the following histologies; peripheral T-cell lymphoma- not otherwise specified (NOS), angioimmunoblastic T-cell lymphoma, and anaplastic large cell lymphoma; and cohort 3 includes patients with relapsed or refractory stage IB-IVB cutaneous T-cell lymphoma, limited to patients with mycosis fungoides or Sézary syndrome. Tislelizumab has been administered at a fixed-dose of 200 mg, IV, Q3W.

As of 20 May 2020, enrollment for cohort 1 was completed and 22 patients have been treated on study. Up to 50 patients are planned to be enrolled in cohort 2, and up to 10 patients are planned to be enrolled in cohort 3, and the overall sample size will be up to approximately 80 to 85 patients. The study is currently in the enrollment phase. As of 20 May 2020, data are available from 77 patients treated on study.

Safety results from study BGB-A317-207 are included in the IB as part of the integrated analysis of monotherapy. Preliminary efficacy data will be presented separately in the IB.

- - 1. Study BGB-A317_Study_208 (Monotherapy)

Study BGB-A317-208 is an open-label, single-arm, multicenter phase 2 study evaluating efficacy of tislelizumab monotherapy in patients with previously treated unresectable hepatocellular carcinoma (HCC). The primary objective of this study is to evaluate the efficacy of tislelizumab monotherapy in patients with unresectable HCC who had received systemic therapy. Additionally, safety, tolerability, PK, immunogenicity as well as potential predicative biomarkers are currently under assessment. Tislelizumab has been administered at a fixed-dose of 200 mg, IV, Q3W.

As of 27 February 2019, the study was fully enrolled. Data are available from all 249 patients treated on study.

Preliminary efficacy data have been submitted for new drug application (NDA) review and are therefore not included in the IB.

- - 1. Study BGB-A317_Study_209 (Monotherapy)

Study BGB-A317-209 is an open-label, single-arm, multicenter, phase 2 study evaluating efficacy and safety of tislelizumab monotherapy in patients with previously treated, locally advanced unresectable or metastatic microsatellite instability-high (MSI-H) or mismatch repair deficient (dMMR) solid tumors. The primary objective of this study is to evaluate the efficacy and safety of tislelizumab monotherapy in patients with previously-treated locally advanced unresectable or metastatic MSI-H/dMMR solid tumors. Additionally, DoR, time to response, DCR, PFS, safety, tolerability, PK, immunogenicity, and potential predictive biomarker(s) are currently under assessment. Tislelizumab has been administered at a fixed-dose of 200 mg, IV, Q3W.

The study plans to enroll to 79 patients. The study is currently in the enrollment phase. As of 20 May 2020, data are available from 68 patients treated on study.

Preliminary safety results from study BGB-A317-209 are as part of the integrated analysis of monotherapy; currently, while currently, however, preliminary efficacy and PK data are not available.

- 1. Pooled Safety Assessment of Monotherapy Studies

A pooled analysis of monotherapy studies has been conducted to provide a comprehensive safety assessment separate from combination therapy. To enhance the analyses and interpretation of the monotherapy safety profiles, safety results from the relevant studies are presented based on the underlying tumor types included in the study, either as solid tumor or hematologic malignancy. The pooled data for the total population across all monotherapy studies are tabulated in the following section. All the monotherapy studies included in the pooled safety analysis are presented in Table 1.

Table 1: Pooled Tislelizumab Monotherapy Studies

|  | Study Code | Number of patients enrolled (as of May 20, 2020) | Study Design |
| --- | --- | --- | --- |
| Monotherapy to solid tumor | BGB-A317-001 | 451 (Enrollment complete) | Section 2.4.1 |
|  | BGB-A317-102 | 300 (Enrollment complete) | Section 2.4.2 |
|  | BGB-A317-204 | 113 (Enrollment complete) | Section 2.4.4 |
|  | BGB-A317-208 | 249 (Enrollment complete) | Section 2.4.6 |
|  | BGB-A317-209 | 68 (Enrollment ongoing) | Section 2.4.7 |
| Monotherapy to hematologic malignancy | BGB-A317-203 | 70 (Enrollment complete) | Section 2.4.3 |
|  | BGB-A317-207 | 77 (Enrollment complete) | Section 2.4.5 |
|  | Total | 1328 |  |

Relevant studies are presented by the underlying tumor types included in the study (either as solid tumor or hematologic malignancy) and by pooled population.

There are 1181 patients treated in 5 pooled solid tumor monotherapy studies. Of these 1181 patients, 283 patients (24.0%) remain on study as of 20 May 2020, 133 patients (11.3%) are still receiving tislelizumab treatment, and 150 patients (12.7%) are in follow-up.

At the time of data cutoff, 719 patients have received study treatment for ≥ 2 months, thus providing sufficient data for evaluation. Among these 719 patients, 130 (18.1%) patients are still receiving tislelizumab treatment.

There are 147 patients treated in 2 pooled hematologic malignancy monotherapy. Of these 147 patients, 85 patients (57.8%) remain on study as of 20 May 2020, 56 patients (38.1%) are still receiving tislelizumab treatment, and 29 patients (19.7%) are in follow-up. At the time of data cutoff, 120 patients have received treatment for ≥ 2 months, thus providing sufficient data for evaluation. Among these 120 patients, 56 (46.7%) patients are still receiving tislelizumab treatment.

- - 1. DLTs Occurred in Dose-Escalation and Determination of MTDs

**Study-BGB-A317-001**

A DLT is defined as any adverse event or abnormal laboratory finding that occurred within the first 28 days, is determined to be unrelated to disease progression, intercurrent illness, or concomitant medications, and meets any of the following criteria listed in the study protocol.

The maximum administered dose in the dose-escalation part of the study is 10 mg/kg Q2W. The MTD was not identified. Only 1 DLT of Grade 3 colitis occurred with 5 mg/kg Q2W schedule. Based on the data from 103 patients enrolled in the dose escalation and dose expansion parts of this phase 1A study, 5 mg/kg Q3W has been selected for phase 1B study to investigate the anticancer activity of tislelizumab against multiple tumor types. The safety, PK, and preliminary efficacy of a fixed dose of 200 mg Q3W that did not exceed the exposure of the MTD have been further evaluated.

**Study-BGB-A317-102**

The occurrence of any of the severe toxicities listed in the study protocol during the first 21 days is considered a DLT, if judged by the investigator to be possibly, probably, or definitely related to study treatment administration.

No DLTs occurred in phase 1 dose-verification portion of study BGB-A317-102. The dose of 200 mg Q3W was confirmed as the RP2D.

- - 1. Treatment-Emergent Adverse Events As Assessed Related to Monotherapy

As of 20 May 2020, of the 1181 patients in the solid tumor group of pooled monotherapy studies, 788 (66.7%) experienced ≥ 1 treatment-related TEAE(s). TEAEs related to tislelizumab monotherapy reported in ≥ 5% of patients with solid tumors are presented in **Table 2** below.

Table 2: Treatment-related TEAEs Occuring in ≥ 5% of Patients in Solid Tumor Group of Pooled Tislelizumab Monotherapy Studies

| Adverse Event | Tislelizumab |
| --- | --- |
| AST increased | 136 (11.5%) |
| ALT increased | 125 (10.6%) |
| Hypothyroidism | 106 (9.0%) |
| Rash | 97 (8.2%) |
| Pruritus | 95(8.0%) |
| Fatigue | 95(8.0%) |
| Anemia | 89 (7.5 %) |
| Blood bilirubin increased | 77 (6.5%) |
| Diarrhea | 71 (6.0%) |
| Decreased appetite | 65(5.5%) |
| Proteinuria | 60(5.1%) |
| Nausea | 59(5.0%) |
| ≥ 1 treatment-related TEAE(s) of ≥ Grade 3 | 167 (14.1%) |
| Treatment-related TEAEs of ≥ Grade 3 occurring in ≥ 1% of patients |  |
| AST increased | 21 (1.8%) |
| ALT increased | 17 (1.4%) |
| Anemia | 13 (1.1%) |

As of 20 May 2020, of the 147 patients in the hematologic malignancy group of pooled monotherapy studies, 122 (83.0%) experienced at least 1 treatment-related TEAE. The most common treatment-related TEAEs are presented in **Table 3** below.

Table 3: Treatment-related TEAEs Occuring in ≥ 5% of Patients in Hematologic Malignancy Group of Pooled Tislelizumab Monotherapy Studies

| Adverse Event | Tislelizumab |
| --- | --- |
| Pyrexia | 48 (32.7%) |
| Hypothyroidism | 33 (22.4%) |
| Pruritus | 20 (13.6%) |
| White blood cell count decreased | 18 (12.2%) |
| ALT increased | 15 (10.2%) |
| Neutrophil count decreased | 13 (8.8%) |
| AST increased | 11(7.5%) |
| Platelet count decreased | 11 (7.5%) |
| Anemia | 11(7.5%) |
| Blood thyroid stimulating hormone increased | 10 (6.8%) |
| Asthenia | 10 (6.8%) |
| Blood creatine phosphokinase increased | 9 (6.1%) |
| Rash | 9 (6.1%) |
| Blood bilirubin increased | 8 (5.4%) |
| Thrombotyopenia | 8 (5.4%) |
| Arthralgia | 8 (5.4%) |
| ≥ 1 treatment-related TEAE(s) of ≥ Grade 3 | 30 (20.4%) |
| Treatment-related TEAEs of ≥ Grade 3 occurring in ≥ 1% of patients |  |
| Neutrophil count decreased | 4 (2.7%) |
| White blood cell count decreased | 3 (2.0%) |
| Anemia | 3 (2.0%) |
| Neutropenia | 3 (2.0%) |
| Pneumonia | 3 (2.0%) |
| Lipase increased | 2 (1.4%) |
| Blood creatine phosphokinase increased | 2 (1.4%) |
| Platelet count decreased | 2 (1.4%) |
| Pneumonitis | 2 (1.4%) |
| Hyponatraemia | 2 (1.4%) |
| Pyrexia | 2 (1.4%) |

- - 1. Special Categories of Immune Related Adverse Events Reported in Monotherapy Studies

As of 20 May 2020, of the 1113 patients in the adjudicated solid tumor group for the pooled monotherapy studies, 233 (20.9%) experienced at least 1 immune-related adverse event (irAE) of any grade. Analysis of the patients with at least 1 irAE that also was ≥ Grade 3 in severity within the solid tumor group showed that 52 patients (4.7%) experienced such events. The most commonly reported irAEs are presented in **Table 4** below.

Table 4: Immune-Related Adverse Events of Any Grade Occurring in ≥ 1% of Patients in Solid Tumor Group of Tislelizumab Monotherapy Studies (Safety Analysis Set)

| Adverse Event | Tislelizumab |
| --- | --- |
| Hypothyroidism | 69 (6.2%) |
| Hyperthyroidism | 37 (3.3%) |
| Rash | 34 (3.1%) |
| Pneumonitis | 19 (1.7%) |
| Pruritus | 17 (1.5%) |
| ALT increased | 14 (1.3%) |
| AST increased | 11 (1.0%) |
| ≥ 1 irAE(s) of ≥ Grade 3 | 52 (4.7%) |
| ≥ Grade 3 irAEs Occurring in ≥ 1% of patients |  |
| Immune-related hepatitis | 18 (1.6%) |
| ALT increased | 14 (1.3%) |
| AST increased | 11 (1.0%) |

As of 20 May 2020, of the 70 patients in the adjudicated hematologic malignancy group for the pooled monotherapy studies, 27 (38.6%) experienced at least 1 irAE of any grade. Analysis of the patients with at least 1 irAE that also was ≥ Grade 3 in severity within the hematologic malignancy group showed that 8 patients (11.4%) experienced such events. The most commonly reported irAEs are presented in **Table 5** below.

Table 5: Immune-Related Adverse Events of Any Grade Occurring in ≥ 1% of Patients in Hematologic Malignancy Group of Tislelizumab Monotherapy Studies (Safety Analysis Set)

| Adverse Event | Tislelizumab |
| --- | --- |
| Hypothyroidism | 15 (21.4) |
| Pruritus | 3 (4.3) |
| Hyperthyroidism | 2 (2.9) |
| Pneumonitis | 2 (2.9) |
| Dermatitis | 1 (1.4) |
| ≥ 1 irAE(s) of ≥ Grade 3 | 8 (11.4) |
| ≥ Grade 3 irAEs occurring in ≥ 1% of patients |  |
| Pneumonitis | 2 (2.9) |
| Blood creatine phosphokinase increased | 1 (1.4) |

- - 1. Fatal Adverse Events Reported in Monotherapy Studies

As of 20 May 2020, 88 patients (6.6%) experienced TEAEs leading to death. A total of 104 patients (8.8%) within the pooled solid tumors monotherapy studies died within 30 days of the last study treatment dose. Of these 104 patients, 18 patients (1.5%) experienced a fatal AE within 30 days of the last study treatment dose. Besides, 683 (57.8%) patients died beyond 30 days of the last study treatment dose; 8 of these deaths (0.7%) were due to an AE.

A total of 3 patients (2.0%) within hematologic malignancy monotherapy studies died within 30 days of the last study treatment dose. All of these patients (3, 2.0%) had a fatal AE within 30 days of the last study treatment dose. Of the 40 patients (27.2%) who died more than 30 days after the last study treatment dose, 12 of these deaths (8.2%) were due to an AE.

- 1. Pooled Efficacy Assessment of Monotherapy Studies
     1. Study BGB-A317-001

BGB-A317_Study_001 is a 2-phase study. Phase 1A consists of a dose-escalation and dose finding component, and phase 1B evaluates efficacy and safety in select tumor types.

There are 451 patients treated in the study and 441 patients are included in the Efficacy Evaluable Set. Responses are assessed by the investigator per the RECIST v1.1.

Across all disease cohorts, 5 patients (1.1%) have achieved CR. A total of 55 patients (12.5%) have achieved confirmed PR. The overall clinical response rate is 13.6%. Additionally, 142 patients (32.2%) have achieved a best overall response of stable disease (SD). A total of 199 patients (45.1%) have achieved best response of PD in this study.

Of the 49 patients in the HCC disease cohort, 6 patients (12.2%) have achieved PR and 19 patients (38.8%) have achieved stable disease, yielding an ORR of 12.2% and DCR of 51%.

- - 1. Study BGB-A317-102

Study BGB-A317-102 is a two-phase, non-randomized, phase 1/2 study of tislelizumab monotherapy in Chinese patients with advanced solid tumors. Phase 1 includes a dose verification substudy and a substudy of PK evaluation of the products derived from 2 manufacturing processes and scales. Phase 2 is an indication‑expansion study. Responses are assessed by the investigator per the RECIST v1.1.

Overall, of the 300 patients treated in study BGB-A317-102, 249 patients are included in the Efficacy Evaluable Analysis Set. The Efficacy Evaluable Analysis Set includes all treated patients who have ≥ 1 measurable baseline target lesion and have ≥ 1 evaluable post-baseline tumor assessment.

Across all disease cohorts and study phases, 1 patient (0.4%) has achieved CR. A total of 44 patients (17.7%) have achieved confirmed PR. The overall clinical response rate is 18.1%. Additionally, 91 patients (36.5%) have achieved best overall response of SD. A total of 113 patients (45.4%) have achieved best response of PD in this study. The disease control rate is 54.6%.

Of the 16 patients in the HCC disease cohort, 3 patients (18.8%) have achieved PR and 7 patients (43.8%) have achieved stable disease, yielding an ORR of 18.8% and DCR of 62.5%.

- 1. Clinical Safety of Tislelizumab

The safety profile of tislelizumab is consistent with the therapeutic class of the drug with a relatively low rate of drug-related ≥ Grade 3 toxicity.

Across the monotherapy studies, the safety profile is consistent in the phase 1and phase 2 studies. Over half of the patients in these studies experienced a treatment-related TEAE, though treatment-related ≥ Grade 3 events were lower (14.1%). Immune-related AEs of any grade have been reported in approximately 22% of patients but are primarily low grade (5.1% ≥ Grade 3). These irAEs, however, have well‑established algorithms for treatment and are therefore considered manageable.

In studies with tislelizumab plus chemotherapy, the AE profile is consistent with the now well‑established profile of ICIs in combination with the standard chemotherapy agents. Over 80% of patients in these studies experienced a treatment‑related TEAE, though treatment-related ≥ Grade 3 events were lower (20.4%).

When combined with another investigational agent, the safety profile of the combination therapy is generally consistent with the safety profiles of each drug given as monotherapy or consistent with the safety profile of the same class agent combination. Overall, the safety profile is found to be well tolerated which has remained consistent with the expected safety profile of the same class agent combination. Over 60% of patients in these studies experienced a treatment‑related (TEAE), though treatment-related ≥ Grade 3 events were lower (3.8% to 38.7%). However, these data should be interpreted with some caution because the sample sizes are relatively small for the ongoing studies.

**Adverse Events and Serious Adverse Events With a Fatal Outcome**

In the monotherapy studies, the incidence of treatment-emergent SAEs is 37.4% across patients with different disease characteristics. Treatment-related treatment‑emergent SAEs are notably lower, with a rate of 19.0%.

In studies with tislelizumab combined with chemotherapy, the treatment-emergent SAEs rate is 34.5%. Treatment-related treatment‑emergent SAEs are notably lower, with a rate of 16.7%. When combined with another investigational agent, the incidence of treatment-emergent SAEs ranges from 33.3% to 64.0%, while the incidence of treatment-related treatment-emergent SAEs ranges from 3.8% to 28.0%. However, these data should be interpreted with some caution because the sample sizes are relatively small for some individual studies.

In all ongoing studies with available clinical data, 121 TEAEs leading to death (accounting for 6.3% of the overall population [n = 1917]) have been reported as of 20 May 2020, of which 18 (0.1%) are assessed as related to tislelizumab. The incidence of TEAEs leading to death is low overall across studies, and the AEs associated with an outcome of death are consistent with what has been reported for the same drug class.

For more detailed information on the safety of tislelizumab, refer to the **Tislelizumab IB**.

- 1. Rationale for the Selection of the Tislelizumab Dose

The PK, safety, and efficacy data obtained from the first-in-human study BGB‑A317_study_001, as well as other clinical study data, have been analyzed in aggregate to determine the recommended dose for pivotal studies of tislelizumab. The flat dose of 200 mg administered intravenously (IV) once every 3 weeks is selected for further evaluation. The MTD has not been identified and only 1 DLT was reported in the first-in-human study.

Rates of treatment-related AEs and SAEs observed in patients receiving 2 mg/kg and 5 mg/kg (once every 2 weeks and once every 3 weeks) were comparable, suggesting no clear dose‑dependence across these regimens. Similarly, confirmed ORRs in patients who were treated with tislelizumab 2 mg/kg and 5 mg/kg once every 2 weeks ranged between 10% and 15%, compared to a range of 15% to 38% for patients treated at 2 mg/kg and 5 mg/kg once every 3 weeks.

According to PK data from BGB A317_Study_001, phase 1A, the CL of tislelizumab was found to be independent of body weight, ethnicity, and gender, and the observed serum exposure of a 200‑mg dose fell between serum exposure observed after 2 mg/kg and 5 mg/kg doses (dose range with comparable safety and efficacy rates).

Additionally, no unexpected treatment-related AEs occurred in 200 mg fixed-dose cohort (BGB‑A317_Study_001, phase 1A, part 3) when compared to body-weight-based cohorts. Of the evaluable patients treated (n = 13), 3 patients (23%) have achieved BOR of PR, 4 patients (31%) have achieved BOR of SD, and 6 patients (46%) have achieved BOR of PD. Therefore, clinical activity with a manageable and tolerable safety profile is expected to be maintained in patients receiving tislelizumab 200 mg once every 3 weeks.

Background Information on Ociperlimab As a TIGIT Inhibitor

- 1. Nonclinical Summary
     1. Pharmacology

Ociperlimab is a humanized immunoglobulin G (IgG) 1 monoclonal antibody against T-cell immunoglobulin and ITIM domain (TIGIT) under clinical development for the treatment of human malignancies.

Ociperlimab binds to the extracellular domain of human TIGIT with high specificity and affinity (equilibrium dissociation constant [K_D_] = 0.135 nM), as demonstrated by target binding assays and surface plasmon resonance (SPR) characterization. Ociperlimab has shown antitumor activities in both the GL261 mouse glioma tumor model and the CT26.WT mouse colon cancer model in humanized TIGIT knock-in mice. In the MC-38 mouse colon cancer model in humanized TIGIT knock-in mice, ociperlimab in combination with anti-mouse PD-1 significantly inhibited tumor growth compared with either therapy alone.

Ociperlimab has the constant region of a wild-type human IgG1 to enable the Fc-mediated effector functions. Ociperlimab has demonstrated competent binding to C1q and all FcγR and induces antibody-dependent cellular cytotoxicity against a TIGIT overexpressing cell line, but no antibody-dependent cellular cytotoxicity or complement-dependent cytotoxicity against primary T cells in the cell-based assays.

Refer to the Ociperlimab Investigator’s Brochure for detailed information on pharmacology studies.

- - 1. Toxicology

Humanized TIGIT knock-in mice containing human TIGIT gene and cynomolgus monkeys were selected for nonclinical safety evaluation of ociperlimab based on the homology of TIGIT amino acid sequence, binding affinity, and efficacy studies. Cynomolgus monkeys are the most relevant species.

Ociperlimab demonstrated a comparable binding affinity in TIGIT receptor occupancy assays with CD3+ splenocytes from humanized TIGIT knock-in mice compared to CD3+ human peripheral blood mononuclear cells (with EC_50_ of 48.8 ng/ml versus 63.2 ng/ml, respectively). In addition, ociperlimab showed a significant inhibition of GL261 tumor growth in humanized TIGIT knock-in mice at a dose of ≥ 0.4 mg/kg via weekly intraperitoneal dosing.

The toxicity and safety profile of ociperlimab was characterized in a 4-week repeated dose toxicology study in humanized TIGIT knock-in mice and a 13-week repeated dose toxicology study in cynomolgus monkeys. Ociperlimab was also evaluated in a 4-week repeated dose study in humanized TIGIT knock-in mice with subcutaneous MC-38 tumors. The cynomolgus monkey was considered the relevant species for toxicity studies based on the target sequence homology and cross-species TIGIT-binding activities of ociperlimab.

No apparent toxicity was noted in monkeys following repeated dosing at 10, 30, or 100 mg/kg once every 2 weeks for 13 weeks. The toxicokinetic profile in the monkey study showed that systemic exposure appeared to be dose proportional with no sex difference. No accumulation was observed over the 13-week dosing period in monkeys. No immunotoxicity was apparent as no changes in clinical pathology or histopathology were observed. Positive anti-drug antibodies (ADAs) against ociperlimab were observed in 6/10, 3/10, and 4/10 animals during the dosing period, and 3/4, 2/4, and 2/4 animals during the recovery period, at 10, 30, and 100 mg/kg, respectively. The anti-ociperlimab antibodies showed a rapid clearance of ociperlimab in serum in a few individual animals but did not appear to have an effect on the overall systemic exposure (area under the concentration-time curve [AUC]) or toxicity assessment.

No specific binding of ociperlimab was noted with normal human tissues. A variety of factors might contribute to the negative results, including negligible target expression in normal tissues and insufficient sensitivity of the immunohistochemistry method.

No significant increase in cytokine release was observed from an *in vitro* cytokine release assay following treatment of nonactivated peripheral blood mononuclear cells with ociperlimab when compared to human IgG. The results suggested that ociperlimab has potentially low risks of causing acute cytokine release syndrome.

Overall, no apparent toxicity was noted in the monkey toxicity study. No unexpected tissue cross reactivity was found in human or monkey tissues. The toxicokinetic profile showed dose proportional increases in systemic exposure without apparent drug accumulation or sex difference. Immunogenicity was observed without apparent immunotoxicity or effect on the systemic exposure. The NOAEL of ociperlimab was 100 mg/kg in the 13-week monkey toxicity study. The safety profile of ociperlimab is considered adequate to support first in human dosing.

Refer to the Ociperlimab Investigator’s Brochure for detailed information on toxicology studies.

- 1. Prior Clinical Experience With Ociperlimab

As of 16 June 2020, a phase 1 study (BGB-900-105) is investigating the safety/tolerability, pharmacokinetic (PK), and preliminary antitumor activity of ociperlimab in combination with tislelizumab with or without chemotherapy in patients with unresectable locally advanced or metastatic solid tumors.

Patients enrolled in study BGB-900-105 are treated with escalating doses of ociperlimab (50, 150, 450, or 900 mg) in combination with tislelizumab 200 mg once every 3 weeks; all patients cleared the dose-limiting toxicity (DLT) period without DLTs. The MTD was not reached and the RP2D has been determined to be 900 mg ociperlimab in combination with 200 mg tislelizumab once every 3 weeks. The most commonly reported TEAEs are fatigue (3 out of 11 patients), diarrhoea and aspartate aminotransferase (AST) increased (2 patients each out of 11 patients). Most TEAEs were Grade 1 or Grade 2, with the exception of three Grade 3 TEAEs of atrial flutter, pericardial effusion malignant and dyspnea, which were serious and deemed not related to study treatments (1 patient each).

Treatment-related TEAEs (ociperlimab, tislelizumab, or both) occurred in 3 patients and include influenza, AST increased, and dry skin in 1 patient; abdominal pain, dry eye, and diarrhea in 1 patient; and fatigue in 1 patient. All treatment-related TEAEs were Grade 1. No infusion reactions have been reported.

The study is currently enrolling patients at the RP2D. Ociperlimab continues to appear to be safe and well-tolerated. Preliminary results also showed that 100% receptor occupancy was achieved at 50 mg.

Refer to the Ociperlimab Investigator’s Brochure for detailed information on clinical experience.

Table 6: Treatment-Emergent Adverse Events by System Organ Class, Preferred Term, and Maximum Severity (Safety Analysis Set)

|  | Tislelizumab 200 mg | | | |  |
| --- | --- | --- | --- | --- | --- |
| **System Organ Class**  **Preferred term** | Ociperlimab 50 mg +  (N = 1) n (%) | Ociperlimab 150 mg  (N = 3) n (%) | Ociperlimab 450 mg  (N = 4) n (%) | Ociperlimab 900 mg  (N = 3) n (%) | Total  (N=11) n (%) |
| **Patients with ≥ 1 TEAE(s)** | **1 (100.0)** | **3 (100.0)** | **3 (75.0)** | **1 (33.3)** | **8 (72.7)** |
| **Gastrointestinal disorders** | **1 (100.0)** | **1 (33.3)** | **2 (50.0)** | **0 (0.0)** | **4 (36.4)** |
| Diarrhea | 0 (0.0) | 1 (33.3) | 1 (25.0) | 0 (0.0) | 2 (18.2) |
| Abdominal pain | 0 (0.0) | 0 (0.0) | 1 (25.0) | 0 (0.0) | 1 (9.1) |
| Constipation | 0 (0.0) | 0 (0.0) | 1 (25.0) | 0 (0.0) | 1 (9.1) |
| Dry mouth | 1 (100.0) | 0 (0.0) | 0 (0.0) | 0 (0.0) | 1 (9.1) |
| **General disorders and administration site conditions** | **1 (100.0)** | **0 (0.0)** | **1 (25.0)** | **1 (33.3)** | **3 (27.3)** |
| Fatigue | 1 (100.0) | 0 (0.0) | 1 (25.0) | 1 (33.3) | 3 (27.3) |
| **Musculoskeletal and connective tissue disorders** | **0 (0.0)** | **1 (33.3)** | **1 (25.0)** | **1 (33.3)** | **3 (27.3)** |
| Back pain | 0 (0.0) | 1 (33.3) | 0 (0.0) | 0 (0.0) | 1 (9.1) |
| Lumbar pain | 0 (0.0) | 0 (0.0) | 0 (0.0) | 1 (33.3) | 1 (9.1) |
| Groin pain | 0 (0.0) | 0 (0.0) | 1 (25.0) | 0 (0.0) | 1 (9.1) |
| **Infections and infestations** | **1 (100.0)** | **0 (0.0)** | **1 (25.0)** | **0 (0.0)** | **2 (18.2)** |
| Influenza | 1 (100.0) | 0 (0.0) | 0 (0.0) | 0 (0.0) | 1 (9.1) |
| Otitis externa | 0 (0.0) | 0 (0.0) | 1 (25.0) | 0 (0.0) | 1 (9.1) |
| **Investigations** | **1 (100.0)** | **1 (33.3)** | **0 (0.0)** | **0 (0.0)** | **2 (18.2)** |
| Aspartate aminotransferase increased | 1 (100.0) | 1 (33.3) | 0 (0.0) | 0 (0.0) | 2 (18.2) |
| **Nervous system disorders** | **1 (100.0)** | **0 (0.0)** | **1 (25.0)** | **0 (0.0)** | **2 (18.2)** |
| Neuralgia | 1 (100.0) | 0 (0.0) | 0 (0.0) | 0 (0.0) | 1 (9.1) |
| Somnolence | 0 (0.0) | 0 (0.0) | 1 (25.0) | 0 (0.0) | 1 (9.1) |
| **Respiratory,thoracic and mediastinal disorders** | **0 (0.0)** | **1 (33.3)** | **1 (25.0)** | **0 (0.0)** | **2 (18.2)** |
| Cough | 0 (0.0) | 0 (0.0) | 1 (25.0) | 0 (0.0) | 1 (9.1) |
| Dyspnoea | 0 (0.0) | 1 (33.3) | 0 (0.0) | 0 (0.0) | 1 (9.1) |
| **Skin and subcutaneous tissue disorders** | **1 (100.0)** | **0 (0.0)** | **0 (0.0)** | **1 (33.3)** | **2 (18.2)** |
| Drug eruption | 0 (0.0) | 0 (0.0) | 0 (0.0) | 1 (33.3) | 1 (9.1) |
| Dry skin | 1 (100.0) | 0 (0.0) | 0 (0.0) | 0 (0.0) | 1 (9.1) |
| **Blood and lymphatic system disorders** | **0 (0.0)** | **0 (0.0)** | **1 (25.0)** | **0 (0.0)** | **1 (9.1)** |
| Anemia | 0 (0.0) | 0 (0.0) | 1 (25.0) | 0 (0.0) | 1 (9.1) |
| **Cardiac disorder** | **1 (100.0)** | **0 (0.0)** | **0 (0.0)** | **0 (0.0)** | **1 (9.1)** |
| Atrial flutter | 1 (100.0) | 0 (0.0) | 0 (0.0) | 0 (0.0) | 1 (9.1) |
| **Ear and labyrinth disorders** | **0 (0.0)** | **1 (33.3)** | **0 (0.0)** | **0 (0.0)** | **1 (9.1)** |
| Ear discomfort | 0 (0.0) | 1 (33.3) | 0 (0.0) | 0 (0.0) | 1 (9.1) |
| **Eye disorder** | **0 (0.0)** | **0 (0.0)** | **1 (25.0)** | **0 (0.0)** | **1 (9.1)** |
| Dry eye | 0 (0.0) | 0 (0.0) | 1 (25.0) | 0 (0.0) | 1 (9.1) |
| **Neoplasms benign, malignant and unspecified (incl cysts and polyps)** | **0 (0.0)** | **0 (0.0)** | **1 (25.0)** | **0 (0.0)** | **1 (9.1)** |
| Cancer pain | 0 (0.0) | 0 (0.0) | 1 (25.0) | 0 (0.0) | 1 (9.1) |
| Pericardial effusion malignant | 0 (0.0) | 0 (0.0) | 1 (25.0) | 0 (0.0) | 1 (9.1) |
| **Psychiatric disorders** | **0 (0.0)** | **1 (33.3)** | **0 (0.0)** | **0 (0.0)** | **1 (9.1)** |
| Confusional state | 0 (0.0) | 1 (33.3) | 0 (0.0) | 0 (0.0) | 1 (9.1) |

- 1. Clinical Pharmacology of Ociperlimab

As of 16 June 2020, preliminary PK data of ociperlimab are available from a total of 11 patients treated with ociperlimab at 50 mg (n = 1), 150 mg (n = 3), 450 mg (n = 4), and 900 mg (n = 3) dose levels in combination with tislelizumab 200 mg in the dose-escalation portion of study BGB-900-105. Ociperlimab serum concentrations declined in a bi-exponential manner after intravenous infusion and the ociperlimab exposures (C_max_ and AUC) increased approximately dose-proportionally from 50 mg to 900 mg (Figure 1).

Peripheral TIGIT receptor occupancy data are available for 11 enrolled patients treated with ociperlimab at the 50 mg (n = 1), 150 mg (n = 3), 450 mg (n = 4), and 900 mg (n = 3) dose levels in study BGB-900-105. Complete (100%) TIGIT receptor occupancy has been observed on CD8, CD4, NK, and Treg cells in peripheral blood at all the tested dose levels.

Figure 1: Cycle 1 Mean (± SD) Serum Concentration-Time Profiles of Ociperlimab in Study BGB-900-105


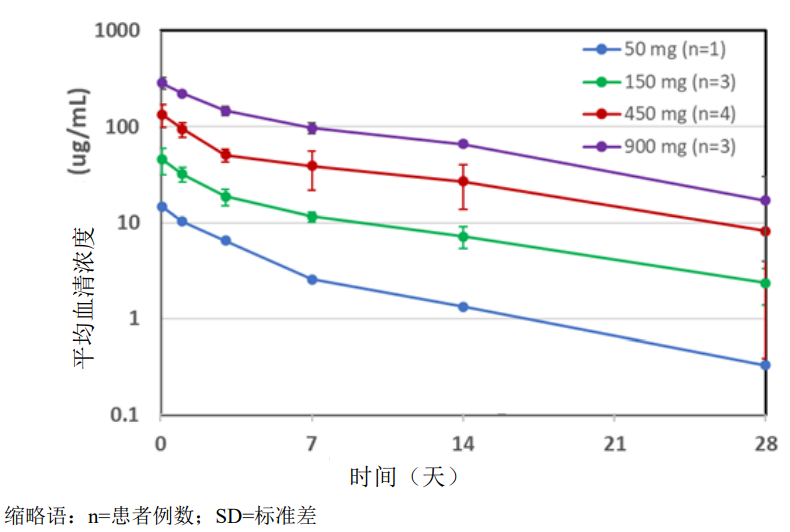


Mean Serum Concentration (μg/mL)

Abbreviations: n, patient number; SD, standard deviation

Time (Day)

Study Rationale

BTC is a highly malignant cancer, with poor prognosis and increasing incidence. It often presents insidiously, spreads easily, and is frequently diagnosed at an advanced stage, with only 10% of patients being candidates for curative surgical resection. For most patients with advanced BTC, systemic therapy has become the mainstay of treatment. However, options for first-line treatment are limited in both availability and efficacy, thus there is an urgent need to develop new modalities to improve overall survival for these patients.

- 1. Rationale forSynergistic Effect of Ociperlimab plus Tislelizumab

Ociperlimab plus an anti-PD-1 inhibitor showed antitumor effect in *in vitro* mouse tumor models

The antitumor activity for the combination of ociperlimab with anti-mouse PD‑1 1Ch15mt was investigated in the MC38 mouse colon cancer model in humanized TIGIT knock-in mice. The combination of ociperlimab and Ch15mt showed higher TGI (102%) than either antibody alone (73% for Ch15mt alone or 11% for ociperlimab alone). This *in vitro* study suggested that the PD-1 inhibitor tislelizumab may have synergistic antitumor effect with ociperlimab**^45^**.

A joint analysis of clinical specimens from patients with ICC showed that high expression of TIGIT and PD-L1 may indicate a shorter survival time

A previous study involving 297 patients with ICC showed that TIGIT expression levels were significantly elevated in tumor tissues, which was associated with a higher recurrence rate and shorter survival times. This indicates that TIGIT expression can predict prognosis and serves as a therapeutic target for ICC. Joint analysis of TIGIT and PD-L1 expression revealed that patients with high expression of both biomarkers had significantly worse prognoses compared to those with high expression of only one marker or low expression of both markers, suggesting the significant value of combining anti-PD-L1 and anti-TIGIT antibodies**^46^**.

Several clinical trials investigating the efficacy of ociperlimab in combination with tislelizumab in solid tumors are ongoing

Study BGB-900-105 is investigating the safety/tolerability, PK, and preliminary antitumor activity of ociperlimab in combination with tislelizumab with or without chemotherapy in patients with unresectable locally advanced or metastatic solid tumors. Preliminary results have confirmed that the combination of tislelizumab and ociperlimab was well tolerated without DLT, and the recommended phase 2 dose had been established**^45^**. Tislelizumab in combination with ociperlimab is being investigated in various tumor types such as lung cancer (NCT04746924, NCT04866017), cervical cancer (NCT04693234), and esophageal cancer (NCT04732494).

- 1. Rationale for Combination of Gemcitabine/Cisplatin (GP) with Immunotherapy

Gemcitabine/cisplatin is currently recognized as the "gold standard" for the first-line treatment of advanced BTC, but its efficacy remains unsatisfactory. Increasing clinical evidence suggests that GP-based combination therapies may be more effective.

Gemcitabine, a nucleoside analogue commonly used in the treatment of pancreatic, lung squamous cell carcinoma, and other cancers, reduces the number of circulating myeloid-derived suppressor cells (MDSCs) and facilitates the transition of tumor-associated macrophages (TAMs) to an immunostimulatory phenotype**^47^**. In addition to its direct immunostimulatory effects, gemcitabine stimulates tumor cells to express MHC class I molecules, thereby increasing their antigenicity**^48^**.

Cisplatin, a commonly used cytotoxic chemotherapeutic agent, has been reported to enhance immune activity in a dose-dependent manner by reducing PD-L2 expression on human dendritic and tumor cells, leading to antigen-specific proliferation and promotion of Th1 cytokine secretion, and enhanced recognition of tumor cells by T cells**^49^**. Cisplatin in combination with gemcitabine has been shown to significantly reduce the percentage of regulatory T cells (Tregs), thereby enhancing immune activity**^50^**.

- 1. Rationale for Biomarkers

Cholangiocarcinoma is characterized by insidious onset and is often asymptomatic at early stage, lacking specific biochemical diagnostic indicators. With the recent advancements in molecular diagnostics techniques, an increasing number of clinical studies are exploring biomarkers associated with the efficacy and prognosis of cholangiocarcinoma.

A study investigating known biomarkers of cholangiocarcinoma revealed that tumors with positive PD-L1 expression had a higher tumor mutation burden compared with tumors without PD-L1 expression. At the same time, mutations in BRAF, BRCA2, RNF43, and TP53 were also significantly increased**^51^**.

Preliminary researches of this IIT study have shown that ICC patients with high expression of both PD-L1 and TIGIT had significantly worse prognoses compared to those with high expression of only one marker or low expression of both markers**^46^**. Therefore, exploring PD-L1 and TIGIT expression would be valuable for developing personalized therapies and combination treatments for cholangiocarcinoma, as well as for developing predictive companion diagnostics for drug efficacy or prognosis.

In summary, the proposed study aims to explore the efficacy and safety of adding tislelizumab (anti-PD-1 inhibitor) and ociperlimab (anti-TIGIT inhibitor) to standard first-line chemotherapy for advanced BTC, providing new treatment options and extending treatment benefit to larger population, which holds significant clinical value.

Study Objectives and Endpoints

- 1. Study Objectives
     1. Primary Objective
- To assess efficacy of GP in combination with tislelizumab and ociperlimab as first-line treatment for unresectable advanced BTC by objective response rate (ORR) as assessed by the investigator per Response Evaluation Criteria in Solid Tumors (RECIST) version1.1.
  - 1. Secondary Objectives
- To assess safety and tolerability of GP in combination with tislelizumab and ociperlimab as first-line treatment for unresectable advanced BTC
- To assess efficacy of GP in combination with tislelizumab and ociperlimab as first-line treatment for unresectable advanced BTC by disease control rate (DCR) as assessed by the investigator.
- To assess efficacy of GP in combination with tislelizumab and ociperlimab as first-line treatment for unresectable advanced BTC by duration of response (DoR) as assessed by the investigator.
- To assess efficacy of GP in combination with tislelizumab and ociperlimab as first-line treatment for unresectable advanced BTC by progression-free survival (PFS) and PFS rates at 6 months and at 12 months as assessed by the investigator.
- To assess efficacy of GP in combination with tislelizumab and ociperlimab as first-line treatment for unresectable advanced BTC by overall survival (OS) and OS rates at 6 months and at 12 months as assessed by the investigator.
  - 1. Exploratory Objective
- To explore potential biomarkers that may correlate with clinical efficacy/prognosis of GP in combination with tislelizumab and ociperlimab as first-line treatment for unresectable advanced BTC.
  1. Study Endpoints
     1. Primary Endpoint
- ORR assessed by the investigator per RECIST v1.1: the proportion of patients who have achieved complete response (CR) and/or partial response (PR)
  - 1. Secondary Endpoints
- DCR assessed by the investigator per RECIST v1.1: the proportion of patients whose DCR is CR, PR, and stable disease (SD).
- DoR assessed by the investigator per RECIST v1.1: the time from the date that the response criteria (CR or PR) are first met until the confirmation of disease progression (PD) or death, whichever occurs first
- PFS assessed by the investigator: the time from the beginning of study treatment to the date of disease progression as assessed per RECIST v1.1 or death from any cause, whichever occurs first
- PFS rates assessed by the investigator: the proportion of patients remain alive and progression-free as assessed per RECIST v1.1 at the 6 and 12 months from the beginning of study treatment.
- OS assessed by the investigator: the time from the beginning of study treatment until the date of death.
- OS rates assessed by the investigator: the proportion of patients remain alive at the 6 and 12 months from the beginning of study treatment.
- Incidence, nature, and severity of adverse events, serious adverse events per the National Cancer Institute-Common Terminology Criteria for Adverse Events (NCI‑CTCAE) Version 5.0: all these parameters will be determined based on NCI-CTCAE v5.0. Safety and tolerability will be assessed in combination with clinical laboratory abnormalities, relevant physical examinations, electrocardiograms (ECGs) and vital signs.
  - 1. Exploratory Endpoint
- Correlation between expression levels of potential biomarkers programmed cell death ligand-1 (PD-L1) and T-cell immunoglobulin and ITIM domain (TIGIT) with disease status, response to/prognosis of GP in combination with tislelizumab and ociperlimab as first-line treatment for unresectable advanced BTC

Study Design

- 1. Summary of Study Design

This is an open-label, multicenter, single-arm phase 2 study designed to evaluate the efficacy and safety of GP in combination with tislelizumab and ociperlimab as first-line treatment for unresectable advanced BTC. 45 patients are planned to be enrolled in the study. The study aims to explore the correlation between expression levels of potential biomarkers PD-L1 and TIGIT with disease status, treatment response/prognosis.

The primary endpoint of the study is ORR as assessed by the investigator per RECIST v1.1.

Study Schema


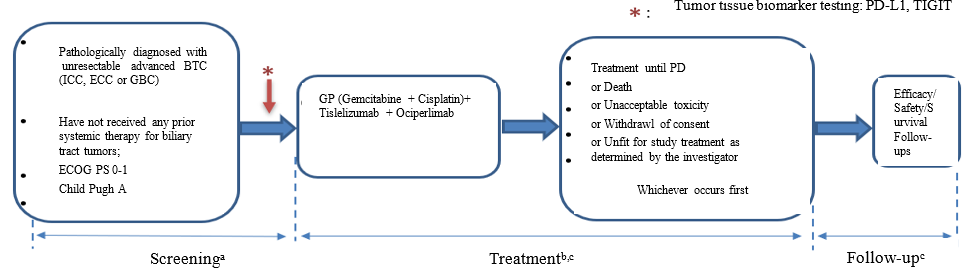


- 1. Archival tumor tissue samples or fresh biopsies must be available for biomarker testings
  2. Tislelizumab and ociperlimab will be administered intravenously on Day 1 of each 21-day cycle (once every 3 weeks). Tislelizumab (200 mg) and ociperlimab (900 mg) will be administered at flat doses.
  3. Gemcitabine 1000 mg/m^2^ + cisplatin 25 mg/m^2^ regimen will be administered on the first and eighth day of each cycle for up to 8 cycles, with chemotherapy cycles and dosage being adjusted based on tolerability. Patients who are intolerant to chemotherapy or have achieved stable disease or objective response after completing 8 cycles of chemotherapy will continue the treatment of tislelizumab 200 mg plus ociperlimab 900 mg until disease progression, unacceptable toxicity, death, withdrawal of consent, or other conditions that make the patient unsuitable for the study, as determined by the investigator.
  4. Survival follow-up should continue until initiation of subsequent anticancer therapy, the patient withdraws consent, dies, is lost to follow-up or the study terminates, whichever occurs first.
  5. Screening Period

Screening evaluations will be performed within 28 days before the first dose of study treatment(s). After providing written informed consent, patients will complete all the screening assessments before the first dose of study treatment to confirm their eligibility for study participation.

Tumor imaging assessments must be performed within 28 days before the first dose of study treatment(s).

- 1. Treatment Period

After completing all screening activities, patients who are confirmed to be eligible by the investigator will be enrolled to receive GP regimen (gemcitabine + cisplatin) in combination with tislelizumab and ociperlimab.


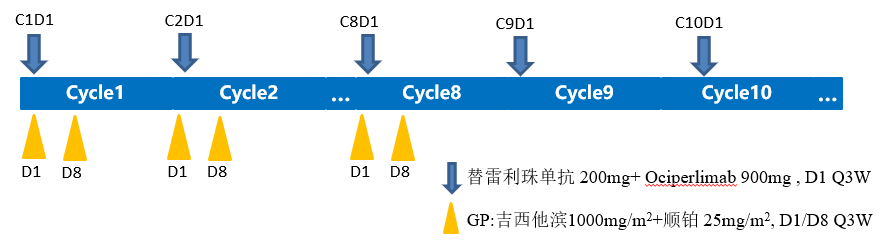


Tislelizumab 200 mg + Ociperlimab 900 mg, D1 Q3W

GP: Gemcitabine 1000 mg/m^2^ + Cisplain 25 mg/m^2^, D1/D8 Q3W

Patients will receive tislelizumab at the dose of 200 mg intravenously followed by the administration of ociperlimab at the dose of 900 mg on Day 1 of each 21-day cycle (i.e, once every 3 weeks).

Gemcitabine 1000 mg/m^2^ + cisplatin 25 mg/m^2^ regimen will be administered on the first and eighth day of each cycle for up to 8 cycles, with chemotherapy cycles and dosage being adjusted based on tolerability.

Relevant laboratory tests should be performed within 48 hours before administration in each cycle. Refer to Appendix 1 for relevant tests.

Patients who are intolerant to chemotherapy or have achieved stable disease or objective response after completing 8 cycles of chemotherapy will continue the treatment of tislelizumab 200 mg plus ociperlimab 900 mg until one of the following occurs (whichever occurs first):

1) Disease progression;

2) Unacceptable toxicity;

3) Death;

4) Patient’s withdrawal of consent;

5) Study termination;

6) Patient’s loss to follow-up;

7) Any discontinuation criteria in the protocol met

Patients who discontinue study treatment for reasons other than disease progression (e.g, toxicity) will continue to undergo efficacy follow-up visits until the patients withdraw consent, die, are lost to follow-up or the study terminates, whichever occurs first.

Safety will be assessed throughout the study by monitoring AEs/SAEs (toxicity grades assigned per National Cancer Institute Common Terminology Criteria for Adverse Events [NCI-CTCAE v5.0] and laboratory abnormalities. Vital signs, physical examinations, ECOG performance status change as well as ECG findings will also be used for safety assessment.

- 1. End-of-Treatment Visit and Safety Follow-up Telephone Visit

Patients who discontinue from study treatment for any reason will be asked to return to the clinic for the EOT Visit, which is required to be conducted within 30 days after the last dose of study treatment(s) or before the initiation of a new anticancer treatment, whichever occurs first. If routine laboratory tests (e.g, hematology, clinical chemistry) are completed ≤ 7 days before the EOT Visit, these tests do not need to be repeated. Tumor assessment is not required at the EOT Visit if ≤ 9 weeks have passed since the last assessment. If the study treatment(s) were initially interrupted and then permanently discontinued due to AE(s), the EOT Visit may occur later, but not later than the permitted time of dose delay plus 7 days.

Telephone contacts with patients should be conducted to assess irAE and concomitant medications (if appropriate, i.e, associated with an irAE or is a new anticancer therapy) at 60 days (± 14 days) and 90 days (± 14 days) after the last dose of tislelizumab and ociperlimab, respectively, regardless of whether or not the patient starts a new anticancer therapy. If a patient reports a suspected irAE at a telephone follow-up contact, the investigator should arrange an unscheduled visit if further assessment is indicated.

All AE, including SAEs, will be collected as described in **Section 11.3**. All drug-related SAEs will be recorded by the investigator after treatment discontinuation until patient death or loss to follow-up, whichever occurs first.

The EOT Visit at which a response assessment showed progressive disease, resulting in patient discontinuation, can be used as the Safety Follow-up Visit, if it occurred 30 days (± 7 days) after the last dose of study treatment.

- 1. Survival Follow-up Visit

Patients who discontinue study treatment(s) for reasons other than disease progression (e.g, toxicity) will continue to undergo assessments according to the Schedule of Assessments ([**Appendix 1**](#APPX1)), until the patients withdraw consent, loss to follow-up, die or until the study terminates, whichever occurs first.

Patients will be followed for survival and to obtain information on subsequent anticancer therapy information after discontinuation of study treatment via telephone calls, patient medical records, and/or clinic visits approximately every 3 months (± 14 days) after the EOT Visit until death, loss to follow-up, withdrawal of consent, or study termination.

- 1. Discontinuation of Study Treatment/Study

Patients who discontinue study treatment but have not withdrawn consent for follow-up should continue to be followed for assessments, if possible.

- - 1. Discontinuation of Study Treatment

Patients have the right to voluntarily withdraw from the study or discontinue study treatment at any time for any reason. In addition, the investigator has the right to withdraw a patient from the study treatment at any time. Patients may discontinue study treatment for reasons including but not limited to the following:

- Disease progression as assessed by the investigator
- Unacceptable toxicity related to study treatment, including development of an immune‑related adverse event determined by the investigator to be unacceptable given the individual patient’s potential response to therapy and severity of the event
- Any medical condition that the investigator determines may jeopardize the patient’s safety if he or she were to continue the study treatment
- Use of any concurrent non-protocol antineoplastic therapy (i.e. chemotherapy, hormonal therapy, immunotherapy for the treatment of cancer, or radiotherapy or standard/investigational agents [including Chinese herbal medicine and Chinese patent medicines] for the treatment of liver cancer)
- Pregnancy
- Patient noncompliance to study treatment
- Patients will no longer benefit from continued treatment as judged by the investigator

The primary reason for study treatment discontinuation should be documented on the patient's chart and appropriate eCRF.

- - 1. Patient Discontinuation From Study (End of Study for an Individual Patient)

Patients may discontinue from the study for reasons that include, but are not limited to, the following:

- Patient withdrawal of consent
- Death
- Loss to follow-up
- Patient completion of all study assessments
  - 1. Study Termination and Study Site Closure

The end of study is defined as the time point when the final data for a clinical study are collected, which is after the last study patient has made the final visit to the study location, and **this is expected to occur approximately 2 years after the enrollment of the last patient**.

Safety and efficacy analyses will be conducted upon the observation of a predefined number of events. The study will continue until the last patient's death, loss to follow-up, or withdrawal from the study or until study termination by sponsor.

The sponsor has the right to terminate this study at any time. Reasons for terminating the study prematurely include, but are not limited to, the following:

- The incidence or severity of AEs in this or other studies indicates a potential health hazard to patients
- Overall patient enrollment is slow

The sponsor will notify each investigator if a decision is made to terminate the study. Should this be necessary, prematurely discontinued patients should be seen as soon as possible for an EOT Visit and EOT assessment.

The investigators may be informed of additional procedures to be followed to ensure that adequate consideration is given to the protection of the patient’s interests. The investigator will be responsible for informing Institutional Review Boards (IRBs)/Independent Ethics Committees (IECs) of the early termination of the study.

The sponsor has the right to close a site at any time. The decision will be communicated to the site in advance. Reasons for closing a site may include but are not limited to the following:

- Patient enrollment is too slow
- Poor protocol compliance
- Inaccurate, delayed or incomplete data recording
- Good Clinical Practice (GCP) noncompliance
- Study activity is completed (i.e, all patients have completed and all obligations have been fulfilled)

Study Population

The investigator must keep records (e.g., patient screening logs) of patients who have entered the study for screening. This information is to indicate that there will be no deviations during the selection of patients.

- 1. Inclusion Criteria

Patients are eligible for inclusion in the study if they meet the following criteria:

1. Male or female, aged 18 to 75 years old;

2. Patients must be able to understand and willing to sign the written informed consent form (ICF) and must sign the ICF before enrollment;

3. Patients with histologically or cytologically confirmed unresectable advanced BTC (including ICC, ECC, and GBC), and agree to submit archival tumor tissue samples or fresh tumor biopsies for biomarker testing;

4. Have least 1 measurable lesion as defined by RECIST v1.1;

5. Have not received any prior systemic therapy for biliary tract tumors, including chemotherapy and immunotherapy;

6. Eastern Cooperative Oncology Group (ECOG) Performance Status (PS) score 0 to 1;

7. Child-Pugh Class A classification for liver function;

8. Patients must have adequate bone marrow, liver and kidney function as indicated by the following laboratory assessment criteria (obtained ≤ 7 days before the first dose of study treatment):

Hematology:

Absolute neutrophil count (ANC) ≥ 1.5 x 10^9^/L, platelets ≥ 100 x 10^9^/L, and hemoglobin ≥ 90 g/L

Hepatic function:

AST and ALT ≤ 3 x upper limit of normal value (ULN); total bilirubin ≤ 1.5 x ULN

Patients with obstructive jaundice can be considered for enrollment if their hepatic function meet the inclusion criteria following the treatment with percutaneous transhepatic cholangial drainage (PTCD) or endoscopic retrograde cholangiopancreatography (ERCP)

Renal function:

Serum creatinine clearance (CrCl) ≥ 45 mL/min

Coagulation:

International normalized ratio (INR) ≤ 1.5

9. Patients with HBV or HCV infection must meet the following criteria:

For patients with inactive/asymptomatic HBV carrier, chronic, or active HBV:

Must have HBV DNA < 2000 copies/mL at Screening.

Note: Patients with detectable hepatitis B surface antigen (HBsAg) or detectable HBV DNA should be managed per treatment guidelines. Patients receiving antiviral treatments at Screening should have HBV DNA < 2000 copies/mL and will continue the antiviral treatment during the study.

For patients with HCV infection:

Detectable HCV RNA level is indicative of infection and patients with detectable HCV RNA are not eligible to participate in this study.

10. Females of childbearing potential (i.e., have the physiological ability to become pregnant) must be willing to use a highly effective method of birth control for the duration of the study and for 120 days after the last dose of study treatment, and have had a negative urine or serum pregnancy test within 7 days of the first dose of study treatment;

11. Non-sterile males must be willing to use a highly effective method of birth control for the duration of the study and for 120 days after the last dose of study treatment;

12. Life expectancy ≥ 3 months.

- 1. Exclusion Criteria

Patients are not eligible to participate in this study if they meet any of the following exclusion criteria:

1. Diagnosis of ampullary cancer, mixed hepatocellular and cholangiocellular carcinoma;

2. History of severe hypersensitivity reactions to other monoclonal antibodies;

3. Allergy to tislelizumab, ociperlimab (anti-TIGIT inhibitor) or any of its excipients; allergy to cisplatin and any of its excipients; allergy to gemcitabine and any excipients;

4. Pericardial effusion, uncontrollable pleural effusion, or clinically significant ascites within 7 days before the first dose of study treatment, defined as meeting the following criteria: (a) ascites can be detected during physical examination at Screening, and (b) ascites that requires abdominal tapping for drainage at Screening;

5. Clinical evidence of portal hypertension with esophageal or gastric varices within 6 months before the first dose of study treatment;

6. Bleeding or thrombotic disorders or use of anticoagulants, such as warfarin or similar agents, requiring therapeutic international normalized ratio (INR) monitoring within 6 months before the first dose of study treatment;

7. Prior malignancies except for the BTC under investigation in this study and any locally recurring cancer that has been treated curatively (e.g, resected basal or squamous cell skin cancer, superficial bladder cancer, carcinoma *in situ* of the cervix or breast);

8. Any known central nervous system metastasis and/or leptomeningeal disease before the first dose of study treatment;

9. Active immunodeficiency or autoimmune disease(s) at Screening and/or history of immunodeficiency or autoimmune disease(s) that may relapse at Screening;

Note: patients with the following conditions will not be excluded:

• Type 1 diabetes mellitus that is clinically manageable

• Hypothyroidism that is controlled by hormonal replacement therapy

• Controlled celiac disease (≤ 3 times/day)

• Skin diseases not requiring systemic treatment (e.g, vitiligo, psoriasis, or alopecia)

• Any other disease that is not expected to recur in the absence of external triggering factors

10. Any condition that requires systemic treatment with either corticosteroids (dose > 10 mg daily of prednisone or equivalent) or other immunosuppressive agents ≤ 14 days before the first dose of study treatment;

Note: Patients who are currently or had previously been on any of the following steroid regimens will not be excluded:

• Adrenal replacement steroid (dose ≤ 10 mg daily of prednisone or equivalent) are permitted in the absence of active autoimmune disease

• Topical, ocular, intra-articular, intranasal, or inhalational corticosteroid with minimal systemic absorption

• Short course (≤ 7 days) of corticosteroid prescribed prophylactically (e.g, for contrast dye allergy) or for the treatment of a non-autoimmune condition (eg, delayed-type hypersensitivity reaction caused by contact allergen)

11. With history of interstitial lung disease or non-infectious pneumonitis;

12. Severe chronic or active infection (including tuberculosis, excluding viral hepatitis) requiring systemic antibacterial, antifungal, or antiviral therapy before the first dose of study treatment;

13. Screening ECG shows QT corrected (QTc) interval (corrected by Fridericia’s method) > 450 msec;

Note: An ECG will be repeated to confirm any reported QTc interval ＞ 450 msec from the initial testing.

14. Any of the following cardiovascular risk factors:

- Cardiac chest pain, defined as moderate pain that limits instrumental activities of daily living (ADL), within 28 days before the first dose of study treatment;
- Symptomatic pulmonary embolism within 28 days before the first dose of study treatment;
- Any history of acute myocardial infarction within 6 months before the first dose of study treatment;
- Any history of heart failure meeting New York Heart Association Classification III or IV within 6 months before the first dose of study treatment;
- Any event of ventricular arrhythmia ≥ Grade 2 in severity within 6 months before the first dose of study treatment;
- Cerebral vascular accident (CVA) or transient ischemic attack (TIA) within 6 months before the first dose of study treatment;

15. Received organ transplantation or hematopoietic stem cell transplantation (HSCT) or any major surgical procedure within 28 days before the first dose of study treatment;

16. Known mental disorders or substance abuses that may compromise the study compliance;

17. Was administered a live vaccine within 28 days before the first dose of study treatment; Note: Seasonal vaccines for influenza are generally inactivated vaccines and are allowed.

18. Known history of human immunodeficiency virus (HIV) infection or syphilis infection;

19. Currently participating in other studies and receiving treatments, or participating in other clinical trials of drug or medical device within 4 weeks after the first dose of study treatment;

20. Pregnant or lactating women, become pregnant or lactating from the Screening visit to 120 days after the last dose of the study treatment, or prepare for pregnancy or give birth to a child within the planned study duration;

21. Poor study compliance as determined by the investigator, or other conditions that render the patient ineligible for the study;

22. Medical contraindications that preclude the use of contrast-enhanced imaging (CT or MRI).

Study Treatment(s)

- 1. Formulation, Packaging and Handling
     1. Tislelizumab

Tislelizumab is a monoclonal antibody formulated for intravenous infusion in a single-use vial (20R glass, United States Pharmacopeia [USP] type I), containing a total of 100 mg antibody in 10 mL of isotonic solution as available. Tislelizumab has been aseptically filled in a single-use vial with a Flurotec-coated butyl rubber stopper and an aluminum cap. Subsequently, vials are packaged in carton boxes.

The contents of the label will be in accordance with all applicable local regulatory requirements.

Tislelizumab must be kept at the temperature condition as specified on the label. Shaking should be avoided.

Refer to the Pharmacy Manual for details regarding intravenous administration, accountability, and disposal. Please refer to the Tislelizumab Investigator’s Brochure for more information.

- - 1. Ociperlimab

Ociperlimab is a monoclonal antibody formulated for intravenous infusion in a single-use vial (20 mL glass vial, USP Type I) containing a total of 200 mg antibody in 10 mL (or 300 mg antibody in 15 mL) of buffered isotonic solution as available. Ociperlimab has been aseptically filled in a single-use vial with a FluroTec-coated butyl rubber stopper and an aluminum cap. Each vial is packaged into a single-carton box.

The contents of the label will be in accordance with all applicable local regulatory requirements.

Ociperlimab must be kept at the temperature condition as specified on the label. Shaking should be avoided.

Refer to the Pharmacy Manual for details regarding intravenous administration, accountability, and disposal. Please refer to the Ociperlimab Investigator’s Brochure for more information.

Investigational products must be dispensed or administered according to procedures described herein. Only patients enrolled in the study may receive study treatment(s) in accordance with all applicable regulatory requirements. Only authorized study site personnel may supply or administer study treatment(s).

- - 1. Gemcitabine and Cisplatin

The actual appearance and composition of gemcitabine and cisplatin may vary depending on the manufacturers.

The contents of the label will be in accordance with all applicable local regulatory requirements.

Gemcitabine and cisplatin must be kept at the temperature condition as specified on their respective labels.

For further details, please refer to the manufacturer’s prescribing information.

- 1. Dosage, Administration, and Compliance
     1. Treatment Administration

Dosing schedules for tislelizumab, ociperlimab and GP regimen are presented separately in Table 7. All patients will be monitored continuously for AEs. Treatment modifications (e.g, dose delay, interruption, reduction, ordiscontinuation) will be based on specific laboratory and AE criteria, **as described in Section 11.3**.

Table 7: Dosage and Timing of Dose for Each Patient

| **Treatment/drug** | **Dosage** | **Frequency and sequence of administration** | **Route of administration** | **Duration of treatment** |
| --- | --- | --- | --- | --- |
| Tislelizumab | 200 mg | Day 1 of each cycle (each 21-day cycle), administration at first | Intravenously | Refer to Section 6.3 |
| Ociperlimab | 900 mg | Day 1 of each cycle (each 21-day cycle), administration after tislelizumab | Intravenously | Refer to Section 6.3 |
| GP regimen | Gemcitabine 1000 mg/m^2^ and cisplatin 25 mg/m^2^ | Day 1 and Day 8 of each cycle | Intravenously | Refer to Section 6.3 |

Table 8: Study Treatment Administration and Monitoring Time

|  |  |
| --- | --- |
| C1D1 and C2D1 | Tislelizumab infusion over 60 minutes followed by ociperlimab infusion over 60 minutes;  GP regimen: gemcitabine by intravenous drip for 30 minutes + cisplatin by intravenous drip (refer to relevant prescribing information)  Patient monitoring for ≥ 120 minutes |
| C3D1 to C8D1 | Tislelizumab infusion over 30 minutes followed by ociperlimab infusion over 30 minutes;  GP regimen: gemcitabine by intravenous drip for 30 minutes + cisplatin by intravenous drip (refer to relevant prescribing information)  Patient monitoring for ≥ 60 minutes |
| C9D1 onwards | Tislelizumab infusion over 30 minutes followed by ociperlimab infusion over 30 minutes  Patient monitoring for ≥ 60 minutes |

Abbreviations: C1D1, Cycle 1 Day 1; C2D1, Cycle 2 Day 1; C3D1, Cycle 3 Day 1.

All drugs will be administered by intravenous infusion through an intravenous line containing a sterile, non-pyrogenic, low-protein-binding 0.2 or 0.22 μm in-line or add-on filter. Specific instructions for product preparation and administration are provided in the Pharmacy Manual.

The initial infusion (Day 1 of Cycle 1 and Cycle 2) will be delivered over 60 minutes; if this is well tolerated, then the subsequent infusions will be administered over 30 minutes, which is the shortest time period permissible for infusion. Tislelizumab and ociperlimab must not be concurrently administered with any other drug.

At the end of each infusion period, the line will be flushed with enough normal saline to make sure the complete doses of study treatments are administered.

As a routine precaution, after infusion of all study treatment is complete on Day 1 of Cycle 1 and Cycle 2, patients must be monitored for ≥ 120 minutes afterward in an area with resuscitation equipment and emergency agents. From Cycle 3 onward, a ≥ 60-minute monitoring period is required in an area with resuscitation equipment and emergency agents.

Guidelines for treatment interruption, or discontinuation and for the management of irAEs and infusion-related reactions are provided in detail in **Section 11.3, Section 11.5 and**[**Appendix 10**](#附录10).

Refer to the Pharmacy Manual/prescribing information for detailed instructions on drug preparation, storage, and administration.

- - 1. Handling of Overdose

Any overdose or incorrect administration of study treatments will be recorded on the administration eCRF. AEs associated with an overdose or incorrect administration of study treatments will be recorded on the AE eCRF. In the event of an overdose or incorrect administration of study treatments, the study sponsor or designee should be notified as soon as possible.

- - 1. Investigational Medical Products Accountability

The investigational medicinal products (IMPs) required for completion of this study are tislelizumab and ociperlimab. The study sites will acknowledge receipt of IMPs. Any damaged shipments will be replaced.

Accurate records of all IMPs received, dispensed, returned, and disposed should be maintained in the site’s Drug Inventory Log. Refer to the Pharmacy Manual for details of IMPs management.

- - 1. Dose Modification or Delay

Reasons for dose modifications or delays, the supportive measures taken, and the outcome will be documented in the patient's chart and recorded on the eCRF.

**Tislelizumab and Ociperlimab**

There will be no dose reduction for tislelizumab or ociperlimab in this study. The investigator is required to make every effort to maintain dose intensity in patients.

Treatment with immunotherapy drugs may be temporarily suspended if the patient experiences a toxicity that is considered related to immunotherapy drugs and requires a dose to be withheld. Hold both tislelizumab and ociperlimab (either drug can not be administered alone; chemotherapy drugs can be continued for the first 8 cycles if toxicities are not caused by chemotherapy drugs ). Treatment with study treatments should resume as soon as possible after the AEs recover to Grade 1 or baseline (whichever is more severe) and within 12 weeks after the last dose of study treatments. If the administration of study treatments can resume within ≤ 10 days, study treatments should be administered in the current cycle. If study treatments need to be withheld for > 10 days, they should be omitted from the current cycle and administration should restart in the next cycle. If the patient is unable to resume study treatments within 12 weeks after the last dose, then the patient should be discontinued from treatment. If the patient is unable to resume study treatments within 12 weeks after the last dose because of unforeseen non-drug-related reasons, continued treatment may be allowed if approved by the investigator.

If the timing of a protocol-mandated study visit coincides with a holiday, weekend, or other event, the visit will be scheduled on the nearest feasible date (**refer to the visit window in Appendix 1**), with subsequent dosing continued in 21-day intervals accordingly, but the time between 2 consecutive doses of tislelizumab or ociperlimab should be at least 14 days.

Management guidelines for irAEs and infusion-related reactions in patients treated with the study treatments are presented in **Appendix 10** and **Section 11.5.12.**

**Gemcitabine and Cisplatin**

Hematologic toxicity: platelet and neutrophil counts must be performed prior to each gemcitabine administration. Patients may continue gemcitabine treatment if their ANC is ≥ 1.5 x 10^9^/L and platelet count is ≥ 100 x 10^9^/L at the start of treatment. Otherwise, treatment needs to be held until abnormalities in hematology recover. Treatment with tislelizumab and ociperlimab can be proceeded as planned. At initiation of subsequent treatment cycles, dose modifications should be based on the nadir values of hematology parameters or the most severe non-hematologic toxicities in the previous treatment cycle. Treatment may be delayed to allow sufficient recovery time if relevant tests do not meet criteria, and treatment may be delayed for up to 3 weeks until neutrophils and platelets recover, otherwise the patient will be discontinued from treatment. Dose reductions for hematologic toxicities after patient recovery are presented in Table 9. For patients receiving gemcitabine, hematology should be monitored prior to each dose and if myelosuppression is detected prior to gemcitabine administration on Day 8,dose modifications or treatment interruptions will be managed according to the guidelines in Table 10.

Table 9: Dose Modification Guidelines for Gemcitabine (on Day 1) Due to Hematologic Toxicities Occurred in the Previous Treatment Cycle

| Nadir neutrophil count  (x 10^9^ /L) |  | Nadir platelet count  (x 10^9^ /L) | Percentage of gemcitabine in the full dose regimen |
| --- | --- | --- | --- |
| ≥ 0.5 | and | ≥ 50 | 100% |
| < 0.5 | and | ≥ 50 | 75% |
| Any condition | and | <50 | 75% |
| Any condition | and | < 50 with bleeding | 50% |
| < 1.0 with fever ≥ 38.5ºC | and | Any condition | 75% |

Table 10: Dose Modification Guidelines for Gemcitabine (on Day 8) Due to Hematologic Toxicities Occureed During the Same Treatment Cycle

| Neutrophil count (x 10^9^ /L) |  | Platelet count (x 10^9^ /L) | Percentage of gemcitabine in the full dose regimen |
| --- | --- | --- | --- |
| ≥ 1.0 | and | ≥ 100 | 100% |
| 0.5-0.999 | Or | 50-99 | 75% |
| < 0.5 | Or | < 50 | Not dose |

Patients whose gemcitabine administration is interrupted on Day 8 may resume their next dose on time if the toxicities have resolved to ≤ CTCAE Grade 2. If the patient cannot receive gemcitabine on Day 8, the dosing schedule (duration) will remain unchanged and this dose will be skipped and will not be made up in the future.

Non-hematologic toxicities: In general, for severe (Grade 3 or 4) non-hematologic toxicities and nausea/vomiting, gemcitabine treatment should be interrupted or subjected to a 50% dose reduction at the discretion of the investigator.

Dose modification guidelines based on toxicity grade and severity for non-hematologic toxicities are summarized in Table 11.

Table 11: Dose Modification Guidelines for Gemcitabine Due to Non-Hematologic Toxicities

|  | **Grade 2** | **Grade 3** | **Grade 4** |
| --- | --- | --- | --- |
| First occurrence | Interrupt treatment until toxicity resolves to Grade 0 to 1, then continue gemcitabine treatment at the same initial dosage as much as possible to prevent recurring toxicity | Interrupt treatment until toxicity resolves to Grade 0 to 1, then continue gemcitabine treatment at 75% of the initial dosage as much as possible to prevent recurring toxicity. | Discontinue gemcitabine treatment unless, in the opinion of the investigator, it is in the best interest of the patient to continue the treatment at 50% of the initial dosage after the toxicity resolves to Grade 0 to 1 |
| 2^nd^ appearance of same toxicity | Interrupt treatment until toxicity resolves to Grade 0 to 1, then continue gemcitabine treatment at the at 75% of the initial dosage as much as possible | Interrupt treatment until toxicity resolves to Grade 0 to 1, then continue gemcitabine treatment at the at 50% of the initial dosage as much as possible |  |
| 3^rd^ appearance of same toxicity | Interrupt treatment until toxicity resolves to Grade 0 to 1, then continue gemcitabine treatment at the at 50% of the initial dosage as much as possible | Permanently discontinue gemcitabine treatment |  |
| 4^th^ appearance of same toxicity | Permanently discontinue gemcitabine treatment |  |  |

Cumulative and dose-related renal impairment is the major dose-limiting toxicity associated with cisplatin. The investigator will determine whether hydration is required during cisplatin treatment to reduce nephrotoxicity based on the patient's specific condition.

Tinnitus and or high-pitched hearing loss were reported in 31% of patients treated with cisplatin, and these effects are more common and more severe during repeated dosing. Neurotoxicity is charcterized by peripheral neuropathy, including sensory and motor nerves neuropathy, may occur in some patients.

Based on the common toxicities of cisplatin, dose modifications guidelines are as follows:

| **Toxicity** | **Grade** | **Treatment** |
| --- | --- | --- |
| Renal toxicity | Grade 1 to 2 | Interrupt treatment until toxicity resolves to Grade 0 or baseline, then continue cisplatin treatment at the same initial dosage as much as possible; permanently discontinue treatment if toxicity recurs |
|  | Grade 3 to 4 | Stop cisplatin treatment |
| Ototoxicity | Grade 1 to 2 | Delay cisplatin treatment until resolve to Grade 0 or baseline |
|  | Grade 3 to 4 | Stop cisplatin treatment |
| Neurotoxicity | Grade 1 to 2 | Delay cisplatin treatment until resolve to Grade 0 or baseline |
|  | Grade 3 to 4 | Stop cisplatin treatment |

For dose modification of cisplatin, the investigator should make adjustments according to the patient's specific conditions, referring to the prescribing information for relevant products.

- - 1. Disposal and Destruction

After completion of the study, all unused study treatments will be inventoried and packaged for return shipment by the hospital unit pharmacist or other designated study site personnel.

Prior and Concomitant Therapies

- 1. Prior Therapies

The eligibility criteria require that the patient must not have received prior systemic therapy (including chemotherapy and immune checkpoint inhibitors such as anti-PD-1, anti-PD-L1/L2, anti-CTLA-4, anti-TIGIT) for biliary tract tumors.

- 1. Concomitant Therapies

Most concomitant medications and therapies deemed necessary and in keeping with local standards of medical care at the discretion of the investigator for the supportive care (e.g, anti-emetics, antidiarrheals) and in a patient’s interest are allowed. Patients should receive full supportive care, including epoetin and other hematopoietic growth factors, transfusions of blood and blood products, antibiotics, antiemetics, and/or other applicable medications, as needed.

All concomitant medications will be recorded on the eCRF including all prescription and over‑the‑counter drugs, herbal supplements, and medications and fluids administered by intravenous infusion. If changes (dose, stop, or start) in concomitant medications occur during the study, documentation of drug dosage, frequency, route, date, and reason for use will be recorded in the eCRF.

Patients with active hepatitis B defined as either detectable HBsAg or HBV DNA at baseline, must initiate or continue effective antiviral therapy during the study to decrease potential viral re-activation risk. Peg-IFN, tenofovir and entecavir are recommended in the American Association for the Study of Liver Disease (AASLD) guideline because they lack resistance with long-term use**^52-53^**. The investigator may use other antiviral agents, if appropriate, following local guidelines. Management of antiviral therapy is at the discretion of the investigator.

Systemic corticosteroids required for the control of infusion reactions or irAEs must be tapered over at least 1 month and be at non-immunosuppressive doses (≤ 10 mg/d of prednisone or equivalent) before the next treatment cycle. The short-term use of steroids as prophylactic treatment (e.g, in patients with contrast allergies to diagnostic imaging contrast dyes) is permitted. After discontinuation of immunotherapeutic drugs due to reasons such as toxicities, the use of corticosteroids and other drugs will not be affected by the investigational chemotherapeutic drugs.

- 1. Excluded (Prohibited or Restricted) Therapies

The following medications are prohibited or restricted at the time of Screening and during the administration of tislelizumab and ociperlimab:

- Immunosuppressive agents (except to treat a drug-related TEAE).
- Systemic corticosteroids > 10 mg daily (prednisone or equivalent), except to treat or control a drug-related TEAE or for short-term use as prophylactic treatment.
- Patients should have avoided alcohol completely and avoid other addictive drugs during the study.
- Live vaccines within 28 days prior to the first dose of study treatment(s) and 60 days following the last dose of study treatment(s).
- Herbal remedies with immune-stimulating properties (e.g, mistletoe extract) or that are known to potentially interfere with liver or other major organ functions (e.g, hypericin). Patients must notify the investigator of all herbal remedies used during the study.
- Radiation therapy is not allowed.
- Use of potentially hepatotoxic drugs in patients with impaired hepatic function should be carefully monitored.
- Use of potentially nephrotoxic drugs in patients with impaired renal function should be carefully monitored.

Study Assessments and Procedures

A table of scheduled study assessments is provided in [**Appendix 1**](#APPX1). Patients will be closely monitored for safety and tolerability throughout the study. All assessments must be performed and documented in the medical record for each patient.

Study treatment administration will occur only if the clinical assessment and local laboratory test values (that must be available before any administration) have been reviewed and found to be acceptable per protocol guidelines.

If the timing of a protocol-mandated study visit coincides with a holiday, weekend, or other event, the visit should be scheduled on the nearest feasible date (**refer to the visit window in Appendix 1**), with subsequent dosing continued in 21-day intervals accordingly.

- 1. Screening

Refer to the inclusion criteria (**Section 7.1**) for detailed requirements for Screening.

Patients who agree to participate in this study will sign the ICF prior to undergoing any screening procedure. Screening evaluations may be repeated as needed within the Screening period; the investigator is to assess patient eligibility according to the latest screening assessment results.

Results of routine assessments performed per standard of care ≤ 28 days prior to first dose may be used for the purposes of screening rather than repeating the standard‑of‑care tests unless otherwise indicated.

- - 1. Demographic Data and Medical History

Demographic data will include age, gender, and self-reported race/ethnicity.

Medical history should include any history of clinically significant diseases, surgeries, or cancer history (including prior anticancer therapies or surgeries); reproductive status (i.e, of childbearing potential or no childbearing potential); history of alcohol consumption and tobacco (i.e, yes or no); and all medications (e.g, prescription drugs, over-the-counter drugs, herbal or homeopathic remedies, nutritional supplements) used by the patient within 30 days before the first dose of study treatment. If appropriate, clinically significant disease should be graded according to NCI-CTCAE v5.0 and reported in the medical history report.

Cancer history will include an assessment of prior surgery, prior radiotherapy, and prior drug therapy including start and stop dates, best response, and reason(s) for discontinuation, and will also include information about macrovascular invasion and/or extrahepatic spread (present or absent). Information on radiographic studies performed before study entry may be collected for review by the investigator.

- - 1. Women of Childbearing Potential and Contraception

Childbearing potential is defined as being physiologically capable of becoming pregnant. **Refer to Appendix 6** for contraception guidelines and definitions of “women of childbearing potential” and “no childbearing potential”.

- - 1. Informed Consent Forms and Screening Log

Voluntary, written, informed consent forms (ICFs) for participation in the study must be obtained before performing any study-specific procedures. ICFs for enrolled patients and for patients who are screened but not enrolled will be maintained at the study site.

All screening evaluations must be completed and reviewed to confirm that patients meet all eligibility criteria before entering the study. The investigator will record details of all patients screened and to confirm eligibility or record reason(s) for screening failure, as applicable.

- - 1. Pulmonary Function Test

Patients who are suspected of having or known to have serious respiratory conditions or who exhibit significant respiratory symptoms unrelated to the underlying cancer will undergo pulmonary function testing which may include, but is not limited to, pulmonary function tests and assessment of diffusion capacity done during the screening period to assist the determination of suitability for the study.

- 1. Enrollment
     1. Confirmation of Eligibility

The investigator will assess the eligibility of each patient. All results from the screening procedures and relevant medical history must be available before eligibility can be determined. All inclusion criteria must be met and none of the exclusion criteria may apply. No eligibility waivers will be granted.

- - 1. Patient Study Number

After obtaining informed consent, study site personnel will assign an unique patient number to a potential study patient.

- - 1. Dispensation of Study Treatment

All study treatments will be dispensed and administered as described in **Section 8.1**.

- 1. Safety Assessments
     1. Vital Signs

Vital signs include the measurements of body temperature (℃), heart rate, and blood pressure (systolic and diastolic) while the patient is in a seated position after resting for 10 minutes.

Height (baseline only) and weight should be measured and recorded on the eCRF.

For the infusions during the first two cycles, the patient’s vital signs should be collected within 60 minutes before the infusion and during and 30 minutes after the infusion. For subsequent cycles, vital signs will be collected within 60 minutes before the infusion and if clinically indicated, during, and 30 minutes after the infusion. Patients will be informed about the possibility of delayed post-infusion symptoms and instructed to contact their study physician if they develop such symptoms.

- - 1. Physical Examination

During the Screening Visit, a complete physical examination will be conducted including evaluation of 1) head, eyes, ears, nose, throat; 2) cardiovascular; 3) dermatological; 4) musculoskeletal; 5) respiratory, 6) gastrointestinal; and 7) neurological systems. Any abnormality identified at baseline will be graded according to NCI‑CTCAE v5.0 and recorded on the medical history eCRF with appropriate disease/condition terms. Height (baseline only) and weight should be measured and recorded in the eCRF.

In addition, the investigator is required to solicit patients regarding changes in vision, visual disturbance, or ocular inflammation at each scheduled study visit during study treatment. For any change in vision, referral to an appropriate specialist will be made for further management guidance.

At subsequent visits (and as clinically indicated), limited, symptom-directed physical examinations will be performed. Changes from baseline will be recorded. New or worsened clinically significant abnormalities are to be recorded as AEs on the eCRF. Refer to **Section 11.5** regarding AE definitions and reporting and follow-up requirements.

- - 1. Eastern Cooperative Oncology Group (ECOG) Performance Status

Patients’ ECOG PS will be assessed at Screening Visit, during the study, and at the Safety Follow-up Visit (Table 12).

Table 12: Eastern Cooperative Oncology Group (ECOG) Performance Status to Grading System

| Grade | Performance Status |
| --- | --- |
| 0 | Fully active, able to carry on all pre-diseases performance without restriction. |
| 1 | Restricted in physically strenuous activity but ambulatory and able to carry out work of a light or sedentary nature, e.g, light house work, office work. |
| 2 | Ambulatory and capable of all self-care but unable to carry out any work activities. Up and about > 50% of waking hours. |
| 3 | Capable of only limited self-care; confined to bed or chair more than 50% of waking hours. |
| 4 | Completely disabled; cannot carry on any self-care; totally confined to bed or chair. |
| 5 | Death. |

- - 1. Laboratory Safety Assessments

Local laboratory assessments on hematology, serum chemistry, coagulation, and urinalysis will be conducted, of which certain elements will be collected as specified in **Appendix 1**.

If laboratory tests at Screening are not performed within 7 days before Day 1 of Cycle 1, these tests should be repeated and reviewed before study treatment administration. The hematology and serum chemistry tests specified in **Appendix 1** should be reviewed within 48 hours prior to subsequent study treatment administration.

The assessments are specified as follow:

1. Hematology (complete blood count [CBC], including red blood cell [RBC] count, hemoglobin, hematocrit, white blood cell [WBC] count with differential [neutrophils], and platelet count). Hematology will be performed at Screening, during treatment, and at EOT Visit; As for treatment period, it will be performed before administration on Day 1 (D1) and Day 8 (D8) of each cycle during treatment with tislelizumab in combination with ociperlimab and GP regimen. After the completion of chemotherapy, it will be performed before administration on D1 of each subsequent cycle.
2. Serum chemistry (glucose, urea nitrogen, urea, creatinine, sodium, potassium, magnesium, chloride, calcium, phosphorus, direct bilirubin, total bilirubin, ALT, AST, alkaline phosphatase (ALP), lactate dehydrogenase , total protein, albumin). Hematology will be performed at Screening, during treatment, and at EOT Visit; As for treatment period, it will be performed before administration on Day 1 (D1) and Day 8 (D8) of each cycle during treatment with tislelizumab in combination with ociperlimab and GP regimen. After the completion of chemotherapy, it will be performed before administration on D1 of each subsequent cycle.
3. Coagulation parameters (international normalized ratio, prothrombin time, and activated partial thromboplastin time). Hematology will be performed at Screening, during treatment, and at EOT Visit; As for treatment period, it will be performed before administration on Day 1 (D1) and Day 8 (D8) of each cycle during treatment with tislelizumab in combination with ociperlimab and GP regimen. After the completion of chemotherapy, it will be performed before administration on D1 of each subsequent cycle.
4. Urinalysis (including, but not limited to urine specific gravity, pH, glucose, protein, ketones). Hematology will be performed at Screening, during treatment, and at EOT Visit; As for treatment period, it will be performed before administration on Day 1 (D1) and Day 8 (D8) of each cycle during treatment with tislelizumab in combination with ociperlimab and GP regimen. After the completion of chemotherapy, it will be performed before administration on D1 of each subsequent cycle.
5. CK/CK-MB (creatine kinase, creatine kinase isoenzyme) will be performed at Screening, during treatment and at EOT Visit. As for treatment period, it will be performed before administration on Day 1 (D1) and Day 8 (D8) of each cycle during treatment with tislelizumab in combination with ociperlimab and GP regimen. After the completion of chemotherapy, it will be performed before administration on D1 of each subsequent cycle.
6. Urine pregnancy test must be performed within 7 days prior to the first dose among female patients of childbearing potential and repeated during treatment as clinically indicated. A serum pregnancy test must be performed if the urine pregnancy test is positive or equivocal.
7. Thyroid function tests (thyroid stimulating hormone [TSH], free T3, free T4) will be performed at Screening, every 9 weeks (± 7 days) during treatment, and at EOT Visit.
8. AFP, CA19-9, CA125, andCEA tests. These tests will be performed at Screening, every 9 weeks (± 7 days) during treatment, and at EOT Visit.
9. HBsAg, anti-HBsAg antibody, hepatitis B core antibody (HBcAb) tests will be performed as clinically indicated during the study.
10. HCV serology (anti-HCV antibody) will be tested as clinically indicated during the study.
11. HBV DNA and HCV RNA HBV DNA or HCV RNA testings will be performed when antibody was positive

During the study, the investigator may adjust or add relevant examinations or assessments as needed based on the clinical symptoms and specific conditions of a patient.

- - 1. Pulmonary Function Test

Pulmonary function tests including spirometry and assessment of diffusion capacity are to be performed for all patients, as clinically indicated, at Screening to assist patient eligibility determination, and as clinically indicated during the study treatment.

- - 1. Electrocardiograms

12-lead ECGs will be performed for all patients at Screening, before dosing on D1 of each treatment cycle, and at EOT Visit. ECG recordings will be performed after the patient has been resting for at least 10 minutes, and a repeat ECG will be performed to confirm abnormal findings, if any.

- - 1. Adverse Events

AEs will be graded and recorded throughout the study according to NCI‑CTCAE v5.0. The characterization of toxicities will include severity, duration, and time to onset.

All AE, including SAEs, will be collected as described in Section 11.3 and Section 11.4. At the end of treatment, any ongoing AE considered related to study treatment will be followed until the event has resolved to baseline or ≤ Grade 1, the event is assessed by the investigator as stable, the patient is lost to follow-up, or the patient withdraws consent.

- 1. Biomarker Testing

Enrolled patients are required to provide tumor tissues (formalin-fixed paraffin-embedded tissue or fresh unstained slides) for the detection of PD-L1 and TIGIT expression in a central laboratory to explore the correlation with disease status, treatment response/prognosis. Refer to the laboratory manual for instructions on the sample collection.

Note:Written informed consent is required before obtaining fresh tumor biopsies. Tumor tissues need to originate from core or punch biopsy. Tumor tissue from fine-needle aspiration is not acceptable. For fresh biopsy, acceptable samples include core needle biopsies for non-superficial tumor tissue or excisional, incisional, punch, or forceps biopsies for cutaneous, subcutaneous, or mucosal lesions. In case of submitting unstained cut slides, freshly cut slides should be submitted to the testing laboratory within 14 days from when the slides are cut. Tumor tissue should be of good quality based on total and viable tumor content. Fine‑needle aspiration, brushing, cell pellets from pleural effusion, and lavage samples are not acceptable.

Instructions for the processing, storage, and shipping of samples will be provided in the study laboratory manual.

- 1. Tumor and Response Evaluation

Tumor imaging will be performed ≤ 28 days before the first dose of study treatment. Results of standard-of-care tests or examinations performed before informed consent has been obtained and ≤ 28 days before the first dose of study treatment may be used for the purposes of screening rather than repeating the standard-of-care tests. During the study, tumor imaging will be performed approximately every 9 weeks (± 7 days). Tumor assessments are required to be performed on schedule regardless of whether study treatment has been administered or held; they should not be adjusted for possible delays in cycles. Tumor assessments should continue until disease progression is determined by the investigator. Patients who discontinue study treatment early for reasons other than disease progression (e.g, toxicity) will continue to undergo tumor assessments following the original plan until the patient experiences disease progression or death, withdraws consent, is lost to follow-up, or until the study terminates, whichever occurs first.

Screening assessments and each subsequent assessment of the tumor must include computed tomography (CT) scans (with oral/intravenous contrast, unless contraindicated) or magnetic resonance imaging (MRI) of the chest, abdomen, and pelvis. Other known or suspected sites of disease (neck, brain, etc) must be included in the imaging assessments.

All measurable and evaluable lesions will be assessed and documented at Screening Visit and reassessed at each subsequent tumor assessments. The same radiographic procedure used to assess disease sites at Screening is required to be used throughout the study (e.g, the same contrast protocol for CT scans or MRI).

• Imaging of the brain (preferably MRI) at baseline is required for all screened patients. Screening evaluations will be performed within 28 days before the first dose of study treatment.

• If a CT scan for tumor assessment is performed on a positron-emission tomography (PET)/CT scanner, the CT acquisition must be consistent with the standards of a diagnostic CT scan.

• Bone scans (Technetium-99m [Tc-99m]) or PET should be performed at Screening if clinically indicated. If bone metastases are present at Screening and cannot be seen on CT or MRI scans, Tc-99m or PET bone scans should be repeated when a CR is suspected in target lesion or when progression in bone is suspected.

• CT scans of the neck or extremities should be performed at Screening only if clinically indicated and should be followed throughout the study if there is evidence of metastatic disease in these regions at Screening.

• At the investigator’s discretion, other methods of assessment of target lesion and non-target lesions per RECIST v1.1 may be used.

Response will be assessed by the investigator using RECIST v1.1 (see **Appendix 9**).

After documentation of initial response (CR or PR), confirmation of tumor response should occur ≥ 4 weeks but ≤ 6 weeks after the initial response.

- 1. Visit Windows

All visits must occur within ± visit windows from the scheduled date, unless otherwise noted (see **Appendix 1**). All assessments will be performed on the day of the specified visit, unless an acceptable time window is indicated. Assessments scheduled on the day of study treatment administration (Day 1) of each cycle should be performed before study treatment infusion/dose unless otherwise noted. Laboratory results are required to be reviewed before dosing.

If the timing of a protocol-mandated study visit coincides with a holiday, weekend, or other event, the visit should be scheduled on the nearest feasible date (**refer to the visit window in Appendix 1**), with subsequent dosing continued in 21-day intervals accordingly.

- 1. Unscheduled Visits

Unscheduled visits may be performed at any time at the patient’s or the investigator’s request and may include vital signs/focused physical examinations, ECOG performance status, AEs review, concomitant medications and procedures reviews, radiographic assessments, physical examination of liver, spleen, and lymph nodes; disease-related constitutional symptoms, and hematology and chemistry laboratory assessments. The date,reason(s) for an unscheduled visit and results must be recorded on the eCRF.

If an unscheduled visit is necessary to assess toxicity or for suspected disease progression, then diagnostic tests may be performed based on the investigator assessment as appropriate, and the results of these tests should be entered on the unscheduled visit eCRF.

- 1. End of Treatment (EOT) Visit

Patients who discontinue from study treatment for any reason will be asked to return to the clinic for the EOT Visit, which is required to be conducted within 30 days after the last dose of study treatment(s) or before the initiation of a new anticancer treatment, whichever occurs first. If routine laboratory tests (e.g, hematology, clinical chemistry) are completed ≤ 7 days before the EOT Visit, these tests do not need to be repeated. Tumor assessment is not required at the EOT Visit if ≤ 9 weeks have passed since the last assessment. If the study treatment(s) were initially interrupted and then permanently discontinued due to AE(s), the EOT Visit may occur later, but not later than the permitted time of dose delay plus 7 days.

- 1. Safety follow-up Visit

Telephone contacts with patients should be conducted to assess irAEs and concomitant medications (if appropriate, i.e, associated with an irAE or is a new anticancer therapy) at 60 days (± 14 days) and 90 days (± 14 days) after the last dose of tislelizumab an ociperlimab, respectively, regardless of whether or not the patient starts a new anticancer therapy. If a patient reports a suspected irAE at a telephone follow-up contact, the investigator should arrange an unscheduled visit if further assessment is indicated.

Information on AEs and SAEs will be collected up to 30 days after the last dose of study treatment or initiation of a new anticancer therapy, whichever occurs first, and information on irAEs will be collected up to 90 days after the last dose of study treatment (regardless of whether or not a new anticancer therapy is initiated). After the discontinuation of study treatment, the investigator should continue to report any SAEs that are considered to be related to the study treatment(s).

The End-of-Treatment Visit at which a response assessment shows progressive disease (PD), resulting in patient discontinuation, could be used as the Safety Follow-up Visit, if it occurs 30 days (± 7 days) after the last study treatment.

- 1. Survival Follow-up Visit

Patients will be followed for survival and to obtain information on subsequent anticancer therapy information after discontinuation of study treatment via telephone calls, patient's chart, and/or clinic visits approximately every 3 months (± 14 days) after the EOT Visit until death, loss to follow-up, withdrawal of consent, or study termination.

Safety Monitoring and Reporting

The investigator is responsible for the monitoring and documentation of events that meet the criteria and definition of an AE or SAE as provided in this protocol.

- 1. Risks Associated With Study Treatments
     1. Risks Associated with Ociperlimab and Tislelizumab

Tislelizumab and ociperlimab are investigational medical products that are currently under clinical development. The following recommendation is based on results from nonclinical and clinical studies with tislelizumab and ociperlimab, and published data on other molecules within the same biologic class.

The PD-L1/ PD-1 pathway is involved in peripheral immune tolerance; therefore, such therapy may increase the risk of irAEs, specifically the induction or enhancement of autoimmune conditions.

Although most irAEs observed with immunomodulatory agents have been mild and self-limiting, such events should be recognized early and treated promptly to avoid potential major complications. Guidelines for the assessment and management of suspected irAEs are provided in [**Appendix 10**](#附录10).

- - 1. Risks Associated With Gemcitabine and Cisplatin

The most common adverse reactions observed during the combination therapy of cisplatin and gemcitabine are gastrointestinal reactions (diarrhea, nausea, vomiting, and mucositis), hematological reactions (neutropenia, thrombocytopenia), and neurological reactions (acute, dose-cumulative, peripheral sensory neuropathy), etc. Most of AEs are manageable through symptomatic management, drug dose reduction, or treatment discontinuation according to the established toxicity management procedures.

Refer to the prescribing information of cisplatin and gemcitabine for further details on the safety profiles of each drug.

- 1. General Plan to Manage Safety Concerns
     1. Eligibility Criteria

The eligibility criteria will be used to select patients so as to guard the safety. Results from the nonclinical toxicology studies and clinical data with tislelizumab, ociperlimab, gemcitabine and cisplatin as well as the nonclinical/clinical data from other anti-PD‑L1/PD-1 and anti-TIGIT inhibitors are considered. Specifically, patients at risk for study-emergent active autoimmune diseases or with a history of autoimmune diseases that may relapse, patients who have undergone allogeneic stem cell or organ transplantation, and patients who have received a live vaccine ≤ 28 days before the first dose of study treatment are excluded from the study **(refer to Section 7.2 for the full list of exclusion criteria)**.

- - 1. Safety Monitoring Plan

Safety will be evaluated in this study through the monitoring of all AEs (serious and non-serious), which will be defined and graded according to NCI-CTCAE v5.0. Patients will be assessed for safety (including laboratory values) according to the Schedule of Assessments in **Appendix 1**. Clinical laboratory results must be reviewed prior to the start of each cycle.

In this study, all enrolled patients will be evaluated clinically and undergo standard laboratory tests before and at regular intervals during their participation. Safety evaluations will consist of medical interviews, recording of AEs, physical examinations, laboratory measurements (hematology, chemistry, etc.) and other assessments. In addition, patients will be closely monitored for the development of any signs or symptoms of autoimmune conditions and infection.

Administration of study treatment will be performed in a setting where emergency medical equipment and staff who are trained to respond to medical emergencies are available.

At the end of treatment, any ongoing AE considered related to study treatment will be followed until the event has resolved to baseline or ≤ Grade 1, the event is assessed by the investigator as stable, the patient is lost to follow-up, or the patient withdraws consent.

All AEs will be recorded during the study and for up to 30 days after the last dose of study treatment or until the initiation of new anticancer therapy, whichever occurs first. All irAEs should be recorded up to 90 days after the last dose of tislelizumab and ociperlimab regardless of whether or not the patient starts a new anticancer therapy. All drug-related SAEs will be recorded by the investigator after treatment discontinuation until patient death or loss to follow-up, whichever occurs first.

The potential safety issues anticipated in this study, as well as measures intended to avoid or minimize such toxicities, are outlined in the following sections.

- 1. Adverse Events
     1. Definitions and Reporting

An AE is defined as any unfavorable and unintended sign (including an abnormal laboratory finding), symptom, or disease (new or exacerbated) . AE is temporally associated with the use of a study treatment, whether considered related to study treatment or not.

Examples of an AE include:

- Worsening of a chronic or intermittent preexisting condition, including an increase in severity, frequency, duration, and/or has an association with a significantly worse outcome.
- New condition(s) detected or diagnosed after study treatment administration even though it may have been present before the start of the study.
- Signs, symptoms, or the clinical sequelae of a suspected interaction.
- Signs, symptoms, or the clinical sequelae of a suspected overdose of either the study treatment or a concomitant medication (an overdose *per se* should not be reported as an AE or SAE).

When an AE or SAE occurs, it is the responsibility of the investigator to review all documentation (e.g, hospital charts, laboratory reports and diagnostics reports) relative to the AE/SAE. The investigator will then record all relevant information regarding an AE or SAE on the eCRF. However, there might be instances when copies of medical records for certain cases are requested by the sponsor. In this instance, all patient identifiers will be redacted on the copies of the medical records before submission to the sponsor.

- - 1. Assessment of Severity

The investigator should make an assessment of severity for each AE and SAE reported during the study. AEs and SAEs should be assessed and graded based on NCI-CTCAE v5.0, if appropriate.

Toxicities that are not specified in the NCI‑CTCAE will be defined as follow:

- Grade 1: Mild; asymptomatic or mild symptoms, clinical or diagnostic observations only, intervention not indicated;
- Grade 2: Moderate; minimal, local, or noninvasive intervention indicated; limiting age‑appropriate instrumental activities of daily living (ADL);
- Grade 3: Severe or medically significant but not immediately life-threatening; hospitalization or prolongation of hospitalization indicated; disabling; limiting self‑care ADL;
- Grade 4: Life-threatening consequences; urgent intervention indicated;
- Grade 5: Death related to AE.

NOTE: The terms “severe” and “serious” are not synonymous. Severity is a measure of intensity (e.g, grade of a specific AE, mild [Grade 1], moderate [Grade 2], severe [Grade 3], or life‑threatening [Grade 4]); whereas seriousness is classified by the criteria based on the regulatory definitions. Seriousness serves as the guide for defining regulatory reporting obligations from the sponsor to applicable regulatory authorities, as described in Section **11.5.2.3**.

- - 1. Assessment of Causality

The investigator is obligated to assess the relationship between the study treatment and the occurrence of each AE or SAE using their best clinical judgment. Alternative causes, such as natural history of the underlying diseases, concomitant therapies, and other risk factors, and the temporal relationship of the AE or SAE to the study treatment will be considered and investigated. The investigator should also consult the Investigator’s Brochure and/or Prescribing Information in the determination of his/her assessment.

There might be situations in which an SAE occurs, and the investigator has minimal information to include in the initial report. However, it is very important that the investigator always assesses causality for every SAE prior to transmission of the SAE report, since the causality assessment is one of the criteria used when determining regulatory reporting requirements. The investigator may change his/her opinion of causality considering follow-up information, amending the SAE report accordingly.

The causality of each AE should be assessed and classified by the investigator as “related” or “not related”. An AE is considered related if there is “a reasonable possibility” that the AE may have been caused by the study treatment (i.e, there are facts, evidence, or arguments to suggest possible causation). A number of factors should be considered in making this assessment, including:

- Temporal relationship of the AE to the administration of study treatment or study procedure
- Whether other possible causes have been identified
- Mechanism of action of the study treatment
- Biological plausibility

An AE should be considered “related to” study treatment if any of the following are met; otherwise, the event should be assessed as “not related”:

- There is clear evidence to suggest a causal relationship, and other possible contributing factors can be ruled out.
- There is evidence to suggest a causal relationship and the influence of other factors is unlikely.
- There is some evidence to suggest a causal relationship (e.g, the AE occurs within a reasonable time after administration of the study treatment). However, the influence of other factors may have contributed to the AE (e.g, the patient’s clinical condition or other concomitant AEs).
  - 1. Follow-up of Adverse Events

After the initial AE or SAE report, the investigator is required to proactively follow each patient and provide further information on the patient’s condition.

All AEs and SAEs documented at a previous visit/contact and designated as ongoing will be reviewed at subsequent visits/contacts.

All AEs and SAEs will be followed until resolution, the condition stabilizes or is considered chronic, the AE or SAE is otherwise explained, the patient is lost to follow-up, or the patient withdraws consent. The investigator will ensure that follow-up information will include any supplemental investigations that may have been indicated to elucidate the natural progression of the AE or SAE or to support the assessment of their causality to the study treatment. This may have included additional laboratory tests or investigations, histopathological examinations, radiographic imaging, or consultation with other health care professionals.

The investigator performs or arranges for the conduct of unscheduled measurements and/or evaluations to elucidate as fully as possible the nature and/or causality of the AE or SAE. The investigator is obligated to assist. If a patient dies during participation in the study or during a recognized follow-up period, the investigator will provide the sponsor with a copy of any postmortem findings including histopathology.

New or updated information will be recorded on the originally completed SAE report. The updated SAE report should be resent to the sponsor within the time frames outlined in **Section 11.5**.

- - 1. Laboratory Test Abnormalities

Only abnormal laboratory findings (e.g, clinical chemistry, hematology, coagulation, or urinalysis) or other abnormal assessments (e.g, ECGs, X-rays, or vital signs) that are judged by the investigator as clinically significant will be recorded as AEs or SAEs. This includes clinically significant abnormal laboratory findings or other abnormal assessments that are present at baseline and significantly worsen during the study. The definition of “clinically significant” is left to the judgment of the investigator. In general, these are the following laboratory test abnormalities or other abnormal assessments that

- Are associated with clinical signs or symptoms, or
- Require active medical intervention, or
- Lead to dose interruption or discontinuation, or
- Require close observation, more frequent follow-up assessments, or
- Require further diagnostic investigation.
  1. Definition of a Serious Adverse Event

A SAE is any untoward medical occurrence that, at any dose:

- Results in death
- Is life‑threatening.

Note: The term “life-threatening” in the definition of “serious” refers to an AE in which the patient is at risk of death at the time of the AE. It does not refer to an AE that hypothetically might have caused death if it is more severe.

- Requires hospitalization or prolongation of existing hospitalization.

Note: in general, hospitalization signifies that the patient has been admitted (usually involving at least an overnight stay) at the hospital or emergency ward for observation and/or treatment that would not have been appropriate in the physician’s office or outpatient setting.

- Results in disability/incapacity

Note: The term disability means a substantial disruption of a person’s ability to conduct normal life functions. This definition is not intended to include experiences of relatively minor medical significance, such as uncomplicated headache, nausea, vomiting, diarrhea, influenza, and accidental trauma (e.g, sprained ankle), which may interfere or prevent everyday life functions, but do not constitute a substantial disruption.

- Is a congenital anomaly/birth defect
- Is considered a significant medical AE by the investigator based on medical judgment (e.g, may jeopardize the patient or may require medical/surgical intervention to prevent the outcomes listed above).

The following are not considered as SAEs:

- Hospitalization for elective treatment of a pre‑existing condition that did not worsen from baseline.
- Hospitalization for social/convenience considerations.
- Scheduled therapy for the target disease of the study, including admissions for transfusion support or convenience.
  1. Timing, Frequency, and Method of Capturing Adverse Events and Serious Adverse Events
     1. Adverse Event Reporting Period

After informed consent has been signed, but prior to the administration of the study treatment, only SAEs should be reported.

After initiation of study treatment, all AEs and SAEs, regardless of relationship to study treatment, will be reported until either 30 days after last dose of study treatment or initiation of new anticancer therapy, whichever occurs first. All irAEs should be recorded up to 90 days after the last dose of study treatment regardless of whether or not the patient starts a new anticancer therapy. Once the AE reporting period has ended, the investigator should continue to report any SAEs or AEs of special interest that are believed to be related to study treatment.

- - 1. Reporting Serious Adverse Events
       1. Expedited Reporting of Serious Adverse Events

As soon as the investigator determines that an AE meets the protocol definition of an SAE, the event must be reported promptly to the sponsor or designee as described in **Table 13**.

Table 13: Timeframes and Documentation Methods for Reporting Serious Adverse Events (SAEs) to the Sponsor or Designee

|  | **Timeframe for Sending Initial Report** | **Documentation Method** | **Timeframe for Making Follow‑up Report** | **Documentation Method** | **Reporting method** |
| --- | --- | --- | --- | --- | --- |
| All SAEs | ≤ 24 hours after first knowledge of the event | SAE Form | Within 24 hours of knowledge of SAE follow-up | SAE Form | Email or fax SAE form |

Abbreviations: AE, adverse event; SAE, serious adverse event.

- - - 1. Completion and Transmission of the Serious Adverse Event Report

Once the investigator becomes aware of a SAE, he/she will report it to the study sponsor or designee within 24 hours. The SAE report will always be completed as thoroughly as possible, including all available details of the event.

If the investigator does not have all the information regarding a SAE, he/she must not wait to receive additional information before notifying the sponsor or designee of the serious adverse event and completing the form. This SAE form will be updated upon receipt of additional information.

The investigator must provide an assessment of causality in the SAE report as described in **Section 11.3.3**.

The sponsor will provide contact information for SAE receipt.

- - - 1. Regulatory Reporting Requirements for Serious Adverse Events

The investigator will report all SAEs to the sponsor in accordance with the procedures detailed in **Section 11.5.2**. The study sponsor should report safety information to the local applicable regulatory authorities in accordance with applicable regulatory requirements.

The investigator, or responsible person according to local requirements, will comply with the applicable local regulatory requirements related to the reporting of SAEs to applicable regulatory authorities and the Institutional Review Board (IRB)/ Independent Ethics Committee (IEC).

When a study site receives an initial or follow-up safety report or other safety information (e.g, revised Investigator’s Brochure) from the sponsor, the investigator or designated responsible person is required to promptly notify the applicable institutions (such as IRB or IEC) based on applicable local regulations.

- - 1. Diagnosis Versus Recording Signs or Symptoms

If a diagnosis is known at the time of reporting, this should be recorded on the CRF (and SAE report, as applicable), rather than the individual signs and symptoms (e.g, record only hepatitis rather than elevated transaminases, bilirubin, or jaundice).

However, if a constellation of signs and/or symptoms cannot be medically characterized as a single diagnosis or syndrome at the time of reporting, each individual AE should be recorded as a SAE or AE on the eCRF (and SAE report, if applicable). If a diagnosis is subsequently established, it should replace the individual signs and/or symptoms as the AE term on the CRF and SAE report, if applicable.

- - 1. Adverse Events Occurring Secondary to Other Events

In general, AEs occurring secondary to other AEs (e.g, clinical sequelae or a cascade of AEs) should be identified by their primary cause. However, events that meet the criteria for separate reporting should be reported separately. For example, if severe vomiting results in dehydration, both severe vomiting and dehydration should be reported as separate SAE or AE on the CRF (and SAE report, if applicable). However, if a patient initially has a non-serious AE, and it subsequently becomes an SAE, both AEs should be reported separately on the eCRF. The onset dates of the events are all non-serious AEs and date that the events became serious will also be provided.

- - 1. Recording Persistent or Recurrent Adverse Events

A persistent AE is one that extends continuously, without resolution, between patient evaluation time points. If the severity of an AE changes, the time at which the AE escalates to its highest severity and the new severity grade will be recorded on the AE CRF.

A recurrent AE is one that occurs and resolves between patient evaluation time points, and subsequently recurs. All recurrent AEs should be recorded separately on the eCRF (and SAE report, if applicable).

- - 1. Disease Progression

Disease progression, which is expected in this study population and is measured as an efficacy endpoint, will not to be recorded as an AE term. Instead, the symptoms, signs that result from disease progression should be reported as the AE term(s). For instance, in a patient who presents with pleural effusion resulting from disease progression, the event term should be reported as “pleural effusion” instead of “disease progression.” Similarly, non-serious AEs that are clearly consistent with the pattern of progression of the underlying disease and are considered unequivocally due to disease progression should not be recorded. However, if there is any uncertainty to whether a non-serious AE is due to disease progression, it should be recorded as an AE. All SAEs and deaths regardless of relatedness to disease progression should be recorded and reported.

- - 1. Recording Deaths

Death is an outcome and not usually reported as a term. If the only information available is death and the cause of death is unknown, then the death is reported as an event, e.g, “death,” “death of unknown cause,” or “death unexplained”.

- - 1. Recording Pregnancy

If a female patient or the partner of a male patient becomes pregnant while receiving study treatment or within 120 days after the last dose of study treatment, a pregnancy report form must be completed and submitted to the sponsor within 24 hours to facilitate outcome follow-up. Information on the status of the mother and child will be forwarded to the sponsor. Generally, follow-up will be no longer than 6 to 8 weeks after the estimated delivery date. Any premature termination of the pregnancy will be reported.

While pregnancy itself is not considered to be an AE or SAE, any pregnancy complication or elective termination of a pregnancy for medical reasons will be recorded as an AE or SAE. Pregnancy should be recorded on the Pregnancy Report Form.

An abortion, whether accidental, therapeutic, or spontaneous, should be always reported as aSAE. Similarly, any congenital anomaly or birth defect in a child born to a patient exposed to the study treatment should be recorded and reported as a SAE.

- - 1. Recording Post-study Adverse Events

A post-study AE or SAE is defined as any AE that occurs after the AE/SAE reporting period, defined in Section **11.5.1**.

The investigators is not obligated to actively follow up information about AEs or SAEs in former patients. However, if the investigator learns of any SAE, including a death, at any time after a patient has been discharged from the study, and he/she considers the SAE related to the study treatment, the investigator will promptly notify the sponsor.

- - 1. Management of Adverse Events of Special Interest

As a routine precaution, after the infusions of study treatment during the first two cycles, patients must be monitored for ≥ 120 minutes afterward in an area with resuscitation equipment and emergency agents. If this is well tolerated during the first two cycles, from Cycle 3 onward, a ≥ 60‑minute monitoring period is required in an area with resuscitation equipment and emergency agents.

The management of infusion-related reactions, severe hypersensitivity reactions and irAEs as well as hepatic AEs are outlined below.

- - 1. Infusion-Related Reactions

The symptoms of infusion-related reactions include fever, chills/rigor, nausea, pruritus, angioedema, hypotension, headache, bronchospasm, urticaria, rash, vomiting, myalgia, dizziness, or hypertension. Severe reactions included acute respiratory distress syndrome, myocardial infarction, ventricular fibrillation, and cardiogenic shock. Patients will be closely monitored for such reactions. Immediate access to an Intensive Care Unit (ICU) or equivalent environment and appropriate medical therapy (including epinephrine, corticosteroids, IV antihistamines, bronchodilators, and oxygen) are required to be available to treat infusion-related reactions.

Treatment modifications for suspected infusion-related reactions due to study treatment(s) is provided in **Table 14**.

Table 14: Treatment Modification Guidelines for Symptoms of Infusion-Related Reactions Due to Study treatment(s)

| **AE Severity Graded According to the NCI-CTCAE** | **Treatment Modification** |
| --- | --- |
| **Grade 1 to mild**  Mild, transient reaction; infusion interruption not indicated; intervention not indicated. | Decrease infusion rate by 50%. Any worsening is closely monitored. Medical management as needed.  Subsequent infusions should be given after premedication and at the reduced infusion rate. |
| **Grade 2 to moderate**  Therapy or infusion interruption indicated but responds promptly to symptomatic treatment (e.g, antihistamines, NSAIDs, narcotics, intravenous fluids); prophylactic medications indicated for ≤ 24 hours. | Stop infusion. Infusion may be resumed at 50% of previous rate once infusion-related reactions have resolved or decreased to ≤ Grade 1 in severity. Any worsening is closely monitored. Proper medical management should be instituted as described below.  Subsequent infusions should be given after premedication and at the reduced infusion rate. |
| **Grade 3 – severe**  Prolonged (e.g., not rapidly responsive to symptomatic medication and/or brief interruption of infusion); recurrence of symptoms following initial improvement; hospitalization indicated for clinical sequelae. | Immediately stop the infusion. Proper medical management should be instituted as described below.  The patient should be withdrawn from study treatment(s) treatment. |
| **Grade 4 – life threatening**  Life-threatening consequences; urgent intervention indicated. | Immediately stop the infusion. Proper medical management should be instituted as described below.  The patient should be withdrawn from study treatment(s) treatment.  Hospitalization is recommended. |

Abbreviations: NCI-CTCAE, National Cancer Institute Common Terminology Criteria for Adverse Events; NSAIDs, nonsteroidal anti-inflammatory drugs.

Once the study treatment(s) infusion rate has been decreased by 50% or suspended due to an infusion‑related reaction, it must remain decreased for all subsequent infusions with premedication. If the patient has a second infusion-related reaction (≥ Grade 2) on the slower infusion rate, infusion should be discontinued, and the patient should be withdrawn from study treatment(s) treatment.

**NCI‑CTCAE Grade 1 or 2 Infusion Reactions**: Proper medical management should be instituted, as indicated per the type of reaction. This includes but is not limited to an antihistamine (e.g, diphenhydramine or equivalent), antipyretic (e.g, paracetamol or equivalent), and if considered indicated oral or IV glucocorticoids, epinephrine, bronchodilators, and oxygen. In the next cycle, the patient will receive oral premedication with an antihistamine (e.g, diphenhydramine or equivalent) and an antipyretic drug (e.g, paracetamol or equivalent), and the patient should be closely monitored for clinical signs and symptoms of an infusion reaction.

**NCI-CTCAE Grade 3 or 4 Infusion Reactions**: Proper medical management should be instituted immediately, as indicated per type and severity of the reaction. This includes, but is not limited to, oral or intravenous antihistamine, antipyretic drugs, glucocorticoids, epinephrine, bronchodilators, and oxygen.

- - 1. Severe Hypersensitivity Reactions and Flu-Like Symptoms

If hypersensitivity reaction occurs, the patient must be treated according to the best available medical practice as described in the Complete Guideline for Emergency Treatment of Anaphylactic Reactions**^54^** according to the Working Group of the Resuscitation Council (UK). Patients are instructed to report any delayed reactions to the investigator immediately.

In the event of a systemic anaphylactic/anaphylactoid reaction (typically manifested within minutes following administration of the drug/antigen, and characterized by respiratory distress; laryngeal edema; and/or intense bronchospasm; and often followed by vascular collapse or shock without antecedent respiratory difficulty; cutaneous manifestations such as pruritus and urticaria with/without edema; and gastrointestinal manifestations such as nausea, vomiting, abdominal pain, and diarrhea), the infusion must be immediately stopped and the patient discontinued from the study.

The patient will be administered epinephrine injection and dexamethasone infusion if hypersensitivity reaction is observed and then the patient should be placed on a monitor immediately and the ICU should be alerted for possible transfer if needed.

For prophylaxis of flu-like symptoms, a dose of 25 mg indomethacin or a comparable dose of nonsteroidal anti-inflammatory drugs (i.e, 600 mg ibuprofen, 500 mg naproxen sodium) may be administered 2 hours before and 8 hours after the start of each dose of study treatments(s) infusion. Alternative treatments for fever (e.g, paracetamol) may be given to patients at the discretion of the investigator.

- - 1. Immune-Related Adverse Events

The immune-related AEs are of special interest in this study. If the events listed below or similar events occur, the investigator should exclude alternative explanations (e.g, combination drugs, infectious disease, metabolic, toxin, disease progression or other neoplastic causes) with appropriate diagnostic tests, which may include but is not limited to serologic, immunologic, and histologic (biopsy) data. If alternative causes has been ruled out; the AE requires the use of systemic steroids, other immunosuppressants, or endocrine therapy; and is consistent with an immune-related mechanism of action, the irAE indicator on the eCRF AE page should be checked. Recommendation for diagnostic evaluation and management of irAEs is based on European Society for Medical Oncology (ESMO)**^55^** and American Society of Clinical Oncology (ASCO)**^56^** guidelines, and common immune-related toxicities are detailed in **Appendix 10**.

A list of potential irAEs is shown below in **Table 15**. All conditions similar to those listed should be evaluated in patients receiving study treatment(s) to determine whether they are irAEs. Recommended diagnostic tests for selected AEs are detailed in [**Appendix 10**](#附录10).

For any adverse events not included in [**Appendix 10**](#附录10), please refer to the ASCO Clinical Practice Guideline**^56^** for further guidance on diagnostic evaluation and management of immune‑related toxicities.

Table 15: Examples of Immune-Related Adverse Events

| **Body system affected** | **Events** |
| --- | --- |
| Skin (mild to common): | Pruritus or rash maculo-papular; vitiligo |
| Skin (moderate): | Follicular or urticarial dermatitis; erythematous/lichenoid rash; Sweet’s syndrome |
| Skin (severe to rare): | Full-thickness necrolysis/Stevens-Johnson syndrome |
| Gastrointestinal: | Colitis (includes diarrhea with abdominal pain or endoscopic/radiographic evidence); diarrhea; pancreatitis; hepatitis; aminotransferase (ALT/AST) elevation; bowel perforation |
| Endocrine: | Thyroiditis, hypothyroidism, hyperthyroidism; hypophysitis with features of hypopituitarism, e.g, fatigue, weakness, weight gain; insulin-dependent diabetes mellitus; diabetic ketoacidosis; adrenal insufficiency |
| Respiratory system: | Pneumonitis/diffuse alveolitis |
| Eye: | Episcleritis; conjunctivitis; iritis/uveitis |
| Neuromuscular: | Arthritis; arthralgia; myalgia; neuropathy; Guillain-Barrè syndrome; meningitis aseptic; myasthenic syndrome/myasthenia gravis, meningoencephalitis; myositis |
| Blood: | Anemia; leukopenia; thrombocytopenia |
| Kidney: | Interstitial nephritis; glomerulonephritis; acute renal failure |
| Cardiac: | Pericarditis; myocarditis; heart failure |

- - 1. Abnormal Kidney Function

When a hepatic event, such as liver function laboratory abnormalities, is observed, the investigator must evaluate for re-activation of viral hepatitis, consider other drug-related toxicities, and exclude progressive disease involving the liver. For diagnosis and management of patients with AST or ALT values ≤ Grade 1 at baseline, please refer to [**Appendix 10**](#附录10).

In patients with Grade 2 AST/ALT abnormalities at baseline, elevated levels of AST and ALT may require therapeutic intervention with steroids. The following algorithm is proposed for the use of steroid treatment:

- Initiate oral prednisolone 1 mg/kg/d and taper over at least 2 to 4 weeks if AST or ALT increases ≥ 50% from baseline for at least one week; if liver function test results worsen, re-escalate the steroid dose as clinically judged (manage as per **Appendix 10**). Study treatment should be withheld until the elevated AST/ALT event resolves or returns to baseline and prednisolone is tapered to ≤ 10 mg.
- If any ALT or AST increases meet Grade 3 or Grade 4 criteria, initiate steroid therapy per the instructions of **Appendix 10**. Study treatment will be permanently discontinued. Study treatment is resumed only after discussion with the study sponsor.

**Abnormal Kidney Function**

The following rules are proposed for steroids that are used to manage immune-related adverse events:

- If the serum creatinine is normal at baseline, refer to **Appendix 10** for diagnosis and management of patients with abnormal kidney functions.
- If the serum creatinine is Grade 1 at baseline and the increase in serum creatinine meets criteria for serum creatinine increase ≥ Grade 2 post study treatment(s), refer to **Appendix 10** for the diagnosis and management of patients with abnormal renal laboratory values. Check the eGFR using the **Appendix 8** and the eGFR calculator link. In the setting of a Grade 2 serum creatinine increase only, study treatment can continue unless the serum creatinine increases by at least 50% from the baseline OR the eGFR falls below 20 mL/min.

Statistical Methods and Sample Size Determination

The statistical analyses will be performed by the sponsor or designee after the study is completed and the database is locked and released. Details of the statistical analyses will be included in a separate Statistical Analysis Plan (SAP).

The following descriptive statistics will be used to summarize the study data on the basis of their nature unless otherwise specified:

• Continuous variables: number of non-missing observed values, mean, standard deviation, median, minimum, and maximum

• Categorical variables: frequencies and percentages

• Time-event variables: the number of non-missing observations (N), median, minimum, and maximum, and Kaplan-Meier event-free rates may also be provided if applicable for specific time-to-event variables

- 1. Statistical Analysis
     1. Analysis Sets

The Safety Analysis Set (SAF) includes all patients who have received ≥ 1 dose of study treatment (any component for the combination therapy).

The Efficacy-Evaluable Analysis Set (EFF) includes patients who have measurable disease per RECIST v1.1 and who have at least 1 evaluable post-baseline tumor assessment unless treatment has been discontinued due to clinical disease progression or death before the first post-treatment tumor assessment.

- - 1. Patients Disposition

The number of patients treated and discontinued from study treatments and/or study and those with major protocol deviations will be counted. The primary reason for study treatment and/or study discontinuation will be summarized according to the categories on the eCRF. The end of study status (alive, dead, withdraw consent, or loss to follow‑up) at the cutoff date will be summarized using the data from the eCRF.

Major protocol deviations will be summarized and listed by each category.

- - 1. Demographics and Other Baseline Characteristics

Demographics and other baseline characteristics will be summarized in the Safety Analysis Set using descriptive statistics. Continuous variables include age, body weight, vital signs, time since initial cancer diagnosis, and time since advanced/metastatic disease diagnosis; categorical variables include number of prior systemic therapies received, gender, ECOG status, country, race and metastatic sites.

- - 1. Prior and Concomitant Medications

Concomitant medications will be coded using the World Health Organization Drug Dictionary. Concomitant medications will be further coded to the appropriate anatomical therapeutic chemical (ATC) code indicating therapeutic classification. Prior and concomitant medications will be summarized and listed by drug and drug class in the clinical study report (CSR) for this protocol. Prior medications will be defined as medications stopped before the first dose of study treatment. Concomitant medications will be defined as medications that (1) started before the first dose of study treatment and are continuing at the time of the first dose of study treatment, or (2) started on or after the date of the first dose of study treatment up to 30 days after the patient’s last dose. A listing of prior and concomitant medications will be included in the CSR of this study protocol.

- 1. Efficacy Analysis
     1. Primary Efficacy Analysis

The primary endpoint ORR will be based upon the investigator’s tumor assessments per RECIST v1.1 from the Efficacy Analysis Set. The targeted ORR for the combination regimen (GP in combination with tislelizumab and ociperlimab) is 45%, whereas the historical control ORR for gemcitabine plus cisplatin in a similar population was 25%. The null and alternative hypotheses are thus set as follows:

H0: ORR = 25%

Ha: ORR > 25%

A binomial exact test will be performed for hypothesis testing. If the obtained one-sided p-value is ≤ 0.05, it is to be concluded that the investigational products statistically significantly increases ORR compared with historical control, and thus demonstrating superiority of the investigational products.

The primary efficacy analysis will be conducted approximately 12 months after the enrollment of the last patient and will be based on the Efficacy Analysis Set.

- - 1. Secondary Efficacy Analyses

The secondary efficacy analyses include DCR, DoR, PFS, PFS rate, OS, and OS rate as assessed by the investigator per RECIST v1.1.

Time-to-event variables, including PFS, OS, and DoR, will be estimated using the Kaplan-Meier (KM) method, with Kaplan-Meier curves generated and plotted over time. The medians of PFS, OS and DoR along with their 95% CIs (constructed using Brookmeyer and Crowley method**^57^** (if estimable) will be estimated based on the investigator’s assessment per RECIST v1.1. Meanwhile, landmark rates at selected time points (6 months and 12 months) for PFS and OS will be estimated using the Kaplan-Meier method with the corresponding 95% CIs constructed using Greenwood’s formula**^58^**. The binomial exact 95% CIs will be used for assessing DCR within the Efficacy Analysis Set.

- - 1. Exploratory Efficacy Analysis

The correlation between expression levels of potential biomarkers programmed cell death ligand-1 (PD-L1) and T-cell immunoglobulin and ITIM domain (TIGIT) with disease status, response to/prognosis of GP in combination with tislelizumab and ociperlimab as first-line treatment for unresectable advanced BTC will be analyzed using multivariate regression analysis in the Efficacy Analysis Set.

- 1. Safety Analysis

Safety will be evaluated for all patients exposed to (or start to receive) any study treatment (any component of the combination therapy). Safety will be determined by the spontaneous reporting of AEs and by laboratory values (hematology, clinical chemistry, coagulation, and urinalysis). Meanwhile, vital signs, physical examination results, and ECG findings will also be used for determining the safety profile. The severity of AEs will be graded according to the NCI-CTCAE v5.0. The incidence of DLT events and TEAEs will be reported as the number (percentage) of patients with TEAEs by system organ class (SOC) and preferred term (PT). Descriptive summary statistics (e.g, n, mean, standard deviation, median, minimum, maximum for continuous variables; n [%] for categorical variables) for observed laboratory parameters, vital signs and their changes from baseline will be calculated.

- - 1. Extent of Exposure

Extent of exposure to each study treatment will be summarized descriptively as the number of cycles received (number and percentage of patients), duration of exposure (days), cumulative total dose received per patient (mg), dose intensity, and relative dose intensity.

The number (percentage) of patients requiring dose reduction, interruption, dose delay, and drug discontinuation due to AEs or other reasons will be summarized for each study treatment.

Patient data listings will be provided for all dosing records and for calculated summary statistics.

- - 1. Adverse Events

The AE verbatim descriptions (investigator’s description from the eCRF) will be coded using Medical Dictionary for Regulatory Activities (MedDRA). AEs will be coded to MedDRA (Version 24.0 or higher) by the preferred term, and primary system organ class.

A treatment-emergent adverse event (TEAE) is defined as any AE or SAE that has an onset date or a worsening in severity from baseline (pretreatment) on or after the date of first dose of study treatment up to 30 days following study treatment discontinuation (Safety Follow-up Visit) or initiation of a new anticancer therapy, whichever occurs first. TEAE classification also applies to irAEs recorded up to 90 days after the last dose of study treatment or until the initiation of new anticancer therapy, whichever occurs first. Only TEAEs will be included in summary tables. All AEs, treatment-emergent or otherwise, will be presented in patient data listings.

The incidence of TEAEs will be reported as the number (percentage) of patients with TEAEs by SOC and preferred term. A patient is counted only once by the highest severity grade per NCI‑CTCAE v5.0, within an SOC and preferred term, even if the patient experiences more than 1 TEAE within a specific SOC and preferred term. The number (percentage) of patients with TEAEs will also be summarized by relationship to the study treatment(s). All SAEs, deaths, ≥ Grade 3 TEAEs, irAEs, treatment-related TEAEs, and TEAEs leading to treatment discontinuation, dose interruption, or dose delay will be summarized.

- - 1. Laboratory Analyses

Clinical laboratory (e.g, hematology, serum chemistry) values will be evaluated as appropriate. Abnormal laboratory values will be flagged and identified as those outside of (above or below) the normal range. Reference (normal) ranges for laboratory parameters will be included in the CSR for this study protocol. Descriptive summary statistics (e.g, n, mean, standard deviation, median, minimum, and maximum for continuous variables; n [%] for categorical variables) for laboratory parameters and their changes from baseline will be calculated. Laboratory values will be summarized by visit and by the worst post-baseline visit.

Laboratory parameters that are graded in NCI-CTCAE v5.0 will be summarized by NCI‑CTCAE Grade. In the summary of laboratory parameters by the NCI-CTCAE Grade, parameters with NCI-CTCAE grading in both high and low directions will be summarized separately.

- - 1. Vital Signs

Specific vital signs (e.g, blood pressure and temperature) will be summarized, including changes from baseline, and listed.

- 1. Sample Size Consideration

The study plans to enroll 45 patients.

Based on the the exact binomial distribution method adopted for this single-arm trial, the sample size of 36 patients will provide 80% statistical power to demonstrate statistically significant difference between the ORR of combination regimen (assumed to be 45%, as assessed per RECIST v1.1) and the historical control ORR of 25% at a one-sided alpha level of 0.05. Assuming a 20% drop-out rate, a total of 45 patients will be enrolled.

Source Documents and Access to Source Data/Documents

The investigator must maintain adequate and accurate records to enable the conduct of the study to be fully documented. Such records include, but are not limited to, the protocol, protocol amendments, ICFs, and documentation of IRB/IEC and governmental approvals. In addition, at the end of the study, the investigator will receive the patient data, which include an audit trail containing a complete record of all changes to data.

- 1. Access to Information for Monitoring

In accordance with International Conference on Harmonisation (ICH) GCP guidelines, the study monitor must have direct access to the investigator’s source documentation to verify the data recorded in the eCRFs for consistency.

The monitor is responsible for routine review of the eCRFs at regular intervals throughout the study to verify adherence to the protocol and the completeness, consistency, and accuracy of the data being entered on them. The monitor should have access to any patient records needed to verify the entries on the eCRFs. The investigator agrees to cooperate with the monitor to ensure that any problems detected during these monitoring visits are resolved.

- 1. Access to Information for Audit or Inspection

Representatives of regulatory authorities or of the sponsor may conduct inspections or audits any time during or after completion of this clinical study. If the investigator is notified of an inspection by a regulatory authority, the investigator agrees to notify the sponsor or its designee immediately. The investigator agrees to provide to representatives of a regulatory agency or sponsor access to records, facilities, and personnel for the effective conduct of any inspection or audit.

Quality Assurance and Quality Control

- 1. Quality Assurance

To ensure compliance with Good Clinical Practice (GCP) and all applicable regulatory requirements, the sponsor may conduct a quality assurance audit. Regulatory agencies may also conduct a regulatory inspection of this study. Such audits/inspections could occur at any time during or after completion of the study. If an audit or inspection occurs, the investigator and institution agree to allow the auditor or inspector direct access to all relevant documents and to allocate their time and the time of their personnel to the auditor or inspector to discuss findings and any relevant issues.

- 1. Site Inspection

This study will be organized, performed, and reported in compliance with the protocol, standard operating procedures (SOPs), working practice documents, and applicable regulations and guidelines.

Site visits will be conducted by the sponsor or an authorized representative to inspect study data, patients’ medical records, and eCRFs. The investigator is to permit national and local health authorities; sponsor’s study monitors, representatives, and collaborators; and IRB/IEC members to inspect study sites and check all records relevant to this study.

- 1. Drug Accountability

The investigator or designee (e.g, pharmacist) is responsible for ensuring adequate accountability of all used and unused study treatments. This includes acknowledgment of receipt of each shipment of study treatment(s) (quantity and condition), patient drug dispensation records, and returned or destroyed study treatment(s). Dispensation records will document the quantities of study treatments and quantities that are dispensed to patients, including the batch number, date dispensed, patient identifier number, patient initials, and the initials of the person dispensing the medication.

At study site initiation, the monitor will evaluate the study site’s standard operating procedure for study treatment disposal/destruction in order to ensure that it complies with requirements of the clinical study. At the end of the study, following final drug inventory reconciliation by the monitor, the study site will dispose of and/or destroy all unused study treatment supplies, including empty containers, according to these procedures. If the study site cannot meet sponsor’s requirements for disposal, arrangements will be made between the study site and sponsor or its representative for destruction or return of unused and returned study treatment supplies.

All drug supplies and associated documentation will be periodically reviewed and verified by the study monitor over the course of the study.

Ethics/Protection of Human Patients

- 1. Ethical Standard

The study will be conducted in full conformance with the ICH E6 guideline for Good Clinical Practice (GCP) and the principles of the Declaration of Helsinki or the laws and regulations of the country in which the research is conducted, whichever affords the greater protection to the individual. The study will comply with the requirements of the ICH E2A guideline (Clinical Safety Data Management: Definitions and Standards for Expedited Reporting).

- 1. Institutional Review Board/Independent Ethics Committee

This protocol, the ICFs, any information to be given to the patient, and relevant supporting information must be submitted to the IRB/IEC by the principal investigator and reviewed and approved by the IRB/IEC before the study is initiated. In addition, any patient recruitment materials must be approved by the IRB/IEC.

The principal investigator is responsible for providing written summaries of the status of the study to the IRB/IEC annually or more frequently in accordance with the requirements, policies, and procedures established by the IRB/IEC. The investigator is also responsible for promptly informing the IRB/IEC of any protocol amendments. The investigator must report all AEs to the sponsor and also report SAEs to local health authorities and the IRB/IEC according to SAE reporting requirements. The investigators is responsible for ensuring that such reports are reviewed and processed in accordance with health authority requirements and the policies and procedures established by their IRB/IEC and archived in the site’s study file.

- 1. Protocol Modifications

All protocol amendments will be prepared by the study sponsor. All protocol modifications must be submitted to competent authorities according to local requirements and to the IRB/IEC together with a revised model ICF in accordance with local requirements. Written documentation from competent authorities (according to local requirements) and from the IRB or IEC must be obtained by the investigator before changes can be implemented, except for changes necessary to eliminate an immediate hazard to patients or changes that involve logistical or administrative aspects only (e.g, change in medical monitor or contact information).

Information on any change in risk and/or change in scope must be provided to patients already actively participating in the study, and they must read, understand and sign each revised ICF confirming their willingness to remain in the study.

- 1. Informed Consent

The sponsor’s sample ICFs will be provided to each site. If applicable, it will be provided in a certified translation of the local language. The final IRB/IEC-approved ICFs must be provided to the sponsor for health authority submission purposes according to local requirements.

The ICFs must be signed and dated by the patient or the patient’s legally authorized representative before his or her participation in the study. Written ICFs will be obtained before the patient can participate in the study.

The ICFs will be revised whenever there are changes to study procedures or when new information becomes available that may affect the willingness of the patient to participate. The final revised IRB-/IEC-approved ICFs must be provided to the sponsor for health authority submission purposes.

Patients must re-consent to the most current version of the ICFs (or to a significant new information/findings addendum in accordance with applicable laws and IRB/IEC policy) during their participation in the study. For any updated or revised ICFs, the case history or clinical records for each patient shall document the informed consent process and that written informed consent is re-obtained using the updated/revised ICFs for continued participation in the study.

A copy of each signed ICF must be provided to the patient or the patient’s legally authorized representative. All signed and dated ICFs must remain in each patient’s study file or in the site file and must be available for verification by study monitors at any time.

- 1. Patient and Data Confidentiality

All records regarding patient identifiers will be kept confidential and will not be made publicly available to the extent permitted by relevant laws and/or regulations. Only study personnel, such as the investigator and the study nurse know patient identity. Electronic Case Report Form (eCRF) does not include patients’ names. Only the patient identifier number and initials will be recorded in the eCRF. If other documents (such as pathology report) include patient’s name, the name must be redacted on the copies. The computerized study reports must comply with local data protection laws. The patient’s identity will also be kept confidential when publishing study results.

The investigator will maintain a record that can identify patients.

Data Handling and Records Retention

- 1. Data Collection and Management Responsibilities
     1. Data Collection

Data will be captured in the EDC system.

Data collection in the eCRF should follow the instructions described in the eCRF completion guidelines. The investigator has the ultimate responsibility for the collection and reporting of all clinical data entered in the eCRF. The investigator or designee must sign the completed casebooks to attest to its accuracy, authenticity, and completeness.

Data contained in the eCRFs are the joint property of sponsor and funding party and should not be made available in any form to third parties without written permission from sponsor, except for authorized representatives of sponsor or appropriate regulatory authorities.

- - 1. Data Management/Coding

All final patient data, both eCRF and external data (e.g, laboratory data), collected according to the protocol will be stored by the sponsor at the end of the study.

Standard procedures (including following data review guidelines, computerized validation to produce queries and maintenance of an audit file which includes all database modifications) will be followed to support accurate data collection. Data will be reviewed for outliers, logic, data inconsistencies and completeness.

During the course of the study, a study monitor (CRA) will make site visits to review protocol compliance, compare eCRFs against individual patient’s medical records, and ensure that the study is being conducted according to pertinent regulatory requirements.

The eCRF entries will be verified against source documentation. The review of medical records will be performed in a manner to ensure that patient confidentiality is maintained. Checking the eCRFs for completeness and clarity and cross-checking with source documents is required to monitor the progress of the study. Direct access to source data is also required for inspections and audits and will be carried out giving due consideration to data protection and medical confidentiality.

Adverse events will be coded using the MedDRA Version 24.0 or higher. Concomitant medications will be coded using the World Health Organization Drug Dictionary. Comorbidities/medical history will be coded using the MedDRA Version 24.0 or higher.

- 1. Data Integrity

Due to the open‑label design of the study, access to the EDC system will be assigned only to predefined study personnel. Although the study is open-label, analyses or summaries generated from actual treatment received will be restricted and documented.

- 1. Study Records Retention

The investigator must maintain adequate and accurate records to enable the conduct of the study to be fully documented, and to allow the study data to be subject to subsequent verification. These documents should be classified into at least the following 2 categories: 1) the investigator’s study file and 2) the patient clinical source documents.

The investigator’s study file will contain the protocol/amendments, blank eCRF and query forms, IRB/IEC and governmental approval with correspondence, informed consent forms, drug records, staff curriculum vitae and authorization forms, and other appropriate documents and correspondence.

Patient clinical source documents (usually defined by the project in advance to record key efficacy/safety parameters independent of the eCRFs) would include (although not be limited to) the following: patient hospital/clinic records, physician’s and nurse’s notes, appointment book, original laboratory reports, ECGs, X‑ray, pathology and special assessment reports, consultant letters, and screening and enrollment log.

Following closure of the study, the investigator must maintain all study records in a safe and secure location. The records must be maintained to allow easy and timely retrieval, when needed (e.g, audit or inspection), and, whenever feasible, to allow any subsequent review of data in conjunction with assessment of the facility, supporting systems, and personnel. Where permitted by local laws/regulations or institutional policy, some or all of these records can be maintained in a format other than hard copy (e.g, microfiche, scanned, electronic); however, caution needs to be exercised before such action is taken. The investigator must assure that all reproductions are legible, are a true and accurate copy of the original, and meet accessibility and retrieval standards, including regenerating a hard copy, if required. Furthermore, the sponsor must ensure there is an acceptable backup of these reproductions and that an acceptable quality control process exists for making these reproductions.

The sponsor will inform the investigator of the time period for retaining these records to comply with all applicable regulatory requirements. The minimum retention time will meet the strictest standard applicable to that study site for the study, as dictated by any institutional requirements or local laws or regulations, or the sponsor’s standards/procedures; otherwise, the retention period will default to 5 years.

The investigator must notify the sponsor of any changes in the archival arrangements, including but not limited to the following: archival at an off‑site facility, transfer of ownership of the records in the event the investigator leaves the study site.

- 1. Protocol Deviations

The investigator is responsible for ensuring that the study is conducted in accordance with the procedures and evaluations described in this protocol. Investigators assert they will apply due diligence to avoid protocol deviations.

The investigator is to document and explain any deviations from the approved protocol. The investigator must promptly report any major deviations that might impact patient safety and/or data integrity to the sponsor and to the IRB/IEC, in accordance with established IRB/IEC policies and procedures.

- 1. Publication and Data Sharing Policy

The results of this study will be published or presented at scientific meetings in a timely, objective, and clinically meaningful manner that is consistent with good science, industry and regulator guidance, and the need to protect the intellectual property of the sponsor, regardless of the outcome of the study. The data that are generated in this clinical study are the exclusive property of the sponsor and are confidential. As for multicenter study, the first publication or disclosure of study results shall be a complete, joint, multicenter publication or disclosure coordinated by the sponsor. Any subsequent publications will be referenced to the initial publication. Authorship will be determined by mutual agreement and all authors must meet the criteria for authorship established by the International Committee of Medical Journal Editors (ICMJE).

After conclusion of the study and without prior written approval from the sponsor, investigators in this study must only communicate, orally present, or publish in scientific journals or other scholarly media after the following conditions have been met:

- Results of the study in their entirety have been publicly disclosed by or with the consent of the sponsor in an abstract, manuscript, or presentation form; or
- The study has been completed at all study sites for ≥ 2 years.
- Each investigator agrees to submit all manuscripts or congress abstracts and posters/presentations to the sponsor for review prior to submission. This allows the sponsor to protect proprietary information, provide comments based on information from other studies that may not yet be available to the investigator, and ensure scientific and clinical accuracy. The details of the processes of producing and reviewing reports, manuscripts, and presentations based on the data from this study will be presented in the investigator’s clinical study agreement.
  1. Study and Study Site Closure

Upon completion of the study, the monitor will conduct the following activities in conjunction with the investigator or study site personnel, as appropriate:

- Return of all study data to the sponsor.
- Resolve and close all data queries.
- Accountability, reconciliation, and arrangements for unused study treatment(s).
- Review of study records for completeness
- Return of treatment codes to the sponsor.

In addition, the sponsor reserves the right to temporarily suspend or prematurely discontinue this study either at a single study site or at all study sites at any time for reasons including, but not limited to, safety or ethical issues or severe noncompliance. If the sponsor determines that such action is needed, the sponsor will discuss this with the investigator (including the reasons for taking such action) at that time. When feasible, the sponsor will provide advance notification to the investigator of the impending action before it taking effect.

The sponsor will promptly inform all other investigators and/or institutions conducting the study if the study is suspended or terminated and will also inform the regulatory authorities of the suspension or termination of the study and the reason(s) for the action. If required by applicable regulations, the investigator must inform the IRB/IEC promptly and provide the reason(s) for the suspension or termination.

If the study is prematurely discontinued, all study data must be returned to the sponsor. In addition, arrangements will be made for the return of all unused study treatment(s) in accordance with the applicable sponsor procedures for the study.

Financial compensation to investigators and/or institutions will be in accordance with the agreement established between the investigators and the sponsor.

- 1. Information Disclosure and Inventions

The principal investigator at each site must comply with all the terms, conditions, and responsibilities stipulated in the study agreement. For the ownership of intellectual property rights and related matters, the provisions of the “Investigator Initiated Study Agreement” should be followed if there are conflicts between the study agreements.

References

1, Massarweh NN, El-Serag HB. Epidemiology of Hepatocellular Carcinoma and Intrahepatic Cholangiocarcinoma. Cancer Control. 2017 Jul-Sep;24(3):1073274817729245.

2, Global Burden of Disease Cancer Collaboration. JAMA Oncol. 1, 505–527 (2015).

3, Andia ME, Hsing AW, Andreotti G, Ferreccio C. Geographic variation of gallbladder cancer mortality and risk factors in Chile: a population-based ecologic study. Int J Cancer. 2008 Sep 15;123(6):1411-6.

4, Randi G, Franceschi S, La Vecchia C. Gallbladder cancer worldwide: geographical distribution and risk factors. Int J Cancer. 2006 Apr 1;118(7):1591-602.

5, Chen W, CA Cancer J Clin, 2016;66:115–132

6, Marcano-Bonilla L, Mohamed EA, Mounajjed T, Roberts LR. Biliary tract cancers: epidemiology, molecular pathogenesis and genetic risk associations. Chin Clin Oncol. 2016 Oct;5(5):61.

7, Patel T. Worldwide trends in mortality from biliary tract malignancies. BMC Cancer. 2002 May 3;2:10.

8, Razumilava N, Gores GJ. Cholangiocarcinoma[J]. Lancet, 2014,383(9935):2168‐2179.

9, Rizvi S, Khan SA, Hallemeier CL, et al. Cholangiocar ‐ cinoma‐evolving concepts and therapeutic strategies[J]. Nat Rev Clin Oncol, 2018, 15(2): 95-111.

10, Kelley RK, Bridgewa ter J, Gores G J, e t al. Sys temic therapies for intrahepatic cholangiocarcinoma[J]. J Hepatol, 2020,72(2):353-363.

11, Jarnagin WR, Shoup M. Surgical management of cholangiocarcinoma. Semin Liver Dis. 2004 May;24(2):189-99.

12, Goetze TO. Gallbladder carcinoma: Prognostic factors and therapeutic options.World J Gastroenterol. 2015 Nov 21;21(43):12211-7.

13, Raderer M, Hejna MH, Valencak JB, Kornek GV, Weinländer GS, Bareck E, Lenauer J, Brodowicz T, Lang F, Scheithauer W. Two consecutive phase II studies of 5-fluorouracil/leucovorin/mitomycin C and of gemcitabine in patients with advanced biliary cancer. Oncology. 1999 Apr;56(3):177-80.

14, Valle J, Wasan H, Palmer DH, Cunningham D, Anthoney A, Maraveyas A, Madhusudan S, Iveson T, Hughes S, Pereira SP, Roughton M, Bridgewater J; ABC-02 Trial Investigators. Cisplatin plus gemcitabine versus gemcitabine for biliary tract cancer. N Engl J Med. 2010 Apr 8;362(14):1273-81.

15, Okusaka T, Nakachi K, Fukutomi A, Mizuno N, Ohkawa S, Funakoshi A, Nagino M, Kondo S, Nagaoka S, Funai J, Koshiji M, Nambu Y, Furuse J, Miyazaki M, Nimura Y. Gemcitabine alone or in combination with cisplatin in patients with biliary tract cancer: a comparative multicentre study in Japan. Br J Cancer. 2010 Aug 10;103(4):469-74.

16, NCCN Hepatobiliary tumor Clinical Practice Guideline 2021 V2

17, 2020 Biliary Malignant Tumours Diagnosis and Treatment guideline Chinese Society of Clinical Oncology (CSCO)

18, Mizusawa J, Morizane C, Okusaka T, Katayama H, Ishii H, Fukuda H, Furuse J;Hepatobiliary and Pancreatic Oncology Group of the Japan Clinical Oncology Group. Randomized Phase III study of gemcitabine plus S-1 versus gemcitabine plus cisplatin in advanced biliary tract cancer: Japan Clinical Oncology Group Study (JCOG1113, FUGA-BT). Jpn J Clin Oncol. 2016 Apr;46(4):385-8.

19, Kim ST, Kang JH, Lee J, et al. Capecitabine plus oxaliplatin versus gemcitabine plus oxaliplatin as first-line therapy for advanced biliary tract cancers: a multicenter, open-label, randomized, phase Ⅲ , noninferiority trial[J]. Ann Oncol,2019,30(5):788-795.

20, Von Hoff DD, Ramanathan RK, Borad MJ, Laheru DA, Smith LS, Wood TE, Korn RL, Desai N, Trieu V, Iglesias JL, Zhang H, Soon-Shiong P, Shi T, Rajeshkumar NV, Maitra A, Hidalgo M. Gemcitabine plus nab-paclitaxel is an active regimen in patients with advanced pancreatic cancer: a phase I/II trial. J Clin Oncol. 2011 Dec 1;29(34):4548-54.

21, Mizrahi JD, Gunchick V, Mody K, Xiao L, Surapaneni P, Shroff RT, Sahai V. Multi-institutional retrospective analysis of FOLFIRI in patients with advanced biliary tract cancers. World J Gastrointest Oncol. 2020 Jan 15;12(1):83-91.

22, Kanai M, Hatano E, Kobayashi S, Fujiwara Y, Marubashi S, Miyamoto A, Shiomi H, Kubo S, Ikuta S, Yanagimoto H, Terajima H, Ikoma H, Sakai D, Kodama Y, Seo S, Morita S, Ajiki T, Nagano H, Ioka T. A multi-institution phase II study of gemcitabine/cisplatin/S-1 (GCS) combination chemotherapy for patients with advanced biliary tract cancer (KHBO 1002). Cancer Chemother Pharmacol. 2015 Feb;75(2):293-300.

23, Shroff RT, Javle MM, Xiao L, Kaseb AO, Varadhachary GR, Wolff RA, Raghav KPS, Iwasaki M, Masci P, Ramanathan RK, Ahn DH, Bekaii-Saab TS, Borad MJ. Gemcitabine, Cisplatin, and nab-Paclitaxel for the Treatment of Advanced Biliary Tract Cancers: A Phase 2 Clinical Trial. JAMA Oncol. 2019 Jun 1;5(6):824-830.

24, Lamarca A, Palmer DH, Wasan HS, et al. Advanced Biliary Cancer Working Group. Second-line FOLFOX chemotherapy versus active symptom control for advanced biliary tract cancer (ABC-06): a phase 3, open-label, randomised, controlled trial. Lancet Oncol. 2021 May;22(5):690-701.

25, https://clinicaltrials.gov/ (up to May 2021)

26, Loeuillard E, Conboy CB, Gores GJ, et al. Immunobiology of cholangiocarcinoma[J]. JHEP Rep, 2019, 1(4): 297-311.

27, Robinson MW, Harmon C, O′Farrelly C. Liver immunology and its role in inflammation and homeostasis[J]. Cell Mol Immunol,2016,13(3):267-276. DOI:10.1038/cmi.2016.3.

28, Razumilava N, Gores GJ. Cholangiocarcinoma[J]. Lancet, 2014,383(9935):2168‐2179.

29, Kasper HU, Drebber U, Stippel DL, et al. Liver tumor infiltrating lymphocytes: comparison of hepatocellular and cholangiolar carcinoma[J]. World J Gastroenterol, 2009, 15(40):5053-5057.

30, Ott PA, Bang YJ, Piha-Paul SA, Razak ARA, Bennouna J, Soria JC, Rugo HS, Cohen RB, O'Neil BH, Mehnert JM, Lopez J, Doi T, van Brummelen EMJ, Cristescu R, Yang P, Emancipator K, Stein K, Ayers M, Joe AK, Lunceford JK. T-Cell-Inflamed Gene-Expression Profile, Programmed Death Ligand 1 Expression, and Tumor Mutational Burden Predict Efficacy in Patients Treated With Pembrolizumab Across 20 Cancers: KEYNOTE-028. J Clin Oncol. 2019 Feb 1;37(4):318-327.

31, Piha-Paul SA, Oh DY, Ueno M, Malka D, Chung HC, Nagrial A, Kelley RK, Ros W, Italiano A, Nakagawa K, Rugo HS, de Braud F, Varga AI, Hansen A, Wang H, Krishnan S, Norwood KG, Doi T. Efficacy and safety of pembrolizumab for the treatment of advanced biliary cancer: Results from the KEYNOTE-158 and KEYNOTE-028 studies. Int J Cancer. 2020 Oct 15;147(8):2190-2198.

32, Kim RD, Chung V, Alese OB, et al. A phase 2 multi-institutional study of nivolumab for patients with advanced refractory biliary tract cancer[J]. JAMA Oncol, 2020, 6(6): 888-894.

33, Ueno M, Ikeda M, Morizane C, et al. Nivolumab alone or in combination with cisplatin plus gemcitabine in Japanese patients with unresectable or recurrent biliary tract cancer: a non-randomised, multicentre, open-label, phase 1 study[J]. Lancet Gastroenterol Hepatol, 2019, 4(8): 611- 621.

34, Koido S, Kan S, Yoshida K, et al. Immunogenic modulation of cholangiocarcinoma cells by chemoimmunotherapy[J]. Anticancer Res,2014, 34(11):6353-6361.

35, Bhatt DR, White R, Martin G, et al. Transitional hypothermia in preterm newborns[J]. Adv Neonatal Care,2010, 10(5 Suppl):S15-S17. DOI:10.1097/ANC.0b013e3181ef 7dfd.

36, Oh DY, et al. 2020 ASCO abstract 4520

37, Chen X, Wu X, Wu H, et al. Camrelizumab plus gemcitabine and oxaliplatin (GEMOX) in patients with advanced biliary tract cancer: a single-arm, open-label, phase II trial. Journal for ImmunoTherapy of Cancer 2020;8:e001240.

38, Wei Li. et al. 2021 ASCO Abstract e16170

39, J Zhou.et al. 2021 ASCO Abstract 4094

40, Ioka T, Ueno M, Oh DY, et al. Evaluation of safety and tolerability of durvalumab (D) with or without tremelimumab (T) in patients (pts) with biliary tract cancer (BTC) [J]. J Clin Oncol,2019,37(4_Suppl):387.

41, Luis Villanueva.et al. 2021 ASCO Abstract 4080

42, Duffy AG, Ulahannan SV, Makorova-Rusher O, et al. Tremelimumab in combination with ablation in patients with advanced hepatocellular carcinoma[J]. J Hepatol, 2017,66(3):545-551.

43: Labrijn AF, et al. Therapeutic IgG4 antibodies engage in Fab-arm exchange with endogenous human IgG4 in vivo. Nature Biotechnology.2009; 27(8):767-771.

44: Tong zhang, et al. (2018) The binding of an anti-PD-1 antibody to FcγRI has a profound impact on its biological functions. Immunotherapy 67: 1079-1090.

45, Ociperlimab Investigator’s Brochure

46, Expression and Prognostic Implication of PD-L1 and TIGIT in 297 ICC Patients. Data from Zhongshan Hospital, Fudan University.

47, Di Caro G, Cortese N, Castino GF, Grizzi F, Gavazzi F, Ridolfi C, et al. Dual prognostic significance of tumor-associated macrophages in human pancreatic adenocarcinoma treated or untreated with chemotherapy. Gut. 2016;65(10):1710-1720.

48, Liu WM, Fowler DW, Smith P, Dalgleish AG. Pre-treatment with chemotherapy can enhance the antigenicity and immunogenicity of tumors by promoting adaptive immune responses. Br J Cancer. 2010;102(1):115-123.

49, Lesterhuis WJ, Punt CJ, Hato SV, Eleveld-Trancikova D, Jansen BJ, Nierkens S, et al. Platinum-based drugs disrupt STAT6-mediated suppression of immune responses against cancer in humans and mice. J Clin Invest. 2011;121(8):3100-3108.

50, Chen C, Chen Z, Chen D, Zhang B, Wang Z, Le H. Suppressive effects of gemcitabine plus cisplatin chemotherapy on regulatory T cells in non-small cell lung cancer. J Int Med Res. 2015;43(2):180-187.

51, Mody K, Starr J, Saul M, et al. Patterns and genomic correlates of PD-L1 expression in patients with biliary tract cancers[J]. J Gastrointest Oncol, 2019, 10(6): 1099- 1109.

52, Terrault N A, et al. AASLD guidelines for treatment of chronic hepatitis B. Hepatology.2016;63: 261-283.

53, AASLD/IDSA HCV Guidance Panel. Hepatitis C guidance: AASLD-IDSA recommendations for testing, managing, and treating adults infected with hepatitis C virus. Hepatology. 2015;62: 932-954.

54, UK Anaphylaxis Algorithm Resuscitation Council UK, 2008

55, Haanen JBAG, et al. Management of Toxicities from Immunotherapy: ESMO Clinical Practice Guidelines for Diagnosis, Treatment and Follow-up. Annals of Oncology.2017; 28 (Supplement 4): iv119–iv142, 2017

56, Brahmer JR, et al. Management of Immune-Related Adverse Events in Patients Treated With Immune Checkpoint Inhibitor Therapy: American Society of Clinical Oncology Clinical Practice Guideline. J Clin Oncol. 2018 Jun 10;36(17):1714-1768.

57, Brookmeyer B and Crowley J. A confidence interval for the median survival time, Biometrics 38(1), p29-41, 1982.

58, Greenwood M. The natural duration of cancer. Reports of Public Health and Related Subjects, HMSO, London, 33:1-26.1926.

Appendix 1 Study Procedures

| **Assessment** | **Screening period^1^** | **Treatment period (1 cycle consists of 3 weeks)** | | | | | | | | | | | **End-of-treatment visit^2^** | **Safety follow-up telephone call^3^** | **Survival Follow-up^4^** |
| --- | --- | --- | --- | --- | --- | --- | --- | --- | --- | --- | --- | --- | --- | --- | --- |
|  |  | **Cycle 1** | | **Cycle 2** | | **Cycle 3** | | **Cycle 4** | | **Cycle 5-8** | | **Cycle 9 and onwards** |  |  |  |
| **Days (window)** | **-28d to -1d** | **D1±2** | **D8±2** | **D1±2** | **D8±2** | **D1±2** | **D8±2** | **D1±2** | **D8±2** | **D1±2** | **D8±2** | **D1±2** | **Within 30 days after last dose of study treatment(s)** | **60 and 90 (± 14) days after last dose** | **Every 3 months (± 2 w)** |
| Informed consent | X |  |  |  |  |  |  |  |  |  |  |  |  |  |  |
| Inclusion/exclusion criteria | X |  |  |  |  |  |  |  |  |  |  |  |  |  |  |
| Demographic/medical history/prior medications^5^ | X |  |  |  |  |  |  |  |  |  |  |  |  |  |  |
| Vital signs/height and weight^6^ | X | X | X | X | X | X | X | X | X | X | X | X | X |  |  |
| Physical examination^7^ | X |  |  |  |  |  |  |  |  |  |  |  |  |  |  |
| Symptom-directed physical examination^7^ |  | X | X | X | X | X | X | X | X | X | X | X | X |  |  |
| Child-Pugh classification scoring^8^ | X（-7d to -1d） |  |  |  |  |  |  |  |  |  |  |  |  |  |  |
| ECOG performance status | X | X |  | X |  | X |  | X |  | X |  | X | X |  |  |
| 12-lead ECG^9^ | X | X |  | X |  | X |  | X |  | X |  | X | X |  |  |
| Adverse event^10^ | X | X | X | X | X | X | X | X | X | X | X | X | X | X |  |
| Concomitant medications | X | X | X | X | X | X | X | X | X | X | X | X | X | X |  |
| Hematology^11^ | X（-7d to -1d） | X | X | X | X | X | X | X | X | X | X | X | X |  |  |
| Serum chemistry^11^ | X（-7d to -1d） | X | X | X | X | X | X | X | X | X | X | X | X |  |  |
| CK and CK-MB^11^ | X（-7d to -1d） | X | X | X | X | X | X | X | X | X | X | X | X |  |  |
| Coagulation parameters^11,12^ | X（-7d to -1d） | X | X | X | X | X | X | X | X | X | X | X | X |  |  |
| Urinalysis^11^ | X（-7d to -1d） | X | X | X | X | X | X | X | X | X | X | X | X |  |  |
| Pregnancy test^13^ | X (-7d to -1d) | As clinically indicated | | | | | | | | | | | |  |  |
| Thyroid function^14^ | X |  |  |  |  |  |  | X |  |  |  | X | X |  |  |
| AFP, CEA, CA19-9, CA125^15^ | X |  |  |  |  |  |  | X |  |  |  | X | X |  |  |
| HBV/HCV tests^16^ | X | As clinically indicated | | | | | | | | | | | |  |  |
| HIV/syphilis test | X | As clinically indicated | | | | | | | | | | | |  |  |
| Pulmonary function test^17^ | X | As clinically indicated | | | | | | | | | | | |  |  |
| Tumor imaging monitoring^18^ | X |  |  |  |  |  |  | X |  |  |  | X | X |  |  |
| Tislelizumab administration^19^ |  | X |  | X |  | X |  | X |  | X |  | X |  |  |  |
| Ociperlimab administration^19^ |  | X |  | X |  | X |  | X |  | X |  | X |  |  |  |
| Gemcitabine and csplatin administration^19^ |  | X | X | X | X | X | X | X | X | X | X |  |  |  |  |
| Tumor tissue sample collection (for biomarker testing) ^20^ | X |  |  |  |  |  |  |  |  |  |  |  |  |  |  |
| Survival status |  |  |  |  |  |  |  |  |  |  |  |  |  |  | X |
| New anticancer therapy |  |  |  |  |  |  |  |  |  |  |  |  |  |  | X |

Abbreviations: AE, adverse event; ECG, electrocardiogram; ECOG, Eastern Cooperative Oncology Group; eCRF, electronic case report form; FFPE, formalin-fixed paraffin-embedded; HBcAb, hepatitis B core antibody; HBsAg, hepatitis B surface antigen; HBV, hepatitis B virus; HCV, hepatitis C virus; HBsAb, hepatitis B surface antibody; HIV, human immunodeficiency virus; IEC, Independent Ethics Committee; irAE, immune-related adverse event; IRB, Institutional Review Board; IRC, Independent Review Committee; IV, intravenous; MRI, magnetic resonance imaging; NCI‑CTCAE, National Cancer Institute Common Terminology Criteria for Adverse Events; Q3W, once every 3 weeks; SAE, serious adverse event; TSH, thyroid stimulating hormone; v, version

1. Written informed consent is required prior to performing any study-specific tests or procedures. Results of standard‑of‑care tests or examinations performed before obtaining informed consent and ≤ 28 days prior to start the first dose of study treatment may be used for screening assessments rather than repeating such tests.
2. The End of Treatment (EOT) Visit is conducted within 30 days after last dose of the study treatment(s) or before initiation of a new anticancer treatment, whichever occurs first. If routine laboratory tests (e.g, hematology, serum chemistry) were completed within 7 days before the End of Treatment Visit, these tests need not be repeated. Tumor assessments are not required at the End of Treatment Visit provided that fewer than 9 weeks have passed since the last assessment.
3. Safety follow-up phone call should be conducted to assess irAEs and concomitant medications if appropriate (i.e, associated with an irAE or is a subsequent anticancer therapy) at 60 days (± 14 days) and 90 days (± 14 days) after the last dose of study treatments, respectively, regardless of whether the patient starts a new anticancer therapy.
4. Patients will be followed for survival and to obtain information on subsequent anticancer therapy information after discontinuation of study treatment via telephone calls, patient medical records, and/or clinic visits approximately every 3 months (± 14 days) after the EOT Visit until death, loss to follow-up, withdrawal of consent, or study termination.
5. Including history of treatment for the primary diagnosis, including prior medications, systemic therapy and surgical treatment. Information on radiographic studies performed before study entry may be collected for review by the investigator.
6. Vital signs collected on study include temperature (°C), pulse rate, and blood pressure (systolic and diastolic) while the patient is in a seated position after resting for 10 minutes. Vital signs will be recorded at Screening, during treatment period, and at the EOT Visit. The patient’s vital signs are required to be recorded within 60 minutes before, during, and 30 minutes after the first infusion of the study treatments in the first two cycles. For subsequent infusions, vital signs will be collected within 60 minutes before infusion and, if clinically indicated, during and 30 minutes after the infusion. Height will be recorded at Screening only. Weight will be recorded at Screening, on Day 1 of each cycle, and at the EOT Visit.
7. During the Screening Visit, a complete physical examination will be conducted. At subsequent visits (and as clinically indicated), limited, symptom-directed physical examinations will be performed.
8. Child-Pugh A classification for liver function is required within 7 days of the first dose of study treatment.
9. 12-lead ECGs will be performed for all patients at Screening, before dosing on D1 of each treatment cycle, and at EOT Visit. ECG recordings will be performed after the patient has been resting for at least 10 minutes, and a repeat ECG will be performed to confirm abnormal findings, if any.
10. The AEs and laboratory abnormalities will be graded per NCI-CTCAE v5.0. All adverse events will also be evaluated for seriousness. After informed consent has been signed, but prior to the administration of the study treatment, only SAEs should be reported. After the first dose of study treatment, all AEs and SAEs, regardless of their assessed relationship to study treatment, are to be reported until either 30 days after the last dose of study treatment or the initiation of new anticancer therapy, whichever occurs first. In addition, telephone contacts with patients should be conducted to assess irAEs and concomitant medications (if appropriate, i.e, associated with an irAE or is a new anticancer therapy) at 60 days (± 14 days), and 90 days (± 14 days) after the last dose of study treatment, respectively, regardless of whether or not the patient starts a new anticancer therapy. IrAEs (serious or nonserious) will be reported until 90 days after the last dose of study treatment, regardless of whether or not the patient starts a new anticancer therapy. The investigator should report any SAEs that are assessed as related to the study treatment, at any time after treatment discontinuation.
11. Serum chemistry, CK/CK-MB, hematology, coagulation test and urinalysis will be performed at Screening, during treatment, and at EOT Visit; As for treatment period, they will be performed before administration on Day 1 (D1) and Day 8 (D8) of each cycle during treatment with tislelizumab in combination with ociperlimab and GP regimen. After the completion of chemotherapy, they will be performed before administration on D1 of each subsequent cycle. If the laboratory tests at Screening are not performed ≤ 7 days before the first dose of study treatment, these tests should be repeated and reviewed. Additional hematology and liver function tests may be performed as necessary. The results will be reviewed within 48 hours before study treatment administration. In the event that CK-MB fractionation is not available, assess troponin I and/or troponin T instead.
12. It includes international normalized ratio, prothrombin time, and activated partial thromboplastin time.
13. Pregnancy tests will be performed at Screening, during treatment period and at EOT Visit. Urine pregnancy test (for women of childbearing potential, including women who have had a tubal ligation) must be performed and documented as negative within 7 days prior to first dose of study treatment, and repeated during treatment as clinically indicated. A serum pregnancy test must be performed if the urine pregnancy test is positive or equivocal.
14. Analysis of FT3, FT4, and TSH will be performed by the laboratories of each study site. Thyroid function tests will be performed at Screening for baseline measurement and will only be performed as clinically indicated during the study.
15. AFP, CEA, CA19-9, CA125 will be performed at Screening, every 9 weeks (± 7 days) during treatment period, and at EOT Visit.
16. Testing will be performed by the laboratories of each study site at Screening and will include HBV/HCV serology (HBsAg, HBsAb, HBcAb, and HCV antibody) and viral load assessment (HBV DNA and HCV RNA). HBV/HCV testings will be performed as clinically indicated during the study.
17. Patients who are suspected or known to have serious respiratory conditions or exhibit significant respiratory symptoms unrelated to the underlying cancer will undergo pulmonary function testing which may include, but is not limited to, spirometry and assessment of diffusion of oxygenation, at a minimum pulse oximetry at rest and with exercise, or alternatively, assessment of diffusion capacity done during the Screening period to assist the determination of suitability on the study.
18. Radiological images captured as standard of care before obtaining written informed consent and ≤ 28 days before enrollment may be used rather than repeating tests. All measurable and evaluable lesions were required to be assessed and documented at the Screening Visit and reassessed at each subsequent tumor evaluation. The same radiographic procedure used to assess disease sites at Screening is required to be used throughout the study (e.g, the same imaging protocol for CT or MRI). Imaging of the brain (preferably MRI) is required for all patients at Screening. Bone scan or PET is required if clinically indicated. During the study, tumor imaging will be performed every 9 weeks (± 7 days) per RECIST v1.1. After documentation of initial response (CR or PR), confirmation of tumor response should occur ≥ 4 weeks but ≤ 6 weeks after the initial response. Tumor assessments are required to be performed on schedule regardless of whether study treatment has been administered or held; they should not be adjusted for possible delays in cycles. Tumor assessment should continue until disease progression is determined by the investigator. Patients who discontinue study treatment early for reasons other than disease progression (e.g, toxicity) will continue to undergo tumor assessments following the original plan until the patient experiences disease progression, withdraws consent, is lost to follow-up, death or until the study terminates, whichever occurs first. See also Section 10.5.
19. Tislelizumab and ociperlimab will be administered intravenously on Day 1 of each 21-day cycle (once every 3 weeks). Tislelizumab will be administered at a fixed dose of 200 mg; Ociperlimab will be administered at a fixed dose of 900 mg; Gemcitabine 1000 mg/m^2^ + cisplatin 25 mg/m^2^ regimen will be administered on the first and eighth day of each cycle (21 days) for up to 8 cycles with chemotherapy cycles and dosage being adjusted based on tolerability. Patients who are intolerant to chemotherapy or have achieved stable disease or objective response after completing 8 cycles of chemotherapy will continue the treatment of tislelizumab 200 mg plus ociperlimab 900 mg until disease progression, unacceptable toxicity, death, withdrawal of consent, or other conditions that make the patient unsuitable for the study, as determined by the investigator.
20. Tumor tissues (formalin-fixed paraffin-embedded [FFPE] blocks or freshly cut unstained FFPE slides) will be sent for designated laboratories for retrospective analysis of exploratory biomarkers.

Appendix 2 ECOG Performance Status

| **Grade** | **Description** |
| --- | --- |
| 0 | Fully active, able to carry on all pre-diseases performance without restriction. |
| 1 | Restricted in physically strenuous activity but ambulatory and able to carry out work of a light or sedentary nature, e.g, light house work, office work. |
| 2 | Ambulatory and capable of all self-care but unable to carry out any work activities. Up and about > 50% of waking hours. |
| 3 | Capable of only limited self-care; confined to bed or chair more than 50% of waking hours. |
| 4 | Completely disabled. Cannot carry on any self-care. Totally confined to bed or chair. |
| 5 | Death. |
| As published by Oken MM, Creech RH, Tormey DC, et al. Toxicity and Response Criteria of the Eastern Cooperative Oncology Group. Am J Clin Oncol. 1982;5:649-55. | |

Appendix 3 Child-Pugh Classification Scoring System

The information presented here has been obtained from the Washington University Medical Center, with sources as follows:

- Lucey MR, Brown KA, Everson GT, Fung JJ, Gish R, Keeffe EB, et al. Minimal criteria for placement of adults on the liver transplant waiting list. Liver Transl Surg. 1997;3(6):628-637
- Pugh RNH, Murray-Lyon IN, Dawson DL, Pietroni MC, and Williams R. Transection of the esophagus for bleeding esophageal varices. Brit J Surgery. 1973;60:646-645
- Trey C, Burns DG, and Saunders SJ. Treatment of hepatic coma cornia by exchange blood transfusion. N Engl J Med. 1996;274(9):473-481

Child-Pugh classification is either Grade A (mild: score 5 to 6 points), B (moderate: from 7 to 9 points), or C (severe: from 10 to 15 points) and is determined by both clinical and biochemical parameters (as shown below).

| Clinical/biochemical parameter | Score (Severity of abnormality) | | |
| --- | --- | --- | --- |
|  | 1 | 2 | 3 |
| Hepatic encephalopathy (NCI-CTCAE grade) ^a^ | 0^b^ | 1^c^ or 2^d^ | 3^e^ or 4^f^ |
| Ascites (presence and severity) | Not at all | Mild | Moderate |
| Total bilirubin (mg/dL) | < 2.0 | 2.0 to 3.0 | > 3.0 |
| Serum albumin (g/dL) | > 3.5 | 2.8 to 3.5 | < 2.8 |
| Prothrombin time prolonged (sec)  Or  Activated partial thromboplastin time (INR^g^) | < 4 or < 1.7 | 4 to 6 or 1.7 to 2.3 | > 6 or > 2.3 |

a. Trey C, Burns DG, and Saunders SJ.Treatment of hepatic coma cornia by exchange blood transfusion.

New England Journal of Medicine 1996; 274 (9): 473-481.

b. Grade 0: Consciousness, personality, neurological examination, and electrocardiogram are all normal.

c. Grade 1: Restlessness, sleep disorders, irritability/anxiety, hand tremor, writing disorders, 5CPS waves.

d. Grade 2: Lethargy, time barrier, discomfort, asterixis, ataxia, three-phase slow wave.

e. Grade 3: Drowsiness, coma, orientation disorder, over-reflection, stiff/slow wave.

f. Grade 4: Cannot wake up from coma, no independent personality/behavior, irrational, slow 2 to 3 CPS Delta activity.

g. Lucey MR, Brown KA, Everson GT, Fung JJ, Gish R, Keeffe EB, et al.Minimal criteria for placement of adults on the liver transplant waiting list.Liver Transl Surg.1997;3(6):628-637.

Abbreviations: INR, international normalized ratio; NCI-CTCAE, National Cancer Institute Common Terminology Criteria for Adverse Events.

Appendix 4 Preexisting Immune Deficiencies or Autoimmune Diseases

Prospective patients should be carefully questioned to determine whether they have any history of an acquired or congenital immune deficiency or autoimmune disease.

Please contact the sponsor regarding any uncertain exceptions related to immune deficiency/autoimmune disease exclusions.

| Acute disseminated encephalomyelitis | Addison disease |
| --- | --- |
| Ankylosing spondylitis | Antiphospholipid antibody syndrome |
| Aplastic anemia | Autoimmune hemolytic anemia |
| Autoimmune hepatitis | Autoimmune hypoparathyroidism |
| Autoimmune hypophysitis | Autoimmune myocarditis |
| Autoimmune oophoritis | Autoimmune orchitis |
| Autoimmune thrombocytopenic purpura | Behcet disease |
| Bullous pemphigoid | Chronic inflammatory demyelinating polyneuropathy |
| Chung-Strauss syndrome | Crohn disease |
| Dermatomyositis | Dysautonomia |
| Epidermolysis bullosa | Gestational pemphigoid |
| Giant cell arteritis | Goodpasture syndrome |
| Granulomatosis with polyangiitis | Graves disease |
| Guillain-Barré syndrome | Hashimoto disease |
| Immunoglobulin A (IgA) neuropathy | Cystitis interstitial |
| Inflammatory bowel disease | Lambert-Eaton myasthenic syndrome |
| Kawasaki’s disease | Lyme disease (chronic) |
| Lupus erythematosus | Morphea |
| Mooren ulcer | Myasthenia gravis |
| Multiple sclerosis | Opsoclonus myoclonus syndrome |
| Neuromyotonia | Ord thyroiditis |
| Optic neuritis | Pernicious anemia |
| Pemphigus | Polyarthritis |
| Polyarteritis nodosa | Primary biliary cirrhosis |
| Polyglandular autoimmune syndrome | Reiter syndrome |
| Psoriasis | Sarcoidosis |
| Rheumatoid arthritis | Stiff person syndrome |
| Sjögren syndrome | Colitis ulcerative |
| Takayasu arteritis | Vogt-Kovanagi-Harada disease |

Appendix 5 Clinical Laboratory Assessments

| **Serum chemistry** | **Hematology** | **Coagulation** | **Urinalysis** |
| --- | --- | --- | --- |
| Alkaline phosphatase  Alanine Aminotransferase  Aspartate Aminotransferase  Albumin  Direct bilirubin  Total bilirubin  Blood urea nitrogen or urea  Creatinine  Glucose  Lactate dehydrogenase  Total protein  CK (creatine kinase)^1^  CK-MB (creatine kinase cardiac isoenzyme)^1^  Potassium  Sodium  Calcium  Magnesium  Chloride  Phosphorus | Red blood cell count  Hemoglobin  Haematocrit  Platelet Count  White blood cell count  Neutrophil count  Lymphocyte count | Prothrombin Time  Activated partial thromboplastin time  International Normalized Ratio | Glucose  Proteins  Ketones  Occult blood  24-hour urine protein^2^ Random urine protein to creatinine ratio^2^ |

Note: additional laboratory assessments may be performed if necessary for clinical management; data related to these assessments will be collected by the study sponsor.

1. Patients receiving study treatment will be tested for CK and CK-MB. CK and CK-MB testing is no longer required if the study treatment has been permanently discontinued. Patients with a history of heart disease or after discontinuation may undergo CK and CK-MB testing if clinically indicated.

In the event that CK-MB fractionation is not available, assess troponin I and/or troponin T instead.

1. On routine urinalysis, if urine protein is ≥ 2+ by dipstick, then obtain a 24-hour urine sample for total protein and a random urine sample for total protein and creatinine to determine a protein to creatinine ratio.

Appendix 6 Contraception Guidelines and Definitions of “Women of Childbearing Potential,” “No Childbearing Potential”

**Contraception Guidelines**

The Clinical Trials Facilitation Group’s recommendations related to contraception and pregnancy testing in clinical trials include the use of highly effective forms of birth control. These methods include the following:

- Combined (estrogen- and progestogen-containing) hormonal contraception associated with the inhibition of ovulation (oral, intravaginal, or transdermal).
- Progestogen-only hormonal contraception associated with the inhibition of ovulation (oral, injectable, or implantable).
- Intrauterine device (IUD)*.
- Intrauterine hormone-releasing system (IUS).
- Bilateral tubal occlusion.
- Vasectomized male partner.
- Sexual abstinence (defined as refraining from heterosexual intercourse during the entire period of exposure associated with the study treatment).

NOTE: Total sexual abstinence should only be used as a contraceptive method if it is in line with the patient’s usual and preferred lifestyle. Periodic abstinence (e.g, calendar, ovulation, sympto-thermal, or postovulation methods), declaration of abstinence for the duration of exposure to study treatment, and withdrawal are not acceptable methods of contraception.

Of note, barrier contraception (including male and female condoms with or without spermicide) is not considered a highly effective method of contraception and, if used, this method have to be combined with another acceptable method listed above.

**Definitions of “Women of Childbearing Potential” and “Women of No Childbearing Potential”**

As defined in this protocol, “women of childbearing potential” are female patients who are physiologically capable of becoming pregnant.

Conversely, “women of no childbearing potential” are defined as female patients meeting any of the following criteria:

• Surgically sterile (i.e, through bilateral salpingectomy, bilateral oophorectomy, or hysterectomy).

• Post-menopausal, defined as

• ≥ 55 years of age with no spontaneous menses for ≥ 12 months

• < 55 years of age with no spontaneous menses for ≥ 12 months AND with a postmenopausal follicle-stimulating hormone concentration > 30 IU/mL

Adapted from: The Clinical Trials Facilitation Group (CTFG)’s recommendations related to contraception and pregnancy testing in clinical , September 15^th^, 2014. http://www.hma.eu/fileadmin/dateien/Human_Medicines/01About_HMA/Working_Groups/CTFG/2014_09_HMA_CTFG_Contraception.pdf

Appendix 7 New York Heart Association Functional Classification

| Classification | Symptoms |
| --- | --- |
| I | No limitation of physical activity. Ordinary physical activity does not cause undue fatigue, palpitation, dyspnea (shortness of breath). |
| II | Limitation of physical activity. Comfortable at rest, but ordinary physical activity results in fatigue, palpitation, dyspnea (shortness of breath). |
| III | Marked limitation of physical activity. Comfortable at rest, but less than ordinary activity causes fatigue, palpitation, or dyspnea. |
| IV | Unable to carry on any physical activity without discomfort. Symptoms of heart failure at rest. If any physical activity is undertaken, discomfort increases. |

Appendix 8 Chronic Kidney Disease Epidemiology Collaboration (CKD-EPI) Equation

In adults, the most widely used equations for estimating glomerular filtration rate (GFR) from serum creatinine are the Chronic Kidney Disease-Epidemiology Collaboration (CKD-EPI) equation and the Modification of Diet in Renal Disease Study (MDRD) Study equation. The National Kidney Disease Education Program (NKDEP) calculators rely on creatinine determinations that are isotope dilution mass spectrometry (IDMS) traceable. All laboratories should be using creatinine methods calibrated to be IDMS traceable.

This CKD-EPI equation calculator should be used when serum creatinine (Scr) is reported in mg/dL. This equation is recommended when estimated GFR (eGFR) values above 60 mL/min/1.73 m^2^ are desired.

GFR = 141 x min (S_cr_ /κ, 1)^α^ x max(S_cr_ /κ, 1)^-1.209^ x 0.993^Age^ x 1.018 [if female] x 1.159 [if black]

Where:

S_cr_ is serum creatinine in mg/dL,

κ is 0.7 for females or 0.9 for males,

α is 0.329 for females and -0.411 for males,

min indicates the minimum of S_cr_ /κ or 1, and

max indicates the maximum of Scr /κ or 1.

The equation does not require weight because the results are reported normalized to 1.73 m^2^ body surface area, which is an accepted average adult surface area.

Online calculators for CKD-EPI can be found here:https://www.niddk.nih.gov/health-information/health-communication-programs/nkdep/lab-evaluation/gfr-calculators/Pages/gfr-calculators.aspx

Appendix 9 The Response Evaluation Criteria in Solid Tumors Guidelines (RECIST) Version 1.1

The text below has been obtained from the following reference:

- Eisenhauer EA, Therasse P, Bogaerts J, Schwartz LH, Sargent D, Ford R, et al. New Response Evaluation Criteria in Solid Tumors: Revised RECIST Guideline (Version 1.1) . Eur J Cancer.2009;45:228‑247.

**Definition**

Response and progression will be evaluated in this study using the international criteria proposed by the Response Evaluation Criteria in Solid Tumors (RECIST) Committee (Version 1.1). Changes in only the largest diameter (uni-dimensional measurement) of the tumor lesions are used in the RECIST criteria.

Note: Lesions are either measurable or non-measurable using the criteria provided below. The term “evaluable” in reference to measurability will not be used because it does not provide additional meaning or accuracy.

Measurable Disease

Tumor lesions: must be accurately measured in ≥ 1 dimension (longest diameter) with a minimum size of:

- 10 mm by CT scan (regardless of scanner type) and MRI (no less than double the slice thickness and a minimum of 10 mm)
- 10 mm caliper measurement by clinical exam (when superficial).
- 20 mm by chest x-ray (if clearly defined and surrounded by aerated lung).

Malignant lymph nodes: To be considered pathologically enlarged and measurable, a lymph node must be 15 mm in short axis when assessed by CT scan (CT scan slice thickness recommended to be no greater than 5 mm). At baseline and in follow-up, only the short axis will be measured and followed.

Non‑measurable Disease

All other lesions (or sites of disease), including small lesions (longest diameter ≥ 10 to < 15 mm with conventional techniques or < 10 mm using spiral CT scan), are considered non-measurable disease. Leptomeningeal disease, ascites, pleural, or pericardial effusion, inflammatory breast disease, lymphangitic involvement of skin or lung, abdominal masses that cannot be definitively diagnosed or monitored using imaging techniques as well as cystic lesions are all non-measurable.

*Bone lesions:*

- Bone scan, PET scan, or plain films are not considered adequate imaging techniques to measure bone lesions. However, these techniques can be used to confirm the presence or disappearance of bone lesions.
- Lytic bone lesions or mixed lytic‑blastic lesions, with identifiable soft-tissue components that can be evaluated by cross sectional imaging techniques such as CT or MRI can be considered as measurable lesions if the soft tissue component meets the definition of measurability described above.
- Blastic bone lesions are non-measurable.

*Cystic lesions:*

- Lesions that meet the criteria for radiographically defined simple cysts should not be considered as malignant lesions (neither measurable nor non‑measurable) since they are, by definition, simple cysts.
- Cystic lesions thought to represent cystic metastases can be considered measurable lesions, if they meet the definition of measurability described above. However, if non-cystic lesions are present in the same patient, these are preferred for selection as target lesions.

*Lesions with prior local treatment:*

- Tumor lesions situated in a previously irradiated area, or in an area pertaining to other loco‑regional therapy, are usually not considered measurable unless there has been demonstrated progression in the lesion. Trial protocols should detail the conditions under which such lesions would be considered measurable.

Target Lesions

All measurable lesions up to a maximum of 2 lesions per organ and 5 lesions in total should be identified as target lesions and recorded and measured at baseline. Target lesions should be selected based on their size (lesions with the longest diameter) and be representative of all involved organs, but in addition should be those that lend themselves to reproducible repeated measurements.

Lymph nodes merit special mention since they are normal anatomical structures that may be visible by imaging even if not involved by tumor. Pathological nodes that are defined as measurable and may be identified as target lesions must meet the criterion of a short axis of ≥ 15 mm by CT. Only the short axis of these nodes will contribute to the baseline sum. The short axis of the node is the diameter normally used by radiologists to judge if a node is involved by solid tumor. Nodal size is normally reported as 2 dimensions in the plane in which the image is obtained (for CT scan, this is almost always the axial plane; for MRI the plane of acquisition may be axial, sagittal, or coronal). The smaller of these measures is the short axis. For example, an abdominal node which is reported as being 20 mm x 30 mm has a short axis of 20 mm and qualifies as a malignant, measurable node. In this example, 20 mm should be recorded as the node measurement. All other pathological nodes (those with short axis ≥ 10 mm but < 15 mm) should be considered non-target lesions. Nodes that have a short axis < 10 mm are considered non-pathological and should not be recorded or followed.

A sum of the diameters (longest for non‑nodal lesions, short axis for nodal lesions) for all target lesions will be calculated and reported as the baseline sum diameters. If lymph nodes are to be included in the sum, then as noted above, only the short axis is added into the sum. The baseline sum diameters will be used as reference to further characterize any objective tumor regression in the measurable dimension of the disease.

Nontarget Lesions

All other lesions (or sites of disease), including pathological lymph nodes, should be identified as non-target lesions and should also be recorded at baseline. Measurements are not required. These lesions should be followed as “present,” “absent,” or in rare cases “unequivocal progression” (more details to follow). In addition, it is possible to record multiple non-target lesions involving the same organ as a single item on the case record form (e.g, “multiple enlarged pelvic lymph nodes” or “multiple liver metastases”).

**Guidelines for Evaluation of Measurable Disease**

All measurements should be recorded in metric notation, using calipers if clinically assessed. All baseline evaluations should be performed as close as possible to the treatment start and never more than 4 weeks before the beginning of the treatment.

The same method of assessment and the same technique should be used to characterize each identified and reported lesion at baseline and during follow-up. An imaging-based evaluation should always be done rather than a clinical examination, unless the lesion(s) being followed cannot be imaged but are assessable by clinical examination.

Clinical lesions: Clinical lesions will only be considered measurable when they are superficial and > 10 mm diameter as assessed using calipers (e.g, skin nodules). For the case of skin lesions, documentation by color photography, including a ruler to estimate the size of the lesion, is suggested. As noted above, when lesions can be evaluated by both clinical examination and imaging, imaging evaluation should be undertaken since it is more objective and may also be reviewed at the end of the study.

- Chest X-ray: Chest CT is preferred over chest X-ray, particularly when progression is an important endpoint, since CT is more sensitive than X-ray, particularly in identifying new lesions. However, lesions on chest x‑ray may be considered measurable if they are clearly defined and surrounded by aerated lung.
- CT, MRI: CT is the best currently available and reproducible method to measure lesions selected for response assessment. This guideline has defined measurability of lesions on CT scan based on the assumption that CT slice thickness is 5 mm or less. When CT scans have a slice thickness greater than 5 mm, the minimum size for a measurable lesion should be twice the slice thickness. MRI is also acceptable in certain situations (e.g, for body scans).
- Ultrasound: Ultrasound is not useful in assessment of lesion size and should not be used as a method of measurement. Ultrasound examinations cannot be reproduced in their entirety for independent review at a later date, and because they are operator dependent, it cannot be guaranteed that the same technique and measurements will be taken from one assessment to the next. If new lesions are identified by ultrasound in the course of the study, confirmation by CT or MRI is advised. If there is concern about radiation exposure at CT, MRI may be used instead of CT in selected instances.
- Endoscopy, laparoscopy: The utilization of these techniques for objective tumor response is not advised. However, they can be useful to confirm CR when biopsies are obtained or to determine relapse in trials where recurrence following CR or surgical resection is an endpoint.
- Tumor markers: Tumor markers alone cannot be used to assess objective tumor response. If markers are initially above the upper normal limit, however, they must normalize for a patient to be considered in CR. Because tumor markers are disease specific, instructions for their measurement should be incorporated into protocols on a disease-specific basis. Specific guidelines for both CA‑125 response (in recurrent ovarian cancer) and prostate‑specific antigen (PSA) response (in recurrent prostate cancer), have been published. In addition, the Gynecologic Cancer Intergroup has developed CA-125 progression criteria that are to be integrated with objective tumor assessment for use in first-line trials in ovarian cancer.
- Cytology, histology: These techniques can be used to differentiate between PR and CR in rare cases if required by protocol (for example, residual lesions in tumor types such as germ cell tumors, where known residual benign tumors can remain). When effusions are known to be a potential adverse effect of treatment (e.g, with certain taxane compounds or angiogenesis inhibitors), the cytological confirmation of the neoplastic origin of any effusion that appears or worsens during treatment can be considered if the measurable tumor has met criteria for response or stable disease in order to differentiate between response (or stable disease) and progressive disease.

**Response Criteria**

Evaluation of Target Lesions

- Complete response (CR): Disappearance of all target lesions. Any pathological lymph nodes (whether target or non-target) must have reduction in short axis to < 10 mm.
- Partial Response (PR): At least a 30% decrease in the sum of the diameters of target lesions, taking as reference the baseline sum diameters.
- Progressive Disease (PD): At least a 20% increase in the sum of diameters of target lesions, taking as reference the smallest sum on study (this includes the baseline sum if that is the smallest on study). In addition to the relative increase of 20%, the sum must also demonstrate an absolute increase of at least 5 mm. (Note: The appearance of 1 or more new lesions is also considered progression).
- Stable Disease (SD): Neither sufficient shrinkage to qualify for PR nor sufficient increase to qualify for PD, taking as reference the smallest sum diameters while on study.
- Lymph nodes: Lymph nodes identified as target lesions should always have the actual short axis measurement recorded (measured in the same anatomical plane as the baseline examination), even if the nodes regress to < 10 mm on study. This means that when lymph nodes are included as target lesions, the “sum” of lesions may not be zero even if complete response criteria are met, since a normal lymph node is defined as having a short axis of < 10 mm. Case report recorded in a separate section where, to qualify for CR, each node must achieve a short axis < 10 mm. For PR, SD and PD, the actual short axis measurement of the nodes is to be included in the sum of target lesions.
- Target lesions that become “too small to measure”: While on study, all lesions (nodal and non- nodal) recorded at baseline should have their actual measurements recorded at each subsequent evaluation, even when very small (e.g, 2 mm). However, sometimes lesions or lymph nodes which are recorded as target lesions at baseline become so faint on a CT scan that the radiologist may not feel comfortable assigning an exact measure and may report them as being “too small to measure.”

When this occurs it is important that a value be recorded on the eCRF. If it is the opinion of the radiologist that the lesion has likely disappeared, the measurement will be recorded as 0 mm. If the lesion is believed to be present and is faintly seen but too small to measure, a default value of 5 mm should be assigned (Note: It is less likely that this rule will be used for lymph nodes since they usually have a definable size when normal and are frequently surrounded by fat, such as in the retroperitoneum; however, if a lymph node is believed to be present and is faintly seen but too small to measure, a default value of 5 mm should be assigned in this circumstance as well). This default value is derived from the 5 mm CT slice thickness (but should not be changed with varying CT slice thickness). The measurement of these lesions is potentially non-reproducible; therefore, providing this default value will prevent false responses or progressions based upon measurement error. To reiterate, however, if the radiologist is able to provide an actual measure, that should be recorded, even if it is below 5 mm.

- Lesions that split or coalesce on treatment: When non‑nodal lesions “fragment,” the longest diameters of the fragmented portions should be added together to calculate the target lesion sum. Similarly, as lesions coalesce, a plane between them may be maintained that would aid in obtaining maximal diameter measurements of each individual lesion. If the lesions have truly coalesced such that they are no longer separable, the vector of the longest diameter in this instance should be the maximal longest diameter for the “coalesced lesion.”

Evaluation of Nontarget Lesions

While some non-target lesions may be measurable, they need not be measured and instead should be assessed only qualitatively at the timepoints specified in the protocol.

- CR: Disappearance of all non‑target lesions and normalization of tumor marker level. All lymph nodes must be non-pathological in size (< 10 mm short axis).
- PD: Unequivocal progression (as detailed below) of existing non-target lesions. (Note: the appearance of 1 or more new lesions is also considered progression.)
- Non‑CR/Non‑PD: Persistence of 1 or more non-target lesion(s) and/or maintenance of tumor marker(s) level above the normal limits.
- When the patient also has measurable disease: In this setting, to achieve “unequivocal progression” on the basis of the non-target disease, there must be an overall level of substantial worsening in non-target disease such that, even in presence of SD or PR in target disease, the overall tumor burden has increased sufficiently to merit discontinuation of therapy. A modest “increase” in the size of 1 or more non-target lesions is usually not sufficient to qualify for unequivocal progression status. The designation of overall progression solely on the basis of change in non-target disease in the face of SD or PR of target disease will therefore be extremely rare.
- When the patient has only non-measurable disease: This circumstance arises in some phase 3 trials when it is not a criterion of trial entry to have measurable disease. The same general concept applies here as noted above; however, in this instance there is no measurable disease assessment to factor into the interpretation of an increase in non‑measurable disease burden. Because worsening in non-target disease cannot be easily quantified (by definition: if all lesions are truly non-measurable), a useful test that can be applied when assessing patients for unequivocal progression is to consider if the increase in overall disease burden based on the change in non-measurable disease is comparable in magnitude to the increase that would be required to declare PD for measurable disease; i.e, an increase in tumor burden representing an additional 73% increase in “volume” (which is equivalent to a 20% increase in diameter in a measurable lesion).

Examples include an increase in a pleural effusion from “trace” to “large,” an increase in lymphangitic disease from localized to widespread, or it may be described in the protocol as “sufficient to require a change in therapy.” If “unequivocal progression” is seen, the patient should be considered to have had overall progressive disease at that point. While it would be ideal to have objective criteria to apply to non-measurable disease, the very nature of that disease makes it impossible to do so; therefore, the increase must be substantial.

New Lesion(s) Present

The appearance of new malignant lesions denotes disease progression; therefore, some comments on detection of new lesions are important. There are no specific criteria for the identification of new radiographic lesions; however, the finding of a new lesion should be unequivocal: i.e, not attributable to differences in scanning technique, change in imaging modality or findings thought to represent something other than tumor (for example, some “new” bone lesions may be simply healing or flare of preexisting lesions). This is particularly important when the patient’s baseline lesions show partial or complete response. For example, necrosis of a liver lesion may be reported on a CT scan report as a “new” cystic lesion, which it is not.

A lesion identified on a follow‑up study in an anatomical location that was not scanned at baseline is considered a new lesion and will indicate disease progression. An example of this is the patient who has visceral disease at baseline and while on trial has a CT or MRI brain ordered which reveals metastases. The patient’s brain metastases are considered evidence of PD even if he or she did not have brain imaging at baseline.

If a new lesion is equivocal, for example because of its small size, continued therapy and follow‑up evaluation will clarify if it represents a truly new disease. If repeat scans confirm that there is definitely a new lesion, then progression should be declared using the date of the initial scan.

While fluorodeoxyglucose (FDG)-PET response assessments need additional study, it is sometimes reasonable to incorporate the use of FDG-PET scanning to complement CT scanning in assessment of progression (particularly possible “new” disease). New lesions on the basis of FDG‑PET imaging can be identified according to the following algorithm:

- Negative FDG-PET at baseline, with a positive FDG-PET at follow-up, is a sign of progressive disease based on a new lesion.
- No FDG-PET at baseline and a positive FDG-PET at follow-up: If the positive FDG‑PET at follow‑up corresponds to a new site of disease confirmed by CT, this is progressive disease. If the positive FDG-PET at follow-up is not confirmed as a new site of disease on CT, additional follow-up CT scans are needed to determine if there is truly progression occurring at that site (if so, the date of PD will be the date of the initial abnormal FDG‑PET scan). If the positive FDG-PET at follow-up corresponds to a pre-existing site of disease on CT that is not progressing on the basis of the anatomic images, this is not PD.

Evaluation of Best Overall Response

The best overall response is the best response recorded from the start of the study treatment treatment until the EOT considering any requirement for confirmation. On occasion, a response may not be documented until after the end of therapy so protocols should be clear if post‑treatment assessments are to be considered in determination of BOR. Protocols must specify how any new therapy introduced before progression will affect best response designation. The patient’s best overall response assignment will depend on the findings of both target and non-target disease and will also take into consideration the appearance of new lesions. Furthermore, depending on the nature of the trial and the protocol requirements, it may also require confirmatory measurement. Specifically, in non-randomized trials where response is the primary endpoint, confirmation of PR or CR is needed to deem either one the “best overall response”.

The BOR is determined once all the data for the patient is known. Best response determination in trials where confirmation of complete or partial response **IS NOT** required: Best response in these trials is defined as the best response across all time points (for example, a patient who has SD at first assessment, PR at second assessment, and PD on last assessment has a BOR of PR). When stable disease is believed to be best response, it must also meet the protocol-specified minimum time from baseline. If the minimum time is not met when SD is otherwise the best time point response, the patient’s best response depends on the subsequent assessments. For example, a patient who has achieved stable disease at first assessment, PD at second and does not meet minimum duration for stable disease, will have a best response of PD. The same patient lost to follow‑up after the first SD assessment would be considered unevaluable.

| **Target Lesions** | **Nontarget Lesions** | **New lesion(s) present** | **Overall Response** |
| --- | --- | --- | --- |
| CR | CR | No | CR |
| CR | Non-CR/non‑PD | No | PR |
| CR | Not evaluated | No | PR |
| PR | Non‑PD or not all evaluated | No | PR |
| SD | Non‑PD or not all evaluated | No | SD |
| Not all evaluated | Non-PD | No | NE |
| PD | Any | Yes or No | PD |
| Any | PD | Yes or No | PD |
| Any | Any | Yes | PD |

Abbreviations: CR, complete response; NE, not evaluable; PD , progressive disease; PR, partial response; SD, stable disease.

When nodal disease is included in the sum of target lesions and the nodes decrease to “normal” size (< 10 mm), they may still have a measurement reported on scans. This measurement should be recorded even though the nodes are normal in order to not to overstate progression should it be based on increase in the size of the nodes. As noted earlier, this means that patients with CR may not have a total sum of “zero” on the eCRF.

In trials where confirmation of response is required, repeated “NE” time point assessments may complicate best response determination. The analysis plan for the study must address how missing data/assessments will be addressed in determination of the response and progression. For example, in most trials it is reasonable to consider a patient with time point responses of PR‑NE‑PR as a confirmed response.

Patients with a global deterioration of health status requiring discontinuation of treatment without objective evidence of disease progression at that time should be reported as “symptomatic deterioration.” Every effort should be made to document objective progression even after discontinuation of treatment. Symptomatic deterioration is not a descriptor of an objective response: it is a reason for stopping trial therapy.

Conditions that define “early progression, early death, and unevaluability” are trial specific and should be clearly described in each protocol (depending on treatment duration, treatment periodicity).

In some circumstances it may be difficult to distinguish residual disease from normal tissue. When the evaluation of complete response depends upon this determination, it is recommended that the residual lesion be investigated (fine‑needle aspirate/biopsy) before assigning a status of complete response. FDG‑PET may be used to upgrade a response to CR in a manner similar to a biopsy in cases where a residual radiographic abnormality is thought to represent fibrosis or scarring. The use of FDG‑PET in this circumstance should be prospectively described in the protocol and supported by disease specific medical literature for the indication. However, it must be acknowledged that both approaches may lead to false positive CR due to limitations of FDG‑PET and biopsy resolution/sensitivity.

For equivocal findings of progression (e.g, very small and uncertain new lesions. cystic changes, or necrosis in existing lesions), treatment may continue until the next scheduled assessment. If at the next scheduled assessment, progression is confirmed, the date of progression should be the earlier date when progression was suspected.

**Confirmatory Measurement/Duration of Response**

Confirmation

In non-randomized studies where response is the primary endpoint, confirmation of PR and CR is required to ensure responses identified are not the result of measurement error. This will also permit appropriate interpretation of results in the context of historical data where response has traditionally required confirmation in such trials. However, in all other circumstances, i.e, in randomized trials (phase 2 or 3) or trials where stable disease or PD are the primary endpoints, confirmation of response is not required since it will not add value to the interpretation of trial results. However, elimination of the requirement for response confirmation may increase the importance of central review to protect against bias, in particular in trials that are not blinded.

In the case of stable disease, measurements must have met the stable disease criteria at least once after trial entry at a minimum interval (in general not less than 6 weeks).

Duration of Overall Response

The duration of overall response is measured from the time measurement criteria are first met for CR/PR (whichever is first recorded) until the first date that recurrent or PD is objectively documented (taking as reference for PD the smallest measurements recorded on study).

The duration of overall complete response is measured from the time measurement criteria are first met for CR until the first date that recurrent disease is objectively documented.

Duration of Stable Disease

Stable disease is measured from the start of the treatment (in randomized trials, from date of randomization) until the criteria for progression are met, taking as reference the smallest sum on study (if the baseline sum is the smallest, this is the reference for calculation of progressive disease).

The clinical relevance of the duration of SD varies in different studies and diseases. If the proportion of patients achieving stable disease for a minimum period is an endpoint of importance in a particular trial, the protocol should specify the minimal time interval required between 2 measurements for determination of stable disease.

Note: The duration of response and SD as well as the progression-free survival are influenced by the frequency of follow-up after baseline evaluation. It is not in the scope of this guideline to define a standard follow‑up frequency. The frequency should take into account many parameters including disease types and stages, treatment periodicity, and standard practice. However, these limitations of the precision of the measured endpoints should be taken into account if comparisons.

Appendix 10 Immune‑Related Adverse Event Evaluation and Management

The recommendations below for the diagnosis and management of any irAE are intended as a guidance. This document should be used in conjunction with expert clinical judgment (by specialist physicians experienced in the treatment of cancer using immunological agents) and individual institutional guidelines or policies.

Criteria used to diagnose irAEs include blood tests, diagnostic imaging, histopathology, and microbiology assessments to exclude alternative causes such as infection, disease progression, and adverse effects of concomitant drugs. In addition to the results of these tests, the following factors should be considered when making an irAE diagnosis:

- What is the temporal relationship between initiation of tislelizumab and/or ociplerlimab and the adverse event?
- How did the patient respond to withdrawal of tislelizumab and/or ociperlimab?
- Did the event recur when tislelizumab and/or ociperlimab was (were) reintroduced?
- Was there a clinical response to corticosteroids?
- Is the event an autoimmune endocrinopathy?
- Is disease progression or an alternative diagnosis a more likely explanation?

When alternative explanations to autoimmune toxicity have been excluded, the irAE field associated with the AE on the eCRF should be checked.

| **Recommended Diagnostic Tests in the Management of Possible Immune-Related Adverse Events** | |
| --- | --- |
| **Immune-Related Toxicity** | **Diagnostic Evaluation Guideline** |
| Thyroid disorder(s) | Scheduled and repeated thyroid function tests (TSH and T4). |
| Hypophysitis | Check visual fields and consider pituitary endocrine axis blood profile. Perform pituitary and whole brain MRI in patients with headache, visual disturbance, unexplained fatigue, asthenia, weight loss, and unexplained constitutional symptoms.  Consider consultation with an endocrinologist if an abnormality is detected. |
| Pneumonitis | All patients presenting with new or worsened pulmonary symptoms or signs, such as an upper respiratory infection, new cough, shortness of breath, or hypoxia should be assessed by high‑resolution CT. Consider pulmonary function test including DLCO.  Radiographic appearance is often nonspecific. Depending on the location of the abnormality, bronchoscopy and bronchoalveolar lavage or lung biopsy may be considered. Consult with a respiratory medicine physician for cases of uncertain cause. |
| Neurotoxicity | Perform a comprehensive neurological examination and brain MRI for all CNS symptoms; review alcohol history and other medications. Conduct a diabetic screen, and assess blood B12/folate, HIV status, TFTs, and consider autoimmune serology. Consider the need for brain/spine MRI/MRA and nerve conduction study for peripheral neuropathy. Consult with a neurologist if there are abnormal findings. |
| Colitis | Review dietary intake and exclude steatorrhea. Consider comprehensive testing, including the following: FBC, UEC, LFTs, CRP, TFTs, stool microscopy and culture, viral PCR, *Clostridium Difficile* toxin, as well as cryptosporidia (drug-resistant organism).  In case of abdominal discomfort, consider imaging, e.g, X-ray, CT scan. If a patient experiences bleeding, pain, or distension, consider colonoscopy with biopsy and surgical intervention as appropriate. |
| Eye disorders | If a patient experiences acute, new onset, or worsening of eye inflammation; blurred vision; or other visual disturbances, refer the patient urgently to an ophthalmologist for evaluation and management. |
| Hepatitis | Check ALT/AST/total bilirubin, INR/albumin; the frequency will depend on severity of the AE (e.g, daily if Grade 3 to 4; every 2 to 3 days if Grade 2, until recovering). Review medications (e.g, statins, antibiotics) and alcohol history. Perform liver screen including hepatitis A/B serology, hepatitis E PCR and assess anti-ANA/SMA/LKM/SLA/LP/LCI, iron studies. Consider imaging, e.g, ultrasound scan, for metastases or thromboembolism. Consult with a hepatologist and consider liver biopsy. |
| Renal toxicity | Review hydration status and medication history. Test and culture urine. Consider renal ultrasound scan, protein assessment (dipstick/24-hour urine collection), or phase‑contrast microscopy. Refer to a nephrologist for further management assistance. |
| Dermatosis | Consider other causes by conducting a physical examination, consider dermatology referral for skin biopsy. |
| Joint or muscle inflammation | Conduct musculoskeletal history and perform complete musculoskeletal examination. Consider joint x-ray and other imaging as required to exclude metastatic disease. Perform autoimmune serology and refer to rheumatology for further management assistance.  For suspected myositis/rhabdomyolysis/muscular weakness include: CK, ESR, CRP, troponin and consider a muscle biopsy. |
| Myocarditis | Perform ECG, echocardiogram, CK/CK-MB, and troponin (I and/or T) and refer to a cardiologist. |

Abbreviations: AE, adverse event; ALT, alanine aminotransferase; ANA, antinuclear antibody; AST, aspartate aminotransferase; CK, creatinine kinase; CK-MB, creatinine kinase cardiac isoenzyme; CNS, central nervous system; CRP, C-reactive protein; CT, computed tomography; DLCO, diffusing capacity for carbon monoxide; ECG, electrocardiogram; ESR, erythrocyte sedimentation rate; FBC, complete blood count; HIV, human immunodeficiency virus; INR, international normalized ratio; LCI, liver cytosolic antigen; LFT, liver function test; LKM, liver kidney microsomal antibody; LP, liver pancreas antigen; MRA, magnetic resonance angiogram; MRI, magnetic resonance imaging; PCR, polymerase chain reaction; SLA, soluble liver antigen; SMA, smooth muscle antibody; T4, thyroxine; TFT, thyroid function tests; TSH, thyroid‑stimulating hormone; UEC, urea electrolytes and creatinine.

**Treatment of Immune-Related Adverse Events**

- The irAEs can escalate quickly; study treatment interruption, close monitoring, timely diagnostic work-up, and treatment intervention, as appropriate, with patient is required.
- The irAEs should improve promptly after introduction of immunosuppressive therapy. If this does not occur, review the diagnosis, seek further specialist advice, and contact the medical monitor
- For some Grade 3 toxicities that resolve quickly, rechallenge with study treatment may be considered if there is evidence of a clinical response to study treatment, after consultation with the study medical monitor.
- Steroid dosages in the table below are for oral or intravenous (methyl)prednisolone. Equivalent dosages of other corticosteroids can be substituted. For corticosteroid-refractory irAEs, consider use of steroid-sparing agents (e.g, mycophenolate mofetil [MMF]).
- Consider prophylactic antibiotics for opportunistic infections if the patient is receiving long-term immunosuppressive therapy
- Please also refer to latest ASCO practice guidelines on the management of irAEs in patients treated with immune checkpoint inhibitor therapy.

| **Autoimmune Toxicity** | **Grade** | **Treatment Guidelines (Subject to Clinical Judgment)** | **Study Treatment Management** |
| --- | --- | --- | --- |
| **Thyroid disorder(s)** | **1-2**  Asymptomatic TFT abnormality or mild symptoms | Replace thyroxine if hypothyroid, until TSH/T4 levels return to normal range.  Thyrotoxic patients should be referred to an endocrinologist. In cases with systemic symptoms: withhold study treatment, treat with a beta blocker and consider oral prednisolone 0.5 mg/kg/d for thyroid pain. Taper corticosteroids over 2 to 4 weeks. Monitor thyroid function regarding the need for hormone replacement. | Continue study treatment or withhold treatment in cases with systemic symptoms. |
|  | **3-4**  Severe symptoms, hospitalization required | Refer the patient to an endocrinologist.  If hypothyroid, replace with thyroxine 0.5 to 1.5 µg/kg/d (for the elderly or those with comorbidities, the suggested starting dose is 0.5 µg/kg/d). Add oral prednisolone 0.5 mg/kg/d for thyroid pain. Thyrotoxic patients require treatment with a beta blocker and may require carbimazole until thyroiditis resolves. | Hold study treatment; resume when resolved/improved to Grade 0 to 1. |
| **Hypophysitis** | **1-2**  Mild-moderate symptoms | Refer the patient to an endocrinologist for hormone replacement. Add oral prednisolone 0.5 to 1 mg/kg/d for patients with pituitary inflammation. Taper corticosteroids over at least 1 month. If there is no improvement in 48 hours, treat as a Grade 3 to 4 event. Taper corticosteroids over at least 1 month. | Continue study treatment. |
|  | **3-4**  Severe or life-threatening symptoms | Refer the patient to an endocrinologist for assessment and treatment. Initiate pulse intravenous methylprednisolone 1 mg/kg for patients with headache/visual disturbance due to pituitary inflammation. Convert to oral prednisolone and taper over at least 1 month. Maintain hormone replacement according to endocrinologist’s advice. Maintain hormone replacement according to endocrinologist’s advice. | Hold study treatment for patients with headache/visual disturbance due to pituitary inflammation until resolved/improved to ≤ Grade 2. Discontinuation is usually not necessary. |
| **Pneumonitis** | **1**  Radiographic changes only | Monitor symptoms every 2 to 3 days.  If appearance worsens, treat as a Grade 2 event. | Consider holding study treatment until appearance improves and cause is determined. |
|  | **2**  Symptomatic: dyspnoea exertional | Initiate antibiotics if infection suspected. Add oral prednisolone 1 mg/kg/d if symptoms/appearance persist for 48 hours or worsen. Consider prophylaxis for pneumocystis infection. Taper corticosteroids over at least 6 weeks.  Consider prophylaxis for adverse steroid effects, e.g, blood glucose monitoring, vitamin D/calcium supplement. | Hold study treatment. Re-treatment is acceptable if symptoms resolve completely or are controlled on prednisolone ≤ 10 mg/d. Discontinue study treatment if symptoms persist with corticosteroid treatment. |
|  | **3-4**  Severe or life-threatening symptoms  Unable to breathe at rest | Admit to a hospital and initiate IV methylprednisolone 2 to 4mg/kg/d. If there is no improvement, or worsening after 48 hours, add infliximab 5 mg/kg (if no hepatic involvement). Convert to oral prednisolone and taper over at least 2 month. Cover with empiric antibiotics and consider prophylaxis for pneumocystis infection and other adverse steroid effects, e.g, blood glucose monitoring, vitamin D/calcium supplement. | Discontinue study treatment. |
| **Neurotoxicity** | **1**  Mild symptoms |  | Continue study treatment. |
|  | **2**  Moderate symptoms | Treat with oral prednisolone 0.5 to 1 mg/kg/d. Taper over at least 4 weeks. Consult with a neurologist. | Hold study treatment; resume when resolved/improved to Grade 0 to 1. |
|  | **3-4**  Severe/life-threatening | Initiate treatment with oral prednisolone or IV methylprednisolone 1 to 2 mg/kg/d, depending on symptoms. Taper corticosteroids over at least 4 weeks.  Consider azathioprine, MMF, cyclosporine if no response within 72 to 96 hours. | Discontinue study treatment. |
| **Colitis/Diarrhea** | **1**  Mild symptoms. < 3 liquid stools per day over baseline and feeling well | Symptomatic management: administer hydration, loperamide, avoid high fiber/lactose diet.  If grade 1 persists for > 14 days manage as a Grade 2 event. | Continue study treatment. |
|  | **2**  Moderate symptoms 4 to 6 liquid stools per day over the baseline, or abdominal pain, or blood in stool, or nausea, or nocturnal episodes | Oral prednisolone 0.5 mg/kg/d (non-enteric coated).  Do not wait for any diagnostic tests to start treatment. Taper steroids over 2 to 4 weeks, consider endoscopy if symptoms are recurring. | Hold study treatment; resume when resolved/improved to the baseline grade. |
|  | **3**  Severe symptoms: > 6 liquid stools per day over the baseline, or if episodic within 1 hour of eating | Initiate IV methylprednisolone 1 to 2 mg/kg/d.  Convert to oral prednisolone and taper over at least 4 weeks. Consider prophylaxis for adverse steroid effects, e.g, blood glucose monitoring, vitamin D/calcium supplement.  If no improvement in 72 hours or symptoms worsen, consider infliximab 5 mg/kg if no perforation, sepsis, TB, hepatitis, NYHA Class III/IV CHF or other immunosuppressive treatment: MMF or tacrolimus.  Consult a gastroenterologist to conduct colonoscopy/sigmoidoscopy. | Hold study treatment; retreatment may be considered when resolved/improved to the baseline grade and after discussion with the study medical monitor. |
|  | **4**  Life-threatening symptoms |  | Discontinue study treatment. |
| **Cutaneous Reactions** | **1**  Skin rash, with or without symptoms, < 10% BSA | Avoid skin irritants and sun exposure; topical emollients recommended. | Continue study treatment. |
|  | **2**  Rash covers 10% to 30% of BSA | Avoid skin irritants and sun exposure; topical emollients recommended.  Topical steroids (moderate strength cream once a day or potent cream twice a day) ± oral or topical antihistamines for itch. Consider a short course of oral steroids. | Continue study treatment. |
|  | **3**  Rash covers > 30% BSA or Grade 2 with substantial symptoms | Avoid skin irritants and sun exposure; topical emollients recommended.  Initiate steroids as follows based on clinical judgment:  For moderate symptoms: oral prednisolone 0.5 to 1 mg/kg/d for 3 days then taper over 2 to 4weeks  For severe symptoms: IV methylprednisolone 0.5 to 1 mg/kg/d; convert to oral prednisolone and taper over at least 4 weeks. | Hold study treatment.  Re-treat when AE is resolved or improved to mild rash (Grade 1 to 2) after discussion with the study medical monitor. |
|  | **4**  Skin sloughing > 30% BSA with associated symptoms (e.g, erythema, purpura, epidermal detachment) | Initiate IV methylprednisolone 1 to 2 mg/kg/d. Convert to oral prednisolone and taper over at least 4 weeks.  Admit to a hospital and seek urgent dermatology consultation. | Discontinue study treatment. |
| **Hepatitis** | **1**  ALT or AST > ULN to 3 x ULN | Check liver function tests (LFTs) within 1 week and before the next dose to verify that there has been no worsening.  If liver function tests are worsening, recheck every 48 to 72 hours until improvement is seen. | Continue study treatment if LFTs are unchanged or improving.  Hold study treatment if LFTs are worsening until improvement is seen. |
|  | **2**  ALT or AST > 3 x to 5 x ULN | Recheck liver function tests within 48 to 72 hours:  For persistent ALT/AST elevation: consider oral prednisolone 0.5 to 1 mg/kg/d for 3 days, then taper over 2 to 4 weeks.  For rising ALT/AST: start oral prednisolone 1 mg/kg/d and taper over 2 to 4 weeks; re-escalate dose if LFTs worsen, depending on clinical judgment. | Hold study treatment, treatment may be resumed when resolved/improved to the baseline grade and prednisolone tapered to ≤ 10 mg. |
|  | **3**  ALT or AST > 5 x to 20 x ULN | ALT/AST < 400 IU/L and normal bilirubin/INR/albumin: Initiate oral prednisolone 1 mg/kg and taper over at least 4 weeks.  ALT/AST > 400 IU/L or rising bilirubin/INR/low albumin: Initiate intravenous (methyl)prednisolone 2 mg/kg/d. When LFTs improve to ≤ Grade 2, convert to oral prednisolone and taper over at least 4 weeks. | Hold study treatment until improved to the baseline grade; reintroduce only after discussion with the study medical monitor. |
|  | **4**  ALT or AST > 20 x ULN | Initiate IV methylprednisolone 1 to 2 mg/kg/d. Convert to oral prednisolone and taper over at least 6 weeks. | Discontinue study treatment. |
|  | **Worsening Liver Function Tests Despite Steroids:**   - If on oral prednisolone, change to pulsed intravenous methylprednisolone. - If on IV, add MMF 500-1000 mg twice a day. - If worsens on MMF, consider addition of tacrolimus.   Duration and dose of steroid(s) required will depend on severity of event. | | |
| **Nephritis** | **1**  Creatinine 1.5 x baseline  Or > ULN to 1.5 x ULN | Repeat creatinine weekly.  If symptoms worsen, manage as per criteria below. | Continue study treatment. |
|  | **2**  Creatinine > 1.5 to 3 x baseline or > 1.5 to 3 x ULN | Ensure hydration and review creatinine in 48 to 72 hours; if not improving, consider creatinine clearance measurement by 24-hour urine collection. Discuss with nephrologist the need for kidney biopsy.  If attributed to study treatment, initiate oral prednisolone 0.5 to 1 mg/kg and taper over at least 2 weeks.  Repeat creatinine/U&E every 48 to 72 hours. | Hold study treatment.  If not attributed to drug toxicity, restart study treatment.  If attributed to study treatment and resolved/improved to the baseline grade: Restart study treatment if tapered to < 10 mg prednisolone. |
|  | **3**  Creatinine > 3 x baseline or > 3 to 6 x ULN | Hospitalize patient for monitoring and fluid balance; repeat creatinine every 24 hours; refer to a nephrologist and discuss need for biopsy. If worsening, initiate IV (methyl)prednisolone 1 to 2 mg/kg/d. Taper corticosteroids over at least 4 weeks. | Hold study treatment until the cause is investigated.  If study treatment suspected: discontinue study treatment. |
|  | **4**  Creatinine > 6 x ULN | As per Grade 3, patient should be managed in a hospital where renal replacement therapy is available. | Discontinue study treatment. |
| **Diabetes/ Hyperglycemia** | **1**  Fasting glucose value ULN to 160 mg/dL; ULN to 8.9 mmol/L | Monitor closely and treat according to local guideline. Check for C‑peptide and antibodies against glutamic acid decarboxylase and islet cells are recommended. | Continue study treatment. |
|  | **2**  Fasting glucose value 160-250 mg/dL; 8.9- 13.9 mmol/L | Obtain a repeat blood glucose level at least every week. Management as per local guideline. | Continue study treatment or hold treatment if hyperglycemia is worsening. Resume treatment when blood glucose is stabilized at baseline or Grade 0 to 1. |
|  | **3**  Fasting glucose value 250-500 mg/dL; 13.9-27.8 mmol/L | Admit patient to hospital and refer to a diabetologist for hyperglycemia management. Corticosteroids may exacerbate hyperglycemia and should be avoided. | Hold study treatment until patient is hyperglycemia symptom-free, and blood glucose has been stabilized at baseline or Grade 0 to 1. |
|  | **4**  Fasting glucose value > 500 mg/dL; > 27.8 mmol/L | Admit patient to hospital and institute local emergency diabetes management. Refer the patient to a diabetologist for insulin maintenance and monitoring. |  |
| Ocular toxicity | **1**  Asymptomatic eye examination/test abnormality | Consider alternative causes and prescribe topical treatment as required. | Continue study treatment. |
|  | **2**  Anterior uveitis or mild symptoms | Refer patient to an ophthalmologist for assessment and topical corticosteroid treatment. Consider a course of oral steroids. | Continue study treatment or hold treatment if symptoms worsen or if there are symptoms of visual disturbance. |
|  | **3**  Posterior uveitis/panuveitis or significant symptoms | Refer patient urgently to an ophthalmologist. Initiate oral prednisolone 1 to 2 mg/kg and taper over at least 4 weeks. | Hold study treatment unless improved to Grade 0 to 1; reintroduce only after discussion with the study medical monitor. |
|  | **4**  Blindness (at least 20/200 or worse) in the affected eyes | Initiate IV (methyl)prednisolone 2 mg/kg/d. Convert to oral prednisolone and taper over at least 4 weeks. | Discontinue study treatment. |
| Pancreatitis | **2**  Asymptomatic, blood test abnormalities | Monitor pancreatic enzymes | Continue study treatment. |
|  | **3**  Abdominal pain, nausea and vomiting | Admit to hospital for urgent management. Initiate IV (methyl)prednisolone 1 to 2 mg/kg/d. Convert to oral prednisolone when amylase/lipase improved to Grade 2, and taper over at least 4 weeks | Hold study treatment; reintroduce only after discussion with the study medical monitor. |
|  | **4**  Acute abdominal pain, surgical emergency | Admit to hospital for emergency management and considering local consultation. | Discontinue study treatment. |
| Arthritis | **1**  Mild pain with inflammation, swelling | Management as per local guideline | Continue study treatment. |
|  | **2**  Moderate pain with inflammation, swelling, limited instrumental (fine motor) activities | Management as per local guideline. Consider referring to rheumatology. If symptoms worsen on treatment, manage as a Grade 3 event. | Continue treatment or, if symptoms continue to worsen, hold study treatment until symptoms improve to baseline or Grade 0 to 1. |
|  | **3**  Severe pain with inflammation or permanent joint damage, daily living activity limited | Refer patient urgently to a rheumatologist for assessment and management. Initiate oral prednisolone 0.5 to 1 mg/kg and taper over at least 4 weeks. | Hold study treatment unless improved to Grade 0 to 1; reintroduce only after discussion with the study medical monitor. |
| Mucositis/Stomatitis | **1**  Test findings only or minimal symptoms | Consider topical treatment or analgesia as per local guideline. | Continue study treatment. |
|  | **2**  Moderate pain, reduced oral intake, limited instrumental activities | As per local guidelines, treat with analgesics, topical treatments, and oral hygiene care. Ensure adequate hydration. If symptoms worsen or there is sepsis or bleeding, manage as a Grade 3 event. | Continue study treatment. |
|  | **3**  Severe pain, limited food and fluid intake, daily living activity limited | Admit to hospital for appropriate management. Initiate IV (methyl)prednisolone 1 to 2 mg/kg/d. Convert to oral prednisolone when symptoms improved to Grade 2 and taper over at least 4 weeks. | Hold study treatment until improved to Grade 0 to 1. |
|  | **4**  Life-threatening complications or dehydration | Admit to hospital for urgent management. If not contraindicated, IV corticosteroids are given to manage infection. | Discontinue study treatment. |
| **Myositis/ Rhabdomyolysis** | **1**  Mild weakness with/without pain | Treat with analgesics.  If CK is significantly elevated and patient has symptoms, consider oral steroids and treat as a Grade 2 event. | Continue study treatment. |
|  | **2**  Moderate weakness with/without pain | If CK is 3 x ULN or worse, initiate oral prednisolone 0.5 to 1 mg/kg and taper over at least 4 weeks. | Hold study treatment until improved to Grade 0 to 1. |
|  | **3-4**  Severe weakness, limiting self-care | Admit to hospital and initiate oral prednisolone 1 mg/kg. Consider bolus IV (methyl)prednisolone and1 to 2 mg/kg/d maintenance for severe activity restriction or dysphagia. If symptoms do not improve, add immunosuppressant therapy. Taper oral corticosteroids over at least 4 weeks. | Hold study treatment until improved to Grade 0 to 1. Discontinue upon any evidence of myocardial weakness. |
| **Myocarditis** | **< 2**  Asymptomatic but significantly elevated CK-MB/troponin or clinically significant intraventricular conduction delay. | Initiate close monitoring of serum myocardial markers to assess cardiac status; consider referring the patient to a cardiologist.  If diagnosis of myocarditis is confirmed, treat as a Grade 2 event. | Hold study treatment.  If a diagnosis of myocarditis is confirmed, permanently discontinue study treatment in patients with moderate or severe symptoms. Tislelizumab may not be restarted in asymptomatic or less symptomatic patients unless cardiac findings recover to baseline, after full discussion with the study monitor. |
|  | **2**  Symptoms on mild-moderate exertion | Admit to hospital and initiate oral prednisolone or intravenous methylprednisolone at 1 to 2 mg/kg/d. Consult with a cardiologist and manage symptoms of cardiac failure according to local guidelines.  If no immediate response, change to pulsed doses of methylprednisolone 1 g/d and add MMF, infliximab or anti-thymocyte globulin. |  |
|  | **3**  Severe symptoms with mild exertion |  |  |
|  | **4**  Is life-threatening |  |  |

Abbreviations: AE, adverse event; ALT, alanine aminotransferase; AST, aspartate aminotransferase; BSA, body surface area; CHF, congestive heart failure; CK, creatine kinase; CK-MB, creatine kinase cardiac isoenzyme; ECG, electrocardiogram; INR, international normalized ratio; LFT, liver function test; MMF, mycophenolate mofetil; MYHA, New York Heart Association; T4, thyroxine; TB, tuberculosis; TFT, thyroid function test; TSH, thyroid‑stimulating hormone; U&E, urea and electrolytes; ULN, upper limit of normal.

REFERENCES:

Brahmer JR, Drake CG, Wollner I, et al. Phase I Study of Single-Agent Anti–Programmed Death-1 (MDX-1106) in Refractory Solid Tumors: Safety, Clinical Activity, Pharmacodynamics, and Immunologic Correlates. J Clin Oncol, 2010, 3167-3175.
